# Characterization and mitigation of gene expression burden in mammalian cells

**DOI:** 10.1038/s41467-020-18392-x

**Published:** 2020-09-15

**Authors:** Timothy Frei, Federica Cella, Fabiana Tedeschi, Joaquín Gutiérrez, Guy-Bart Stan, Mustafa Khammash, Velia Siciliano

**Affiliations:** 1 Department of Biosystems Science and Engineering (D-BSSE), ETH Zürich, Mattenstrasse 26, Basel, 4058 Switzerland; 2 Istituto Italiano di Tecnologia-IIT, Largo Barsanti e Matteucci, Naples, 80125 Italy; 3 University of Genoa, Genoa, 16132 Italy; 4 Department of Bioengineering and Centre for Synthetic Biology, Imperial College London, London, SW7 2AZ UK

**Keywords:** miRNAs, Genetic circuit engineering, Synthetic biology

## Abstract

Despite recent advances in circuit engineering, the design of genetic networks in mammalian cells is still painstakingly slow and fraught with inexplicable failures. Here, we demonstrate that transiently expressed genes in mammalian cells compete for limited transcriptional and translational resources. This competition results in the coupling of otherwise independent exogenous and endogenous genes, creating a divergence between intended and actual function. Guided by a resource-aware mathematical model, we identify and engineer natural and synthetic miRNA-based incoherent feedforward loop (iFFL) circuits that mitigate gene expression burden. The implementation of these circuits features the use of endogenous miRNAs as elementary components of the engineered iFFL device, a versatile hybrid design that allows burden mitigation to be achieved across different cell-lines with minimal resource requirements. This study establishes the foundations for context-aware prediction and improvement of in vivo synthetic circuit performance, paving the way towards more rational synthetic construct design in mammalian cells.

## Abstract (web summary)

The design of genetic networks in mammalian cells is still slow and often fails. Here the authors show that miRNA-based incoherent feedforward loop circuits can be used to alleviate cellular burden.

## Introduction

Mammalian synthetic biology facilitates the study of diverse biological processes including gene regulation^1^, developmental patterns^2^, evolution^3^, and cancer progression^4^. More recently, it has gained clinical relevance, offering powerful new tools for the engineering of recombinant protein-producing cells^5^ and for the creation of novel cell-based therapies for clinical use^6–8^. Prior to cell engineering, the synthetic parts and the behavior of their resulting devices are tested and characterized via transient transfection in the desired mammalian cell lines. However, often the discrepancy between expected and actual behavior leads to numerous design–build–test–learn iterations^9,10^, which are particularly expensive and time consuming^11^ in mammalian cells.

At the core of the problem is the poor predictability of gene expression^10^ in transfected cells arising from the dependence of gene expression on the cellular context. In particular, the often overlooked dependence of exogenous genetic circuits on limited host resources that are shared with endogenous pathways frequently leads to unanticipated and counterintuitive circuit behaviors^12^. In bacterial cells, substantial progress towards increasing the predictability of gene expression has been made by showing that exogenous genetic material imposes a significant burden, resulting in decreased growth rates and degraded cellular performance^13^. This has been attributed to the diversion of the pool of resources available for gene expression^14,15^ towards transcription and translation of the newly introduced synthetic payloads. These observations prompted the development of models that consider gene expression in a resource-limited context^16–19^ and led to approaches for mitigating the impact of resource burden in bacteria^20,21^. Analogous studies in *Saccharomyces cerevisiae* showed that transcription and translation are limiting processes^22^. For example, the use of potent transactivators—such as the DOX-inducible rtTA—causes a squelching shortage of general transcription factors for native gene expression in yeast^23^. In mammalian cells, while performance shortcomings of synthetic circuits due to transactivator dosage and plasmid uptake variation^24^ have been observed, a deeper understanding of the problem of resource burden and methods for its mitigation are still missing. Competition for endogenous resources can have detrimental effects on basic and translational biology. For instance, in studies based on transient DNA expression, genes that are used to normalize the results might be subject to resource-dependent expression coupling (e.g. protein levels measured by flow cytometry are usually normalized to the expression levels of the transfection marker, which is also used as a measure of transfection efficiency).

**Fig. 1:**
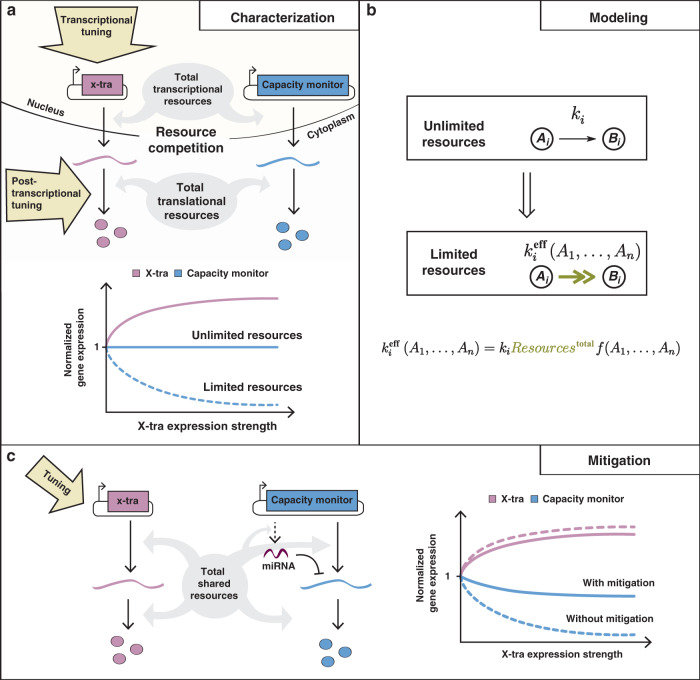
Resource sharing and the origin of gene expression burden. **a** Characterization of gene expression burden. Expression of independent exogenous genes impacts on host cellular resources. Thus, perturbations in one gene’s expression (hereby named X-tra) affect the expression of a second gene (hereby named capacity monitor). **b** Modeling of gene expression in a resource-limited environment. Modeling of gene expression is generally performed under the assumption of unlimited resources. A simple framework enables the straightforward transformation of such a model to a system that incorporates resources explicitly. The transformation involves a simple function that scales the original reaction rate. **c** Mitigation of gene expression burden. A simple microRNA-based circuit motif is capable of mitigating the burden-induced coupling of X-tra and the capacity monitor. It should be noted that the dynamic range of X-tra also slightly decreases as a consequence of mitigation. However, as it will be discussed in the results section and shown in Supplementary Fig. 19, the absolute expression of X-tra is higher with mitigation.

Here, we investigate the burden imposed by transiently expressed synthetic circuits on host cells (Fig. 1). Through the design of genetic constructs that allow us to uncouple transcription and translation processes, we separately study transcriptional and translational burden caused by cellular resource sharing. In particular, we engineer several regulatory circuits composed of a tunable load, called X-tra (eXtra Transgene), which we genetically express in the host cell in varying amounts. We then measure the impact of this tunable load on a “sensor” gene, which we refer to as the capacity monitor (Fig. 1a). We demonstrate in different mammalian cell lines that the sharing of transcriptional and translational resources in the host cell can tightly couple otherwise independently co-expressed synthetic genes and lead to trade-offs in their expression (Fig. 1a). To enhance the predictability of synthetic devices in mammalian cells, we explicitly incorporate these load-sharing effects in a general mathematical model in which we replace the rates of resource-dependent reactions with adjusted effective rates (Fig. 1b). This framework follows ideas originally used to capture the competitive interaction of multiple inhibitors with an enzyme^25^ and has been applied to describe shared cellular resources in previous studies^16–19,26^. We demonstrate the usefulness of this modeling framework by showing that it successfully recapitulates the non-monotonic dose–response behavior of a simple inducible gene expression system observed in Lillacci et al.^24^. Additionally, we investigate the role of post-transcriptional regulators, like RNA-binding proteins (RBPs) and microRNAs (miRNAs), in mitigating the impact of burden-induced coupling and find that both are able to reallocate resources, making them candidates for use in burden-mitigation circuits. Using these observations, and guided by our modeling framework, we identify the incoherent feedforward loop (iFFL) as a network topology that is particularly effective at resource burden mitigation, and then we use endogenous and synthetic miRNA regulation to engineer iFFL-based, burden-mitigating synthetic circuits (Fig. 1c). While miRNA-based iFFL circuits have been previously constructed and proposed to buffer gene expression against noise^27,28^ and fluctuations in external inducer concentration^29^, in this study we demonstrate that they also act to rescue the expression level of genes of interest despite changes in available cellular resources due to the loading effects of transgene constructs (Fig. 1c). Our findings pave the way to more realistic output predictions and optimal synthetic construct design in mammalian cells.

## Results

### Genetic circuits compete for limited shared resources

**Fig. 2:**
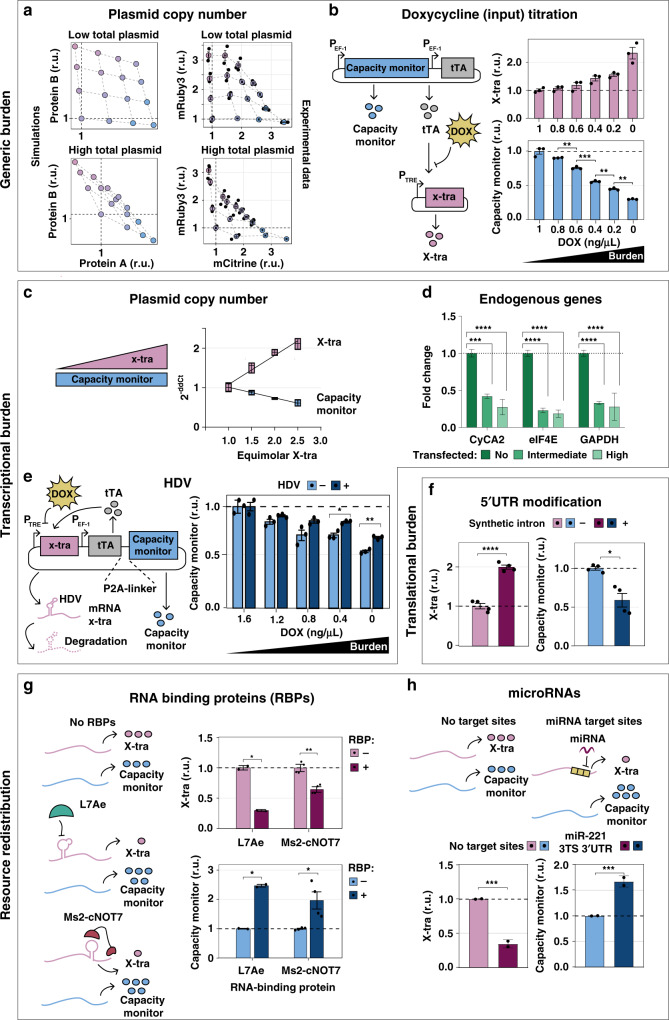
Burden imposed by genetic circuits in mammalian cells. **a** Left: As the total plasmid amount increases, the total expression plateaus. Right: Titration of two plasmids expressing the fluorescent proteins mCitrine and mRuby3 from EF1α promoters in ratios from 1:4 to 4:1 (total of 50 ng, top right; or 500 ng of DNA, bottom right). *N* = 3 biological replicates. Source data are provided as a Source Data file. **b** Two plasmids were co-transfected, one constitutively expressing capacity monitor and tTA from a strong constitutive promoter and the other expressing X-tra from a tTA responsive promoter. Capacity monitor levels counterbalance the increase in X-tra expression. Flow cytometry data are normalized to the expression at maximal Dox. *N* = 3 biological replicates. Source data are provided as a Source Data file. **c** mRNA quantification of X-tra and a capacity monitor expressed at different molar ratios. As the X-tra increases, the mRNA levels of the capacity monitor decreases. *N* = 4 biological replicates. qPCR analysis was performed 48 h post-transfection and data show fold change ± SE. Source data are provided as a Source Data file. **d** Cells transfected with a plasmid expressing two fluorescent proteins from a bidirectional promoter were sorted according to high, intermediate, or no fluorescence (Supplementary Fig. 4) for mRNA extraction. mRNA levels expressed from endogenous genes decrease in cells with intermediate and high fluorescence. *N* = 3 biological replicates. Data show fold change ± SE. Individual values are plotted in Supplementary Fig. 28. Source data are provided as a Source Data file. **e** Capacity monitor levels are higher with an HDV ribozyme rapidly degrading the capacity monitor mRNA than with an inactive mutant, suggesting a sequestration of transcriptional resources. *N* = 3 biological replicates (*N* = 2 for HDV−, 1.6 ng/μL DOX). Source data are provided as a Source Data file. **f** The synthetic intron shows higher X-tra levels compared to a control and leads to reduced capacity monitor levels. *N* = 4 biological replicates. Source data are provided as a Source Data file. **g** Repressed X-tra expression leads to increased capacity monitor levels. *N* = 2 biological replicates for L7Ae and *N* = 4 for Ms2-cNOT7. Source data are provided as a Source Data file. **h** When X-tra is downregulated by miR-221 endogenously expressed in HEK293T cells, the capacity monitor levels increase. All flow cytometry data were acquired 48 h post-transfection and are plotted as mean ± SE. SE standard error, r.u. relative units. *N* = 2 biological replicates. Source data are provided as a Source Data file. Unpaired two-sided *T*-test. *P* value: ****<0.0001, ***<0.0005, **<0.005, *<0.05.

We reasoned that competition for finite cellular resources would introduce an indirect coupling in the expression levels of two otherwise independently expressed genes. To test this, we co-transfected HEK293T cells with two constitutively expressed fluorescent proteins mCitrine and mRuby3 driven by EF1α promoters, in molar ratios ranging from 1:4 to 4:1, for a total of 50 ng (low) or 500 ng (high) of encoding plasmid (Fig. 2a). The competition for limited resources is expected to shape gene expression as presented in Fig. 2a, according to the modeling framework that will be introduced in Fig. 4a (model described in Supplementary Note 2). As expected, the total amount of 500 ng of encoding plasmids results in a dramatic drop of encoded-gene expression as compared to 50 ng (Fig. 2a, right). Furthermore, in both experimental conditions mCitrine and mRuby3 fluorescence levels are negatively correlated; the higher the amount of expressed mCitrine, the lower that of mRuby3 and vice versa (Fig. 2a, right); this correlation was also more severe for 500 ng of transfected plasmid than for 50 ng.

We demonstrated that the negative correlation is promoter independent: using a CMV and a PGK promoter^30^ that have different expression strength in HEK293T and H1299 (Supplementary Fig. 1a), we observed analogous outcomes (Supplementary Fig. 1b–e). Further, by combining different molar ratios of mCitrine and mRuby3 encoding plasmids driven by two promoters of different strengths (EF1α or EFS) a similar behavior to Fig. 2a was observed (Supplementary Fig. 2). Finally, as many synthetic circuits rely on tunable gene expression, we next tested resource competition on transcriptional inducible systems, by modulating X-tra repression with a Doxycycline (Dox)-repressed promoter (Fig. 2b) at different concentrations of Dox (from 0 to 1 μg/mL) while keeping capacity monitor amounts constant (Fig. 2b, left). Consistent with previous results, we observed that increased repression of X-tra corresponds to increased capacity monitor levels (Fig. 2b, right).

To exclude any bias of fluorescent protein expression on resource competition, we transfected a plasmid encoding a human codon optimized variant of the bacterial *σ*-factor sigW in increasing amounts with a fixed concentration of the mCitrine capacity monitor plasmid, and demonstrated similar behavior to fluorescent protein expression (Supplementary Fig. 3).

Finally, to avoid any experimental confounds as the source of our observations, we showed that neither cell seeding nor nutrient supply had any apparent effect on the expression levels of the two genes, one of which was titrated whereas the second was held at a constant copy number (Supplementary Fig. 4).

These proof-of-concept experiments demonstrate that (i) gene expression in mammalian synthetic circuits is connected even in the absence of direct regulation and (ii) expression of exogenous genes is limited by cellular resource availability.

### Transcriptional and translational resources are limiting

Since several different resource pools could be responsible for the observed effects described above, we set out to characterize the individual contributions of transcriptional and translational resource limitation to cellular burden in HEK293T and H1299 cells (Fig. 2). To evaluate potential limitations in transcriptional resources and the consequent gene competition for mRNA expression, we quantified mRNA levels in cells expressing X-tra/capacity monitor molar ratios from 1:1 to 2.5:1 in H1299 cells for a total of 500 ng of plasmid DNA (corresponding protein data in Supplementary Fig. 8a). We observed that as the X-tra mRNA increased, the capacity monitor mRNA levels decreased (Fig. 2c), supporting the hypothesis that shared transcriptional resources are indeed a limiting factor in mammalian synthetic gene co-expression.

To investigate whether the expression of endogenous genes is also affected by heterologous genetic payloads, we transfected H1299 cells with a plasmid encoding for EGFP and mKate under the control of a bidirectional promoter. We then sorted transfected cells according to high and intermediate levels of fluorescent markers as well as non-transfected cells (absence of fluorescence) (Supplementary Fig. 5). We then quantified the mRNA levels of three endogenous genes (CyCA2, eIF4E, GAPDH, Fig. 2d, Supplementary Fig. 28). Notably, in transfected cells that express high and intermediate levels of EGFP and mKate, the expression of CyCA2, eIF4E, and GAPDH decreases when compared to the non-transfected population. We also measured the mRNA levels of CyCA2, eIF4E, and GAPDH in cells transfected with X-tra/capacity monitor molar ratios from 1:1 to 2:1 and observed a progressive, albeit not dramatic decrease with higher amounts of X-tra when compared to the 1:1 ratio (Supplementary Fig. 6). Of note, in the latter experiment cells were not sorted before mRNA extraction.

To provide further support to the observations on transcriptional burden on exogenous genes (Fig. 2c), we implemented a genetic circuit that can selectively overload the transcriptional resource pool without sequestering translational resources. The system is based on the self-cleaving hepatitis delta virus (HDV) ribozyme, which ensures that most of the transcribed mRNA is cleaved and thus destabilized (Fig. 2e, left). The circuit is composed of a single plasmid with two transcriptional units (TUs). One TU contains a tTA transcription factor co-expressed with the mRuby3 (capacity monitor) via the P2A peptide, driven by a constitutive promoter. The second TU includes the HDV-X-tra expression regulated by the TRE promoter. In this setup, Dox can be used to modulate the amount of burden imposed, similar to what was already shown in Fig. 2b.

We compared this circuit to a catalytically inactive mutant of the HDV ribozyme in HEK293T cells. As expected, we observed that when the HDV ribozyme is inactive, X-tra protein levels increase with decreasing amounts of Dox (Supplementary Fig. 7, top pale pink bar), whereas those of the capacity monitor decrease (Fig. 2e, bottom pale blue bar). In contrast, when the HDV ribozyme is active, X-tra expression is strongly reduced and only minorly increasing with lower Dox concentrations (Supplementary Fig. 7, top dark purple bar). Here, the capacity monitor levels decrease to a smaller extent than in the previous condition, supporting the observations in Fig. 2c that transcriptional resources are limited to a certain extent (Fig. 2e, dark blue bar). Interestingly, the expression levels of the capacity monitor with active HDV ribozyme are higher compared to the inactive mutant (Supplementary Fig. 7, bottom dark blue bar). We suggest that, assuming that the X-tra mRNA with an active HDV ribozyme is decapped and rapidly degraded, it is likely to sequester fewer translational resources, which should result in higher expression of the capacity monitor.

Transcriptional resource pool sharing is therefore at least partially responsible for the described gene expression trade-offs, and translational resources may represent an additional bottleneck to the overall expression of synthetic genes. We confirmed this hypothesis by adding a synthetic intron^31^ in the 5′ untranslated region (UTR) of the X-tra fluorescent protein (Fig. 2f, top). The synthetic intron enhances translation by augmenting mRNA export from the nucleus to the cytoplasm^31^ and therefore imposes specific translational load. Indeed, we observed higher expression of X-tra in HEK293T (Fig. 2f) and H1299 (Supplementary Fig. 8b) cell lines in the presence of a synthetic intron, accompanied by lower capacity monitor levels, confirming that resources employed for translational regulation are also limiting. Thus our data collectively indicate that exogenous genes compete for resources both at the transcriptional and translational levels, overall imposing a gene expression burden on mammalian cells.

Since one of the goals in synthetic biology is output predictability, reproducibility, and robustness, gene expression burden is a key issue to address. We reasoned that post-transcriptional and translational regulators, such as RBPs and miRNAs, may free up cellular resources^32^ by repressing target mRNA translation or inducing its degradation. If true, they could be exploited in more robust circuit topologies to reduce gene expression load, resulting in improved performance and predictability of engineered circuits. Therefore, we tested two RBPs, L7Ae and Ms2-cNOT7 (refs. ^33,34^), as well as endogenous miRNAs, miR-221 and miR-31, in HEK293T (Fig. 2g, h) and H1299 (Supplementary Fig. 8c, d) respectively. For each system, a fluorescent protein encoding mRNA targeted by either RBPs or miRNAs (X-tra) was co-expressed with a second, constitutively expressed fluorescent readout (capacity monitor). L7Ae binds the 5′UTR of the X-tra mRNA inhibiting its translation, whereas Ms2 binds target sites (TS) in the 3′UTR of the X-tra transcript, allowing cNOT7 to cut the polyA tail to destabilize the target mRNA^33^. We consistently observed in both cell lines that X-tra downregulation by RBPs results in increased levels of the capacity monitor (Fig. 2g, Supplementary Fig. 8c).

miRNAs operate by either translation inhibition or mRNA degradation, according to complete^35^ or partial^36^ complementarity to the mRNA target. To evaluate the effect of miRNA regulation on cellular resource reallocation, we placed three perfect complementary TS in the 3′UTR of X-tra, which respond to the endogenous miR-221 and miR-31 highly expressed in HEK293T and H1299 cells. The capacity monitor expression levels increased when the X-tra mRNA was downregulated by miRNAs, as compared to controls lacking miRNA TS (Fig. 2h, Supplementary Fig. 8d).

To further demonstrate that the burden imposed by synthetic circuits is cell-type independent, we performed the same set of experiments of Supplementary Fig. 1d and Fig. 2f–h in U2OS, HeLa, and CHO-K1 cells, obtaining similar results (Supplementary Figs. 9–11). Interestingly, even CHO-K1 cells, which are the workhorses of the biopharmaceutical industry due to their high productive capability^37^ show cellular burden. Redistribution of resources was also observed by the RBPs L7Ae and MS2-cNot7 and the highly expressed endogenous miR-221 and miR-21 in U2OS and HeLa/CHO-K1 cells, respectively.

These results confirm that post-transcriptional regulators can redistribute intracellular resources and, importantly, that this phenomenon is cell-context independent. The extent of negative correlation between X-tra and capacity monitor expression, as well as the amount of repression by post-transcriptional regulators, differs across cell lines; this could be the consequence of several factors, such as the relative abundance of transcriptional, post-transcriptional, and translational resources.

A major advantage of miRNAs over RBPs is that they are endogenously expressed and cell line specific. Thus, their expression does not impose an additional burden, and since several thousand endogenous miRNAs with different TS are naturally present in mammalian cells^38^, the design space is rather large, giving rise to a tremendous number of circuits that can be easily tailored to the cell/tissue of interest. Based on the results presented here, we envision that genetic circuits that mitigate resource competition via miRNAs may be designed for any mammalian cell line with a very broad set of potential applications.

### Characterizing the effect of miRNAs on resource distribution

**Fig. 3:**
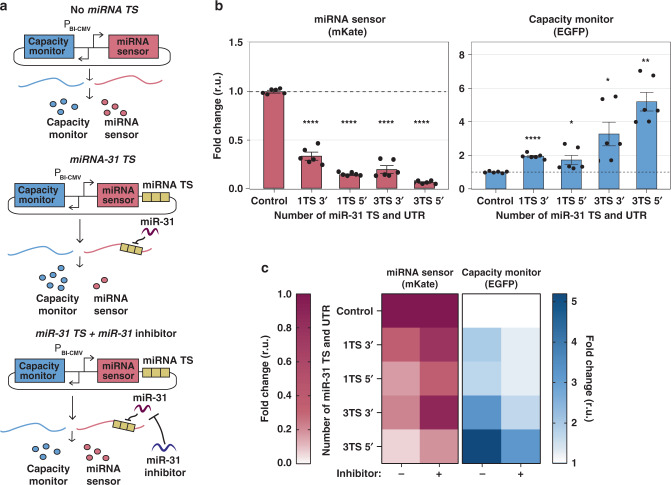
Impact of miRNA target sites number and location on burden. **a** Schematics of experimental design to infer miRNA-mediated cellular resources redistribution. EGFP (capacity monitor) and mKate (miRNA sensor) are encoded on the same bidirectional CMV promoter plasmid. One or 3 TS for miR-31 (TS) are added either in the 3′ or 5′UTR of mKate. Control: no miR-31 TS. Hypothesis: in the absence of miR-31 regulation, capacity monitor and miRNA sensor are expressed to a certain level (top). In the presence of miR-31, lower miRNA sensor levels correlate with higher capacity monitor expression (middle). This condition is reversed by an miR-31 inhibitor (bottom). **b** Fold change of miRNA sensor and capacity monitor protein levels compared to control (set to 1). EGFP increases up to fivefold with the strongest downregulation of mKate (3 TS 5′UTR). Flow cytometry data were acquired 48 h post-transfection and are plotted as mean ± SE. SE standard error, r.u. relative units. *N* = 6 biological replicates. Source data are provided as a Source Data file. Unpaired two-sided *T*-test. *P* value: ****<0.0001, **<0.005, *<0.05. **c** When miR-31 activity was impaired by a miR-31 inhibitor, the rescue of mKate expression corresponds to reduced EGFP levels, whereas both fluorescent proteins do not vary in the control. The heatmaps represent the fold change derived by flow cytometry data, calculated as the ratio between the geometric mean of six biological replicates and the corresponding geometric mean in the control condition. Source data are provided as a Source Data file. Bar plots and statistical analysis are reported in Supplementary Fig. 12.

We sought to characterize the correlation between miRNA-mediated downregulation and resource redistribution by building a library of miRNA sensors for miR-31, which is endogenously expressed in H1299 lung cancer cells^39^. The miRNA sensor is composed of the fluorescent reporter mKate with or without miR-31 TS, encoded along with the capacity monitor (EGFP) on a single plasmid with a bidirectional promoter (Fig. 3a). The library includes 0, 1, or 3 fully complementary miR-TS in the 3′ or 5′UTR of mKate.

Similar to what was previously observed (Supplementary Fig. 8d), when the miRNA sensor’s levels decrease as a consequence of miR-31 regulation, the expression of the capacity monitor increases. The strongest repression was achieved with 3 TS in the 5′UTR and was accompanied by corresponding higher capacity monitor levels (Fig. 3b). Conversely, when we rescued mKate expression by a miR-31 inhibitor (Fig. 3c, left and Supplementary Fig. 12, red bars), the capacity monitor levels decreased (Fig. 3c, right and Supplementary Fig. 12, dark blue bars) demonstrating that miRNA sensor and capacity monitor levels are linked. Interestingly, the effect of the miRNA inhibitor was more pronounced with TS placed in the 3′UTR. Synthetic miRNA inhibitors bind to endogenous miRNAs in an irreversible manner^40^, but differences in their action (e.g. when TS are placed in the 3′ versus 5′UTR), as well as mechanistic insights into these differences, are still missing.

To confirm that miRNA-mediated resource redistribution is independent of experimental setting and plasmid design, we encoded the miRNA sensor and capacity monitor on two separate plasmids. Similar to previous results, miRNA sensor and capacity monitor were negatively correlated (Supplementary Fig. 13a), suggesting that cellular burden and miRNA-dependent resource reallocation are a common challenge and solution respectively. Downregulation of the miRNA sensor was also confirmed by qPCR (Supplementary Fig. 13b). Finally, when the miR-31 sensor was transfected in low miR-31 cell lines such as U2OS and HEK293T, neither the miRNA sensor nor the capacity monitor levels varied (Supplementary Fig. 14), further confirming the miRNA-dependent resource reallocation.

We showed in Fig. 2h and Supplementary Figs. 8d, 9d, 10d and 11d that miRNA-dependent resource reallocation is observed across different cell lines, by expressing cell-specific miRNA sensors which include 3 TS in the 3′UTR. We then built a library of sensors with different numbers and locations of TS for miRNA-221 and -21 which are highly expressed in U2OS and HeLa cells, respectively. We also confirmed here that miRNA sensor and capacity monitor are inversely correlated, consistent with our observations in H1299 cells (Supplementary Figs. 15 and 16).

Overall these data show that miRNAs can be used to develop resource-aware plasmid-designs harboring burden-mitigating circuit topologies, and that the number and location of TS can be tuned to achieve desired protein expression levels.

### A resource-aware model framework

In order to provide a better understanding of our results, we developed a general resource-aware model, which offers a simple and convenient framework for extending existing models of biochemical reactions allowing them to incorporate the effects of shared limited resources.

**Fig. 4:**
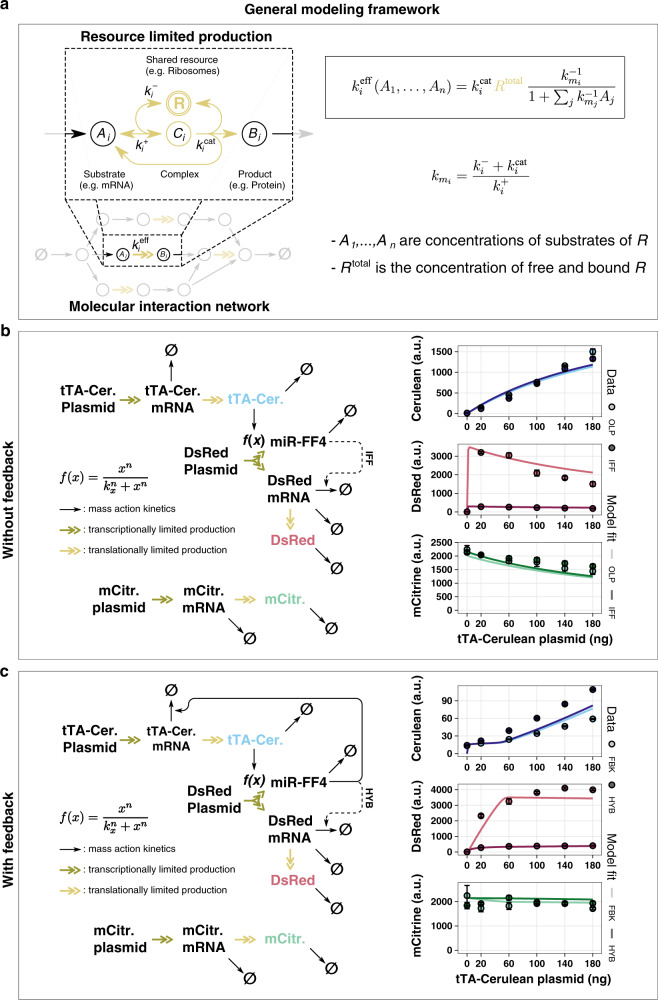
A resource-aware mathematical modeling framework. **a** General framework for transforming molecular interaction network models. Existing models of molecular interaction networks can be transformed to include shared limiting resources by substituting *k*_*i*_, the reaction rate of a resource-limited production, with *k*^eff^_*i*_. Shown above an exemplary resource-limited production are the detailed interactions between the substrate and the shared resource. **b** Limited shared resources reproduce non-monotonous dose response in open-loop and incoherent feedforward circuit topologies. On the left, a graphical representation of a model for both the open-loop (OLP) and incoherent feedforward (IFF) topologies from Lillacci et al.^24^. Transcriptional activation is modeled by a Hill-type function. The solid arrows denote reactions assumed to follow the law of mass action. The model incorporates resources as introduced in panel **a**. These reactions are depicted as double-headed arrows. The model was fit to data obtained by transiently transfecting HEK293T cells with increasing amounts of plasmid encoding tTA-Cerulean. The data and the fit are shown on the right. **c** Limited shared resources reproduce non-monotonous dose-response in feedback and hybrid circuit topologies. The model shown on the left is the same as in panel **b** with an additional negative feedback from miR-FF4 to tTA-mRNA. These topologies correspond to the feedback (FBK) and hybrid (HYB) topologies from Lillacci et al.^24^ The activation of gene expression by tTA-Cerulean is modeled by a Hill-type function as shown in the center. Reactions with double-headed arrows denote resource-limited production reactions as introduced in panel **a**. Solid arrows are assumed to follow the law of mass action. The model was fit to experimental data obtained from transient transfections with increasing amounts of plasmid encoding tTA-Cerulean. A description of the models can be found in Supplementary Note 4 and the parameter values obtained by fitting are summarized in Supplementary Table 7. Data were obtained 48 h after transfection and are plotted as mean ± SE. SE standard error. *N* = 3 biological replicates. Source data are provided as a Source Data file.

Figure 4a illustrates an overview of the framework. The main idea is to replace the rates of reactions that involve a shared resource with an effective reaction rate that captures the reduced availability of that resource due to the presence of competing genes. To create a distinction between regular reactions and resource-limited ones, we use double-headed reaction arrows to denote resource-limited reactions as illustrated at the bottom of Fig. 4a. This double-headed arrow summarizes the set of intermediate interactions shown in more detail at the top left of Fig. 4a. Here, the substrate *A*_*i*_ binds resource *R* with rate *k*^+^_*i*_ to form the complex *C*_*i*_. This reaction is also assumed to be reversible with rate *k*^−^_*i*_. With a rate *k*^cat^_*i*_ the complex gives rise to the product *B*_*i*_, while also freeing up both the substrate *A*_*i*_ and the resource *R*. We assume that the total amount of resource, *R*^total^, is conserved and remains constant at the time scale of the considered reactions. Considering all possible substrates that require resource *R* and assuming that *C*_*i*_ is in quasi-steady state, the rate for resource-limited production can be expressed as *k*^eff^_*i*_, shown in the top right corner of Fig. 4a. A more detailed derivation can be found in Supplementary Note 1. *k*^eff^_*i*_ is a function of the total amount of resources and the current concentration of all substrates competing for this resource. This expression can be readily used to substitute all reaction rates that involve shared and limited resources.

To demonstrate the effectiveness of our modeling framework, we extend the models of different circuit topologies introduced in Lillacci et al.^24^ to include limited resources and show that the resulting extended models recapitulate the previously unexplained non-intuitive experimental observations.

The four topologies considered in Lillacci et al.^24^ were split into two groups based on the presence of negative feedback from the fluorescent protein DsRed to the transcriptional activator (tTA). The first group consisted of the open-loop (OLP) and incoherent feedforward (IFF) topologies. In both these circuits, the constitutively expressed transcriptional transactivator, fused to the fluorescent protein Cerulean (tTA-Cer), activates the expression of the fluorescent protein DsRed. Furthermore, the gene of DsRed intronically encodes the synthetic miRNA FF4 (miR-FF4). In the IFF topology, the matched target of this miRNA is present in the 3′UTR of the *DsRed* gene. This target is replaced by a mismatched target for the miRNA FF5 in the OLP. These detailed interactions are depicted here in Fig. 4b, left side. To observe potential shifts in the allocation of resources, we generated dose–response curves by increasing the amount of transfected tTA-Cer plasmid, while the other two plasmids, containing DsRed and the constitutively expressed fluorescent transfection reporter mCitrine, were held constant. As can be seen from the model fit, plotted as a solid line in the data graph, the extended model reproduces the non-monotonic behavior of the dose responses (Fig. 4b, right).

The second group of topologies considered by Lillacci et al.^24^ consisted of the feedback (FBK) and the FBK + IFF hybrid (HYB) topologies. In addition to all the interactions described for the OLP and IFF circuits, the FBK and the HYB circuits possess miR-FF4 targets in the 3′UTR of the *tTA-Cer* gene, which introduces negative feedback. Furthermore, the FBK and HYB differ from each other by the presence of a matched target for miR-FF4 in the HYB topology, which introduces incoherent feedforward and is replaced by a mismatched FF5 target in the FBK circuit. All the interactions are illustrated in detail in Fig. 4c, left. The dose–response curves for the two circuits were obtained as described above. Again, the fit of the extended model to the data captures its rather unexpected behavior (Fig. 4c, right).

Lastly, we also apply our framework to model the gene expression systems presented in Figs. 2b, e, g and 3b. The resulting model fits are shown in Supplementary Fig. 17. The models are described in Supplementary Note 6 and the parameter values obtained by fitting are summarized in Supplementary Tables 13–16.

Our simple framework adapts existing models of gene expression to include pools of shared and limited resources. We show that it can be used to provide an explanation for unintuitive dose responses in tTA-based circuits. With this framework as a tool, we believe that performance issues attributed to gene expression burden can be addressed head-on in the design phase of circuit-building, thereby reducing the need for costly subsequent build-test-learn iterations.

### Mitigating burden with iFFL circuits

**Fig. 5:**
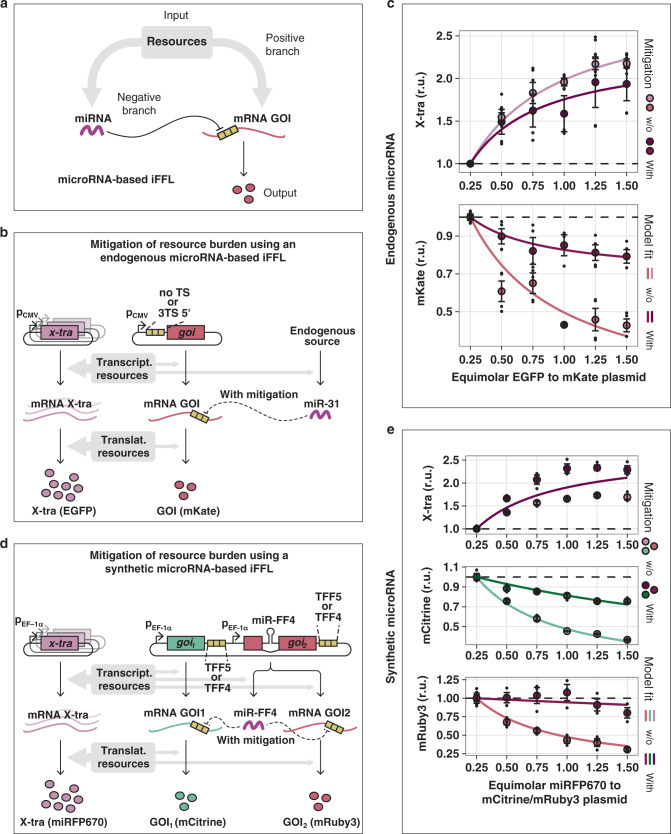
Mitigating the effects of resource limitation with microRNA-based iFFL. **a** The microRNA-based incoherent feedforward loop (iFFL) motif. **b** Mitigation system based on endogenous microRNA. At high copy number of the X-tra, resources are drawn away from the production of the GOI and miR-31. By sensing the resource availability and repressing the GOI less when there are fewer resources, the miRNA reduces the effect of limited resources. **c** Two plasmids were co-transfected into H1299 cells which respectively express the *X-tra* and *GOI* genes (EGFP and mKate respectively (**b**)), and the molar ratio of the X-tra:GOI plasmid was progressively increased. The presence of miR-31 TS in mKate 5′UTR mitigates effects due to resource sharing. The parameter values obtained by fitting are summarized in Supplementary Table 8. *N* = 3 biological replicates. **d** Mitigation system based on synthetic miRNA. In the presence of many copies of the *X-tra* gene, resources are drawn away from the production of both the GOIs and the miR-FF4. Due to lower production of miR-FF4 the GOIs are less repressed. This compensates for the reduced availability of resources. **e** A plasmid encoding both the fluorescent protein mCitrine and an intronic microRNA expressed from the *mRuby3* gene (GOI_1_, GOI_2_ and miR-FF4 (**d**)) was co-transfected into HEK293T cells with increasing amounts of a plasmid expressing the *X-tra* gene (miRFP670 (**d**)). The impact of resource limitation on both GOIs was reduced when they contained three miR-FF4 targets in their 3′UTRs compared to when they contained three mismatched miR-FF5 targets. The parameter values obtained by fitting are summarized in Supplementary Table 9. *N* = 3 biological replicates. Source data are provided as a Source Data file. A description of the models can be found in Supplementary Note 5. Flow cytometry data were acquired 48 h post-transfection and are plotted as mean ± SE. SE standard error, r.u. relative units.

We implemented a strategy that exploits miRNA to reduce the indirect coupling between co-expressed genes. In particular, we took advantage of the fact that miRNA production also requires (pre-translational) cellular resources, therefore acting as a sensor for resource availability. Because of this, it is possible to reduce the coupling between genes co-expressed via a common resource pool by introducing miRNA-mediated repression of those genes (as long as the miRNA itself is also affected by the same resource pool). Since both the miRNA and the miRNA-repressed gene are affected by the availability of resources, miRNA-mediated repression implements an iFFL similar to previously published circuits^24,29,41^ (Fig. 5a). Interestingly, this iFFL-based circuit constitutes a biological implementation of the miRNA circuit proposed by Zechner et al.^42^. In this setting, the miRNA can be interpreted as an estimator of its cellular context (e.g. amount of free resources) and acts to filter out this context, thereby minimizing its impact on the output of interest.

We explored this strategy for an endogenously expressed miRNA (Fig. 5b, c) and a synthetic miRNA encoded on a plasmid (Fig. 5d, e). More specifically, Fig. 5b describes a strategy that exploits endogenous miRNAs to reduce the coupling of a gene of interest (GOI) to the expression level of other genes, introduced by the limitation in resources. Implementation of this strategy only requires adding the TS of an endogenous miRNA to the 5′UTR of the gene of interest (mKate). In our experimental setup, when the copy number of a second gene *(X-tra)* is increased, resources are drawn away from the expression of mKate and allocated to the expression of X-tra. The shift in resource allocation is expected to also affect miR-31, which acts as a capacity monitor. This leads to a reduction in the repression of mKate, effectively compensating for the burden imposed by the co-expression of the *X-tra* gene.

To demonstrate this mitigation approach experimentally, we co-transfected H1299 cells with increasing amounts of EGFP (X-tra), along with a constant amount of mKate (GOI) that either includes (for mitigation) or omits (no mitigation) three miR-31-TS in the 5′UTR. As expected, the expression level of X-tra approached saturation as the plasmid copy number increased, both for the targeted and non-targeted GOI variants (Fig. 5c). In agreement with previous results, the expression of the non-targeted GOI strongly decreased with increased expression of X-tra. Conversely, the decrease in expression of the targeted GOI was only about a third of that of the non-targeted variant, indicating improved adaptation to changes in resource availability (Fig. 5c and Supplementary Fig. 18). This observation was also captured well by a model of the system that explicitly considered resources, as described in the previous section. It should be noted that while the relative dynamic output range of X-tra is slightly reduced (fold change of 1.94× with mitigation versus 2.18× without mitigation (Fig. 5c), our data show that the absolute levels of X-tra increases about 2× in the presence of miR-31-based iFFL, de facto benefiting from this network topology (Supplementary Fig. 19). Analogously, miR-221-iFFL circuits specific for U2OS and HEK293T cells^43^ (Supplementary Fig. 21) show improved robustness to burden imposed by increasing exogenous gene load (Supplementary Figs. 20 and 22). Models used for fitting and the resulting parameter values are summarized in Supplementary Note 5 and Supplementary Tables 10 and 11.

Importantly, the delivery of genetic payloads also affects the expression of endogenous genes (CyCA2, elF4E, and GAPDH), as shown in Fig. 2d. We then sought to compare the expression of the same endogenous genes in the presence or absence of miR-31 sensor in H1299 cells. After 48 h from transfection of EGFP and mKate on a bidirectional plasmid, with mKate either including (miRNA sensor) or not (noTS) TS for miR-31, we sorted cells according to high, intermediate, or absence of fluorescence expression (Supplementary Fig. 23a) and performed qPCR. Curiously, we observed that in cells transfected with miR-31 sensor, the decrease in the expression of the endogenous genes was much lower than in its absence (Supplementary Fig. 23c). Furthermore, the expression of endogenous genes was inversely proportional to the levels of fluorescent proteins (Supplementary Fig. 22b). Thus, the lower expression of endogenous genes due to the burden imposed by exogenous payloads is counteracted by the miR-31-sensor. To investigate whether the use of endogenous miRNAs may impair the regulation of native targets, we measured the expression of SATB2 mRNA, a natural target of miR-31 (ref. ^44^) in cells transfected with miR-31-sensor versus the noTS control, and observed no difference between the two conditions (Supplementary Fig. 24).

Motivated by our desire to achieve portability across cell lines and multiple-output regulation, we implemented and tested a synthetic miRNA-iFFL circuit that tunes two GOIs (Fig. 5d). Similar to the endogenous case, the genes of interest, *mCitrine* (*GOI*_*1*_) and *mRuby3* (*GOI*_*2*_), encode TS for the miRNA-FF4 in their 3′UTRs. In contrast to endogenous miRNA expression, however, here the miRNA is expressed intronically from *GOI*_*2*_. In this way, the circuit forms a self-contained unit that can be easily transferred between cell types.

We co-transfected HEK293T cells with a plasmid encoding constitutively expressed miRFP670 (X-tra) and a plasmid composed of two TUs, each expressed under the constitutive promoter EF1α (Fig. 5d). The first TU encodes mCitrine, whereas the second drives mRuby3. Furthermore, the 3′UTR of mCitrine and mRuby3 contained either three TS for the synthetic miRNA-FF4 or three mismatched miR-FF5 TS (negative control). The miRNA-FF4 was intronically encoded in the *mRuby3* gene. Identically to the endogenous case, the amount of X-tra plasmid was increased while keeping the GOIs plasmid constant. Again, expression of X-tra increased and approached saturation with increasing molar amounts and consequently, the non-targeted variants of the GOIs decreased (TFF5 in Fig. 5e). Conversely, the expression of the targeted variants (TFF4 in Fig. 5e) decreased to a lesser extent than the non-targeted ones, analogously to what was observed for endogenous miRNAs, albeit with lower efficiency. Finally, to demonstrate the portability of the device we tested the approach in mouse embryonic stem cells (Supplementary Fig. 25). Here, adaptation to shifts in resource availability was similar to the endogenous miRNA-based regulation (Fig. 5c). The model used for fitting and the resulting parameter values are summarized in Supplementary Note 5 and Supplementary Table 12. Thus, we showed that also in entirely synthetic systems, adaptation to shifts in resource availability was achieved. To ensure that the observed mitigation was not caused by a higher tolerance to changes in availability at lower expression levels, we showed analytically using the described modeling framework that the normalized expression at lower levels was more sensitive to burden (Supplementary Note 3).

Indeed, mitigation comes at the cost of the maximal achievable expression levels for the capacity monitor. Moreover, tuning the iFFL circuit to become even less sensitive to changes in available resources will necessarily further limit the maximal expression. This trade-off is intrinsic to the iFFL mitigation strategy. Nevertheless, these results suggest that our approach can be used to mitigate resource-mediated coupling of gene expression despite cell-to-cell variability, demonstrating the portability and broad applicability of our findings. Our results demonstrate that iFFL circuits can mitigate burden from transgene expression in mammalian cells. Importantly, by using miRNAs one can either opt for endogenous miRNAs to specifically tailor a circuit to a desired cell line or create a portable circuit by using a synthetic miRNA such as miR-FF4.

## Discussion

Our study demonstrated that the sharing of limited cellular resources represents a general bottleneck for the predictability and performance of transiently transfected synthetic circuits in mammalian cells, with important consequences for mammalian synthetic biology and biotechnology applications. Due to resource limitations, transient heterologous gene expression results in the coupling of independent exogenous genes and affects the expression of endogenous ones. We presented a detailed characterization of the distinct contributions of transcriptional and translational processes to resource competition and showed that RBPs and miRNAs can redistribute cellular resources thereby alleviating burden. To get a deeper understanding of the mechanisms behind gene expression coupling, we described a modeling framework that captures the indirect interdependence of gene expression in a resource-limited context. Our resource-aware model successfully recapitulated the non-intuitive behavior of the dose responses for the family of controllers described in Fig. 4, demonstrating its potential to aid the design of circuits that are less prone to burden effects.

The modeling framework also suggested that an iFFL is a particularly well-suited circuit motif for mitigating burden effects. The iFFL itself is one of the core gene regulatory motifs in biology, and unsurprisingly it has served as inspiration for many synthetic genetic circuits that exploit its adaptation properties^24,29,41,45^. In this study we adopt a miRNA implementation of iFFL circuits for the purpose of burden mitigation. Previously, synthetic miRNA-based iFFLs have been demonstrated to increase robustness to gene dosage variability^24,41^ and external perturbations^29^. In contrast to synthetic miRNAs, endogenous miRNAs have seen far more limited use in synthetic circuits (e.g. as inputs to synthetic cell-type classifiers^46,47^). Regardless of their origin, miRNA-based iFFL circuits were shown here to decouple the expression of both exogenous and endogenous genes. We speculate that this positive effect is attributed to the freeing up of translational resources, leading to an increase in the expression of proteins involved in the transcription of endogenous genes. At the same time, as already proposed in Gambardella et al.^48^, the downregulation of mKate by miRNAs may lead to a “queueing effect” for the degradation of the other mRNAs, similar to what was shown with two independent proteins tagged for degradation by the proteasome^49^.

An implementation of iFFL could alternatively be achieved using RBPs (e.g. L7Ae and Ms2-cNOT7), or using endoribonucleases as is done in a concurrent study by Jones et al.^50^. Here, we opted for a miRNA-based approach (both endogenous and synthetic) due to several considerations. RBPs impose additional burden, limiting their suitability to mitigate burden itself, while miRNAs are endogenously or intronically expressed with the GOI, thus channeling a negligible amount of resources. To achieve minimal load as in our endogenous miRNA-based iFFL, the RBP alone, or the iFFL should be integrated into the genome. However, a single-copy integration may not guarantee burden mitigation, whereas multiple copy integration may constitute itself a new source of burden. Such systems would need to be tested to assess their usefulness for burden mitigation. Moreover, RBPs rely on specific binding sites that are not as easy to tune as miRNA TS. Lastly, miRNA circuits do not use genetic components that derive from different organisms, circumventing potential toxicity and immunogenicity concerns that could limit their application in medical therapy^51,52^.

At the same time, miRNAs offer several inherent benefits. Specifically, iFFL circuits that exploit endogenous miRNAs enable cell-type specificity^46,53^, whereas synthetic miRNAs enable portability of circuits across different cell lines. Furthermore, flexibility at the sequence level allows scaling up to many orthogonally operating circuits. The specificity of a miRNA can be easily engineered to target any synthetic or endogenous gene without the need to engineer the target itself^54–56^ (programmability). Finally, tunability of repression strength can be easily achieved both through the number and the placement of the targets, and can be used to enhance adaptation to variations in resource availability. It should be noted that stronger repression will yield lower expression levels of the gene of interest (GOI, Fig. 5c). This trade-off is unavoidable, and is an inherent limitation to all implementations of iFFL-based burden-mitigation circuits, including endoRNase implementations.

A potential limitation of miRNA-based iFFL circuits is the diversion of endogenous miRNAs from native targets to synthetic ones. Although this is not what we observe in our miR-31 iFFL (Supplementary Fig. 24) this may however give rise to an inevitable trade-off similar to what has been observed for competing endogenous RNA (ceRNA). ceRNAs are known to naturally regulate other RNAs by competing for miRNA-binding. To attempt to remedy this, one could use partially complementary TS, which would decrease the affinity of the miRNA to the target and diminish the competition. However this would make the system less efficient and potentially decrease the mitigation effect. Alternatively, the incorporation of multiple TS that respond to different highly expressed miRNAs would distribute the competition between multiple miRNAs and reduce the detrimental effects on their native targets.

Besides iFFLs, negative feedback motifs^24,57–60^ can also be used to mitigate resource burden, as was shown in a series of studies in *Escherichia coli*^13,17,20,21,61^. While negative feedback circuits possess well-established robustness properties, iFFL circuits have several advantages for burden mitigation. In particular, iFFL circuits are considerably simpler to implement and easier to tune than negative feedback circuits, which usually require more components and can become dynamically unstable if not properly designed and tuned. In terms of dynamic response, iFFL circuits are also generally faster in rejecting disturbances like a sudden change in resource availability. Indeed iFFL regulation responds to the disturbance itself, while negative feedback begins to act only after the impact of the disturbance on the regulated output has been detected.

In this study we characterized the contribution of transcriptional and translational processes to resource competition in mammalian cells. In yeast, it was previously reported that squelching, a shortage of general transcription factors, is responsible for the evolutionary breakdown of synthetic gene circuits following exogenous gene expression under a rtTA-responsive promoter^23^. However, a deeper understanding of similar effects in mammalian cells is currently lacking. For example, the activation domain of tTA, VP16, interacts with essential components of the transcription machinery such as TFIIB, TFIID, TFIIH, and dTAFII40 (ref. ^62^), whose abundances or sub-compositions are unknown and may vary widely across cells^63,64^. Uncovering the key players responsible for gene coupling and endogenous genes’ dysregulation will enable the implementation of even more robust and resource-aware solutions to mitigate gene expression burden.

Ultimately, the goal of gene circuit engineering is the creation of cell lines that stably express circuits of interest. Although the presented work focused on the effects of limited resources as induced by transient transfection, it would be natural to investigate if similar effects also occur in the context of genomic integration of highly expressed genes. Moreover, while using a transiently transfected capacity monitor enables the quantification of cellular expression capacity by providing a comparative measure of the geometric mean of free resources in a burdened population relative to a minimally burdened baseline population, stable integration of the capacity monitor would permit a more direct measure in terms of arithmetic mean of free cellular resources (Supplementary Note 7).

Understanding the impact of resource availability during the engineering of biological systems will have important consequences for biological studies and for improved mammalian cell engineering. For example, studies of biological functions that employ perturbations by exogenous gene expression often lack accuracy and exhibit highly variable results due to less-than-optimal genetic circuit designs. Using burden-aware designs, cell therapies that rely on finely tuned expression and secretion of therapeutic molecules can now be engineered with resource-aware circuits. Our findings suggest that, when choosing a host cell line, one of the key factors to consider should be its transcriptional and translational capacity^22^ not only in terms of productivity but also in terms of the ability of the cells to maintain their fitness while performing their engineered function. Our study presents a portable design capable of enhancing the insulation of transgene expression and will thus contribute to the development of robust-by-design mammalian synthetic circuits, with important implications for basic science and applications in industrial biotechnology and medical therapy.

## Methods

### Cell culture

HEK293T, U2OS, and HeLa cells (all from the ATCC) used in this study were maintained in Dulbecco’s modified Eagle medium (DMEM, Gibco); H1299 (ATCC) were maintained in Roswell Park Memorial Institute medium (RPMI, Gibco); CHO-K1 were maintained in minimum essential medium α (α-MEM, Gibco). All media were supplemented with 10% FBS (Atlanta BIO), 1% penicillin/streptomycin/l-glutamine (Sigma-Aldrich), and 1% non-essential amino acids (HyClone). HEK239T cells (ATCC, strain number CRL-3216) used for part of this study were maintained in DMEM (Sigma-Aldrich or Gibco) supplemented with 10% FBS (Sigma-Aldrich), 1× GlutaMAX (Gibco) and 1 mM Sodium Pyruvate (Gibco). E14 mouse embryonic stem (mES) (a kind gift from Dr. Maaike Welling) cells were grown in DMEM (Gibco) supplemented with 15% FBS (PAN Biotech; specifically for ES cell culture), 1% penicillin/streptomycin (Sigma-Aldrich), 1% non-essential amino acids (Gibco), 2 mM l-glutamine (GlutaMAX; Gibco), 0.1 mM beta-mercaptoethanol (Sigma-Aldrich), and 100 U/mL Leukemia inhibitory factor (LIF; Preprotech). At every passage the media was additionally supplemented with fresh CHIR99021 to 3 μM and PD0390125 to 1 μM to support naïve pluripotency (2*i* conditions^65^). All labware used was coated with 0.1% gelatin (prepared ourselves) prior to plating the ES cells. The cells were maintained at 37 °C and 5% CO_2_.

### Transfection

Transfections were carried out in a 24-well plate for flow cytometry analysis or in a 12-well plate format for flow cytometry and qPCR analysis run on the same biological replicates (Supplementary Table 1). Transfections for Fig. 2d and Supplementary Figs. 5 and 22 were carried out in 6 cm dishes. H1299, HeLa, U2OS, HEK293T, and CHO-K1 cells were transfected with Lipofectamine® 3000 (ThermoFisher Scientific) according to the manufacturer’s instructions and 300 ng total DNA (500 ng in Fig. 2c, d and Supplementary Figs. 1, 8a, 9a, 10a, and 11a) in 24-well plates. DNA and transfection reagents were scaled up according to the Lipofectamine® 3000 manufacturer’s instructions. miR-31 inhibitor (Invitrogen™ *mir*Vana™ miRNA Inhibitors) was co-transfected using the same method as for DNA (Fig. 3c).

HEK293T cells used for experiments shown in Figs. 2a, b, e, 4 and 5e were plated approximately 24 h before transfection at 62,500–75,000 cells per well in 24-well plates. The transfection solution was prepared using polyethylenimine (PEI) “MAX” (Mw 40,000, Polysciences, Inc.) in a 1:3 (μg DNA to μg PEI) ratio with a total of 500 ng of plasmid DNA per well. Both DNA and PEI were diluted in Opti-MEM I reduced serum media (Gibco) before being mixed and incubated for 25 min prior to addition to the cells. E14 mouse embryonic stem cells were transfected using Lipofectamine® 2000 (ThermoFisher Scientific) in a 1:3 (μg DNA to μg Lipofectamine® 2000) with 300 ng of plasmid DNA per well. The transfection was performed on cells in suspension immediately after plating at approximately 30,000 cells per well. All wells were coated with 0.1% gelatin before the addition of the cells.

### Flow cytometry and data analysis

H1299, HEK293T, U2OS, HeLa, and CHO-K1 cells were analyzed with a BD Facsaria™ cell analyzer (BD Biosciences) or BD Celesta™ cell analyzer (BD Biosciences) using 488 and 561 lasers. For each sample >20,000 singlet events were collected and fluorescence data were acquired with the following cytometer settings: 488 nm laser and 530/30 nm bandpass filter for EGFP, 561 nm laser and 610/20 nm filter for mKate. Cells transfected in 12-well plates were washed with DPBS, detached with 100 μL of Trypsin-EDTA (0.25%), and resuspended in 600 μL of DPBS (Thermo Fisher). Two hundred microliters of cell suspension were used for flow cytometry and 400 μL for RNA extraction. HEK293T used for experiments shown in Figs. 2a, b, e, 4 and 5e cells were measured 48 h after transfection on a BD LSRFortessa™ Special Order and Research Product (SORP) cell analyzer. mCitrine fluorescence was excited via a 488 nm laser and was detected through a 530/11 nm bandpass filter. mRuby3 was excited via a 561 nm laser and measured through a 610/20 nm bandpass filter. miRFP670 was excited at 640 nm and measured through a 670/14 nm bandpass filter. E14 mES cells were measured 48 h after transfection on a Beckman Coulter CytoFLEX S flow cytometer. mCitrine fluorescence was excited using a 488 nm laser and was detected through a 525/40+OD1 bandpass filter. mRuby3 was excited with 561 nm laser light and measured through a 610/20+OD1 bandpass filter. miRFP670 was excited at 638 nm and measured through a 660/10 bandpass filter. The cells were collected for measurement by washing with DBPS (Sigma-Aldrich or Gibco) and detaching in 70–180 μL of Accutase solution (Sigma-Aldrich). For each sample between 10,000 and 200,000 singlet events were collected. Fluorescence intensity in arbitrary units (a.u.) was used as a measure of protein expression. For each experiment a compensation matrix was created using unstained (wild type cells), and single-color controls (mKate/mCherry only, EGFP only or mCitrine only, mRuby3 only, miRFP670 only). Live cell population and single cells were selected according to FCS/SSC parameters (Supplementary Figs. 26 and 27). Data analysis was performed with Cytoflow or a custom R script. Data fitting was performed using Mathematica’s NonlinearModelFit function and the InteriorPoint method.

### Cell sorting

H1299 cells used for the experiment shown in Fig. 2d were trypsinized from 6 cm dishes and counted. They were then centrifuged at 500*g* for 5 min and resuspended at a concentration of 5 mln/mL in sorting buffer (PBS 1× + 3 mM EDTA + 0.8% Trypsin + 1% FBS). Cells were sorted with a BD FACSMelody™ cell sorter according to their fluorescence levels (Supplementary Fig. 5). In total, 150,000 cells per gate were collected.

### DNA cloning and plasmid construction

Plasmid vectors carrying gene cassettes were created using In-Fusion HD cloning kit (Clonetch), Gibson Assembly, via digestion and ligation or using the yeast toolkit (YTK)^66^ with custom parts for mammalian cells. Gibson Assembly master mixes were created from Taq DNA Ligase (NEB), Phusion High-Fidelity DNA Polymerase (NEB), and T5 Exonuclease (Epicentre) in 5× isothermal buffer (Supplementary Table 6). Ligation reactions were performed in 1:2–5 molar ratios of plasmid backbone:gene insert starting with 50–100 ng of vector backbone digested with selected restriction enzymes. Assemblies using the YTK were performed according to the original publication^66^. Newly created constructs were transformed into XL10-Gold or TOP10 *E. coli* strains.

For plasmids with miRNA TS, the target sequences were selected using miRBase database (http://www.mirbase.org/) and are listed in Supplementary Table 4. List of oligos used to clone endogenous miRNAs TS are listed in Supplementary Table 3. All plasmids were confirmed by sequencing analysis and deposited to addgene.

To perform western blot analysis, an His-tag composed of six Histidine residues was inserted after the start codon of mKate encoding plasmids.

### mRNA extraction and reverse transcription

RNA extraction was performed with E.Z.N.A.® Total RNA Kit I (Omega Bio-tek). The protocol was followed according to manufacturer’s instructions and RNA was eluted in 30 L of RNAse free water. RNA samples were conserved at −80 °C.

PrimeScript RT Reagent Kit with gDNA Eraser—Perfect Real Time (Takara) was used according to the manufacturer’s instructions. The protocol was performed on ice in a RNAse free environment to avoid RNA degradation. A negative control without PrimeScript RT Enzyme Mix I was always prepared to investigate genomic DNA contamination.

### qPCR

Fast SYBR Green Master Mix (ThermoFisher Scientific) was used to perform qPCR of cDNAs obtained from 500 ng of RNA and diluted 1:5. Samples were loaded in MicroAmp™ Fast Optical 96-Well Reaction Plate (0.1 mL) and the experiment was carried out with a CFX96 Touch Real-Time PCR Detection System (BioRad) machine. Each well contained 20 μL of final volume (7 μL SYBR Green Master Mix, 10 μL ddHO, 1 μL of each primer, 1 μL of template). Also, a control without template (blank) was set. Primers were designed to amplify a region of 60–200 bp (Supplementary Table 5) and with a temperature of annealing between 50 °C and 65 °C. Data were analyzed using the comparative Ct method according to the Applied Biosystems Protocols.

### Statistics and reproducibility

Each experiment was repeated independently at least twice with similar results, with the exception of Supplementary Fig. 2 and condition w/o Mitigation, 1.5 equimolar EGFP to mKate plasmid in Supplementary Fig. 20. All models used for parameter fitting are contained in Supplementary Note 4–6. The obtained parameter values are summarized in Supplementary Tables 7–16.

### Reporting summary

Further information on research design is available in the Nature Research Reporting Summary linked to this article.

## Supplementary information

Supplementary Information

Reporting Summary

## Data Availability

All relevant data are included as Source Data and/or are available from the corresponding author on reasonable request. Plasmid sequences are deposited on AddGene and GenBank under the accession codes specified in Supplementary Table 2. Strains and plasmids used in this study are available from the corresponding author on reasonable request. The miRNA target sites were obtained from the miRBase database (http://www.mirbase.org/) and are listed in Supplementary Table 4. Source data are provided with this paper.

## Source data

Source Data

## Code availability

The authors are confident that the conclusions do not strongly depend on the particular choice of analysis software. Nevertheless, the code used for automated analysis and fitting is available on reasonable requests from the corresponding authors.
