# Supplementary material for: Characterization and mitigation of gene expression burden in mammalian cells
Source: Nat Commun. 2020 Sep 15;11:4641. doi: 10.1038/s41467-020-18392-x (PMC7492461; doi:10.1038/s41467-020-18392-x)
Supplement: Supplementary file 1 — Supplementary Information [file 41467_2020_18392_MOESM1_ESM.pdf]

Supplementary Information

Frei T±, Cella F±, Tedeschi F, Gutierrez J, Stan GB, Khammash M*, Siciliano V*.

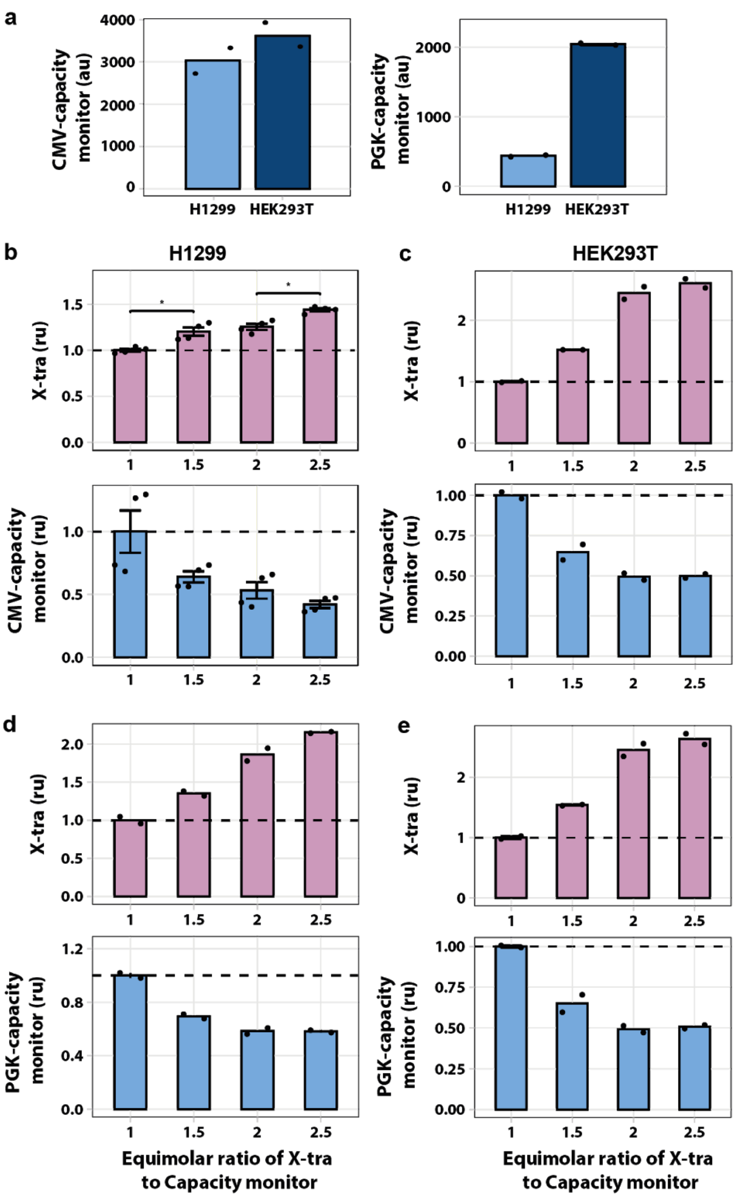

Supplementary Figure 1. Relation of X-tra and capacity monitor expression in H1299 and HEK293T cell lines using CMV and PGK promoter. (a) Levels of fluorescence driven by the same promoter (CMV or PGK) differ across cell lines. Data show absolute units of capacity monitor detected by flow cytometry in 1:1 molar ratio transfection. N=2 biological replicates. Source data are provided as a Source Data file. Flow cytometry results of H1299 (N=4 biological replicates). Source data are provided as a Source Data file. **(b)** and HEK293T (N=2 biological replicates). Source data are provided as a Source Data file. **(c)** cells co-transfected with fixed amount of CMV-mKate (capacity monitor) PGK-EGFP (X-tra) (molar ratio from 1:1 to 1:2.5). Flow cytometry results of H1299 (N=2 biological replicates) **(d)** and HEK293T (N=2 biological replicates). Source data are provided as a Source Data file. **(e)** cells co-transfected with fixed amount of mKate (capacity monitor) under PGK promoter regulation and increasing amount of EGFP (X-tra) under CMV promoter regulation (molar ratio from 1:1 to 1:2.5). N=2 biological replicates. Source data are provided as a Source Data file. Data show the mean fluorescence normalized to its value at a plasmid molar ratio of 1. Error bars represent the standard error, SE. au: arbitrary units. ru: relative units. Unpaired two-sided T-test. p-value: * < 0.05.

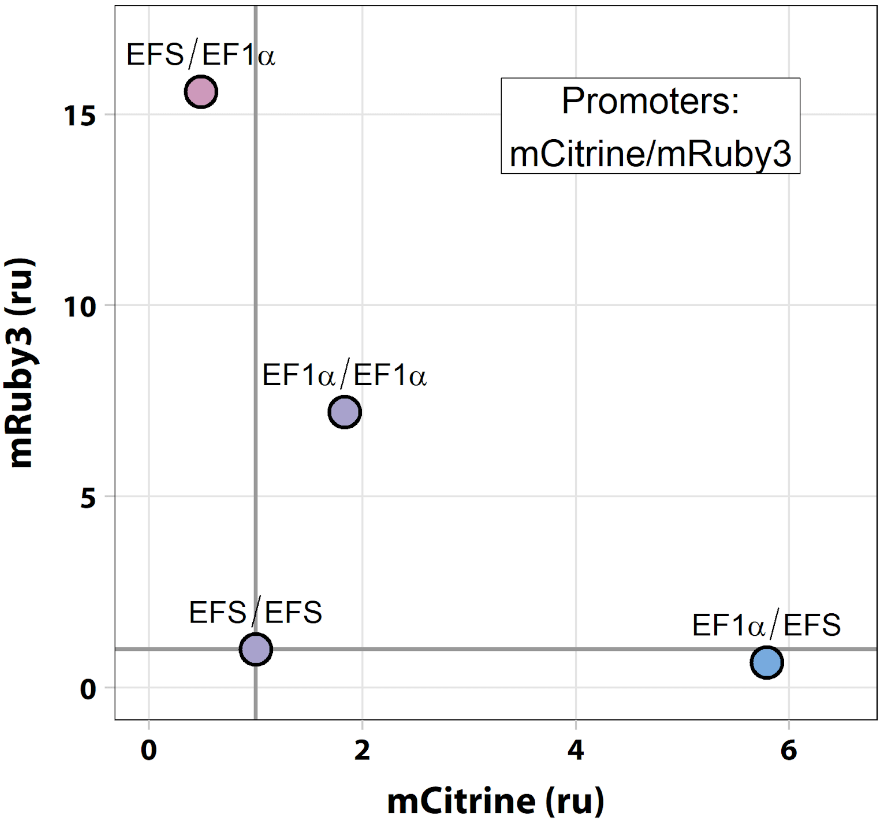

Supplementary Figure 2. Promoter expression strength indirectly affects expression of co-transfected genes. Plasmids expressing the fluorescent protein mCitrine and mRuby3 from a strong (EF-1α) or a medium strength EF-1α short (EFS) promoter were co-transfected in several molar ratio combinations. The expression levels for both mCitrine and mRuby3 were normalized by the data obtained from the weakest promoters pair (EFS/EFS). Similar to **Fig. 2a**, there is a negative correlation between the expression strength of one protein and the promoter strength of the other gene. Of note, when strong promoters drive both proteins, the global expression levels drop as already suggested by **Fig. 2a**. Source data are provided as a Source Data file. Data was acquired 48 hours after transfection and is plotted as fluorescence normalized to the EFS/EFS sample. ru: relative units.

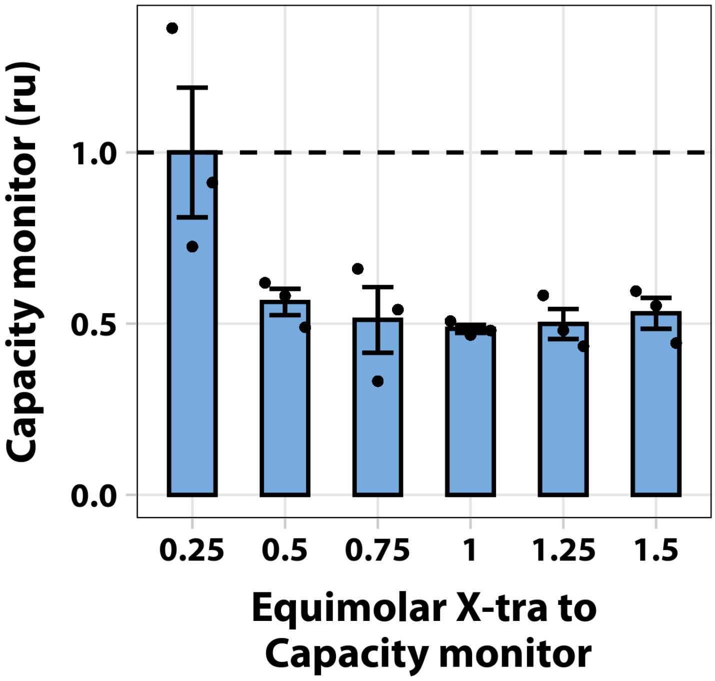

Supplementary Figure 3. Non-fluorescent protein genes also compete for cellular resources. A plasmid encoding a human codon optimized variant of the bacterial σ-factor sigW was co-transfected in increasing amounts with a fixed concentration of the mCitrine capacity monitor plasmid. Data were acquired 48 hours after transfection and are plotted as mean fluorescence normalized to the lowest equimolar ratio. Error bars represent the standard error, SE. ru: relative units. N=3 biological replicates. Source data are provided as a Source Data file.

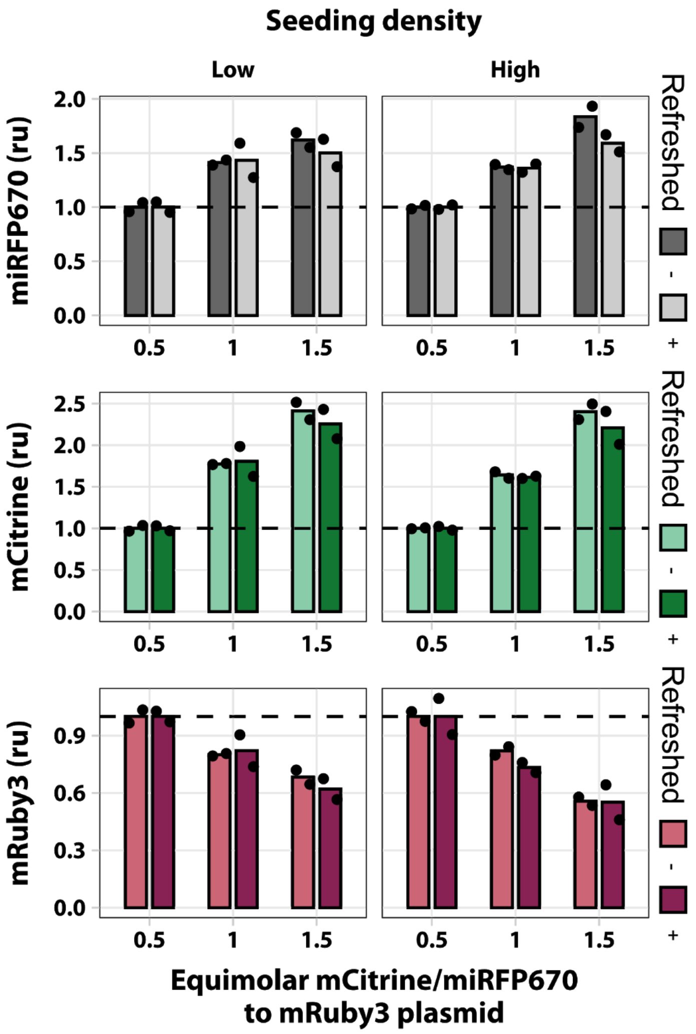

Supplementary Figure 4. Assessing nutrient starvation and cell seeding density as potential impacts on limited resources. In this experimental setting, cells were co-transfected with two plasmids. The first plasmid, which was provided at incremental levels, is composed of two transcriptional units (TU), one consisting of a strong promoter driving the expression of mCitrine (hEF1a), the other driving the expression of miRFP670 under a weak promoter (SV40). The second plasmid encodes for mRuby3 under a strong constitutive promoter (hEF1a).
HEK293T cells were seeded at $5e^{4}$ (low) and $7.5e^{4}$ (high) cells/well to assess seeding density effects. To investigate the effects of nutrient starvation, we refreshed the medium in two out of four wells per condition. Data were collected 48 hours post transfection and represent the mean fluorescence intensity of the three fluorescent proteins normalized to the 0.5 equimolar ratio condition. Error bars represent the standard error, SE. N=2 biological replicates. Source data are provided as a Source Data file.

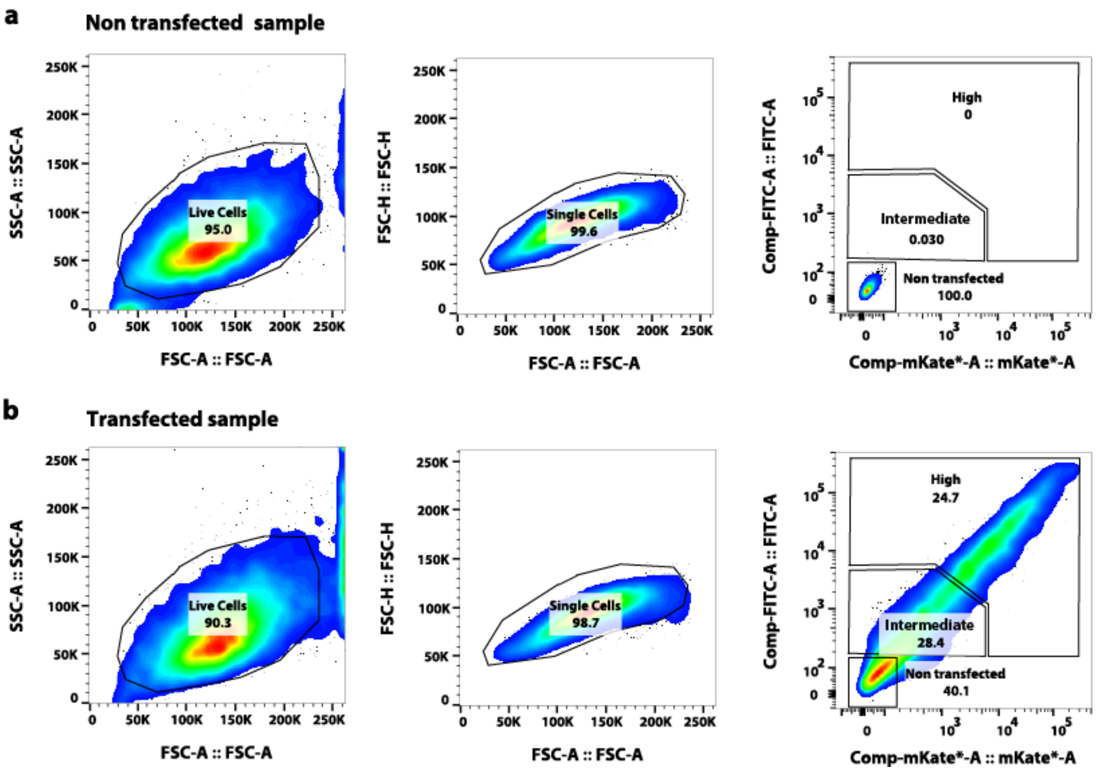

Supplementary Figure 5. Sorting strategy. H1299 cells were transfected with a plasmid encoding the fluorescent proteins EGFP and mKate, expressed from a bidirectional promoter. Cells were sorted by fluorescence intensity 48 hours post-transfection to collect non-transfected, intermediate and high transfected cells from the same transfection plate. **(a, b)** First, gates to select live and single cells were determined (left and middle plots). Then, the threshold for fluorescent intensity was set using a non-transfected sample as reference **(a, right)**. The two additional gates to collect intermediate and high transfected cells were created as shown in the plots **(a, b)** on the right.

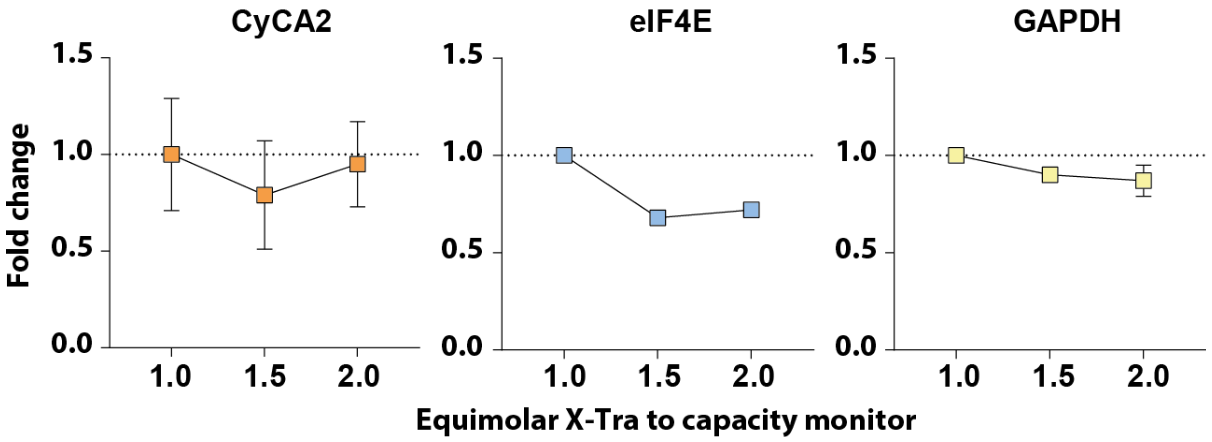

Supplementary Figure 6. Effect of *X-tra* titration on endogenous genes. We measured CyCA2, eIF4E and GAPDH mRNA levels by qPCR in the samples shown in **Fig. 2c** at 1.0, 1.5 and 2.0 molar ratios. Data represent the mean value normalized to the equimolar ratio of 1.0. Error bars represent the standard error, SE. N=4 biological samples for CyCA2 and GAPDH. N=2 biological samples for eIF4E. Source data are provided as a Source Data file.

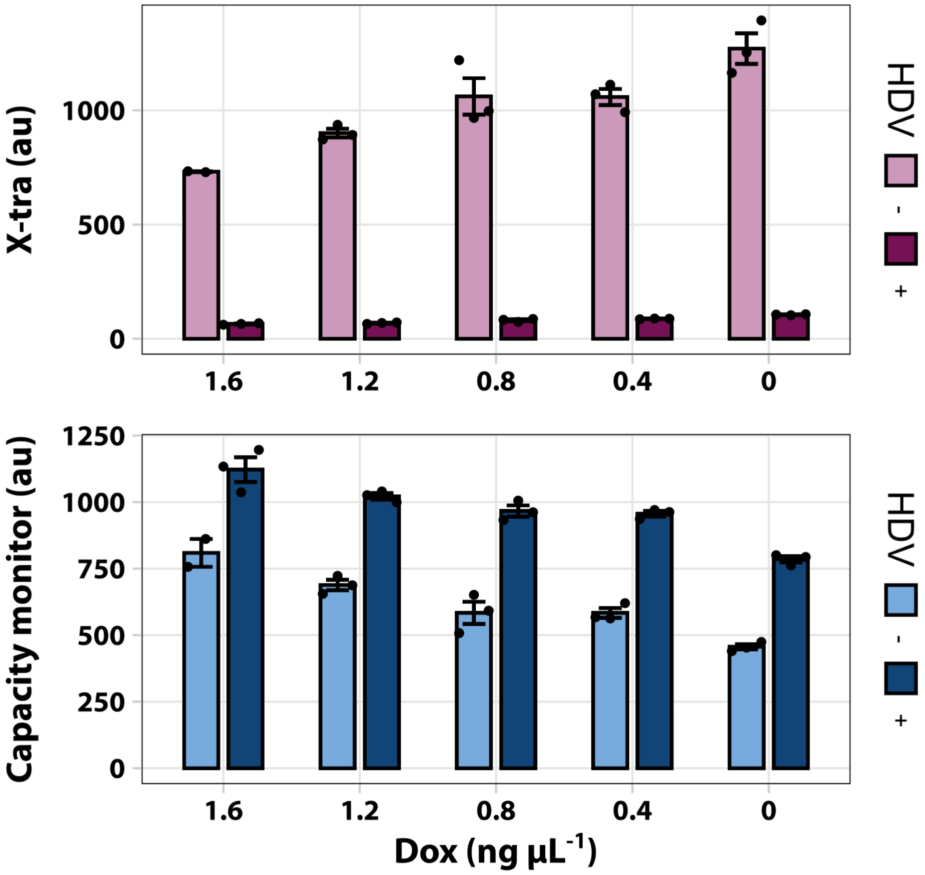

Supplementary Figure 7. Fluorescence data of Fig. 2e shown in arbitrary units. In this experimental setting Dox represses *X-tra* transcription. Thus, the lower Dox, the higher the *X-tra* levels, and as a consequence, the lower the *capacity monitor* levels. The HDV-dependent mRNA decapping and degradation of *X-tra* should consume less translational resources, which is consistent with the higher expression of the *capacity monitor* (dark blue bars) as compared to the inactive mutant (pale blue bars). Data was acquired 48 hours after transfection and is plotted as mean fluorescence intensity +/- SE. SE: standard error. N=3 biological replicates (N = 2 for HDV -, 1.6 ng/µL DOX). Source data for **Fig. 2e** are provided as a Source Data file.

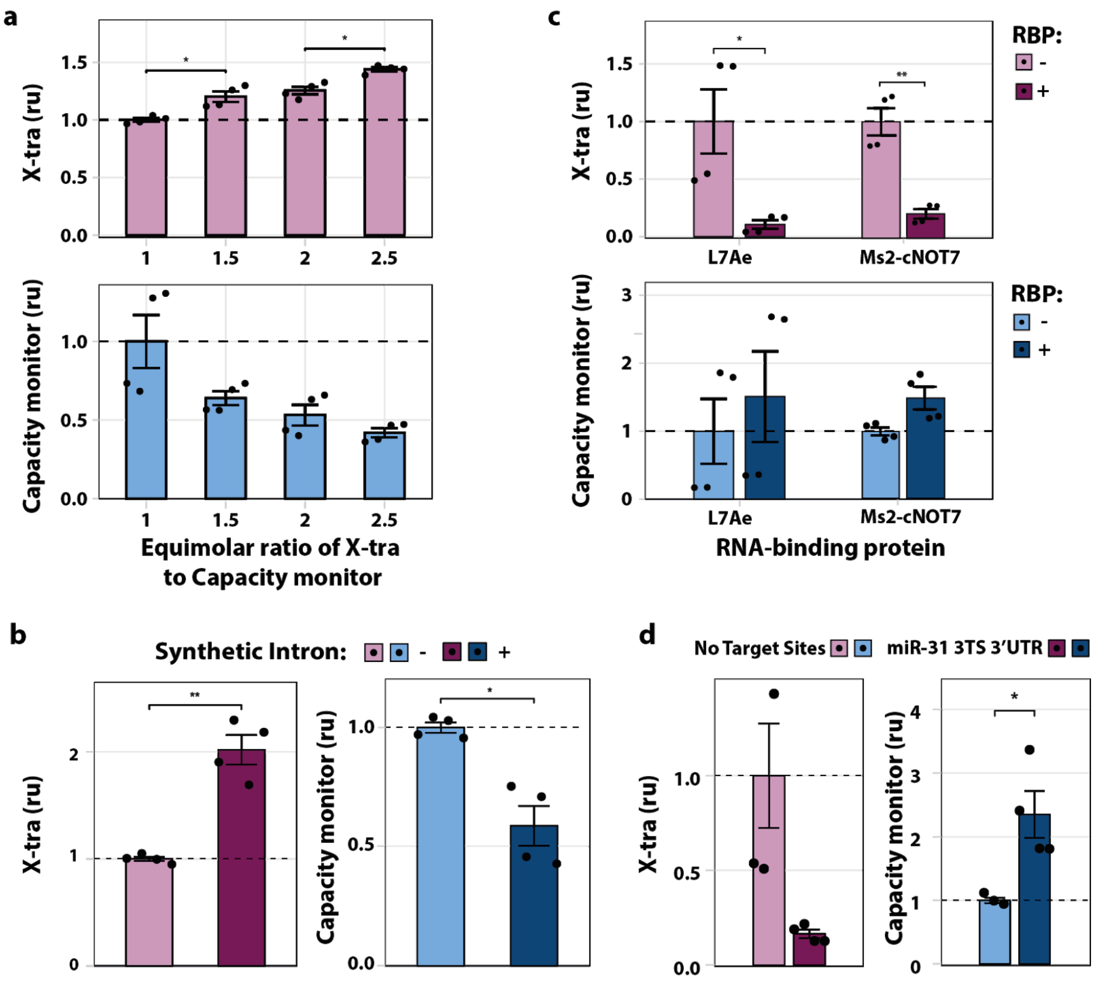

Supplementary Fig. 8. Gene expression burden in H1299 cells. (a) Flow cytometry results of H1299 cells co-transfected with fixed amount of capacity monitor and increasing amount of X-tra (1:1 to 1:2.5 molar ratio), both under CMV promoter regulation. Data show the mean fluorescence normalized to its value at a plasmid molar ratio of 1. N=4 biological replicates. Source data are provided as a Source Data file. **(b)** Flow cytometry results of H1299 cells co-transfected with X-tra (mKate) which includes or not a synthetic intron in the 5'UTR, and capacity monitor (EGFP). Data show that when mKate expression is enhanced by the synthetic intron, EGFP levels decrease. Data represent the mean fluorescence normalized to fluorescence values in the absence of the intron. N=4 biological replicates. Source data are provided as a Source Data file. **(c)** Flow cytometry results of H1299 cells co-transfected with 2kturn-EGFP or EGFP-8xMs2 (X-tra) and mKate (capacity monitor) in presence or absence of L7Ae or Ms2-cNOT7 respectively. Data show that when X-tra is down-regulated, the capacity monitor levels increase. Plot represents mean fluorescence normalization of fluorescence values to the condition without RBP. N=4 biological replicates. Source data are provided as a Source Data file. **(d)** Flow cytometry results of H1299 cells co-transfected with mKate (X-tra) that includes or not miR-31 target sites in the 5'UTR, and EGFP (capacity monitor). Data show that capacity monitor levels are higher when the X-tra is downregulated by miR-31. N=4 biological replicates (N = 3 for noTS). Source data are provided as a Source Data file. Plot represents normalization of mean fluorescence values to the no target site condition. Data were acquired 48 hours post-transfection. Error bars represent the standard error. ru: relative units. Unpaired two-sided T-test. p-value: **<0.005, *<0.05.

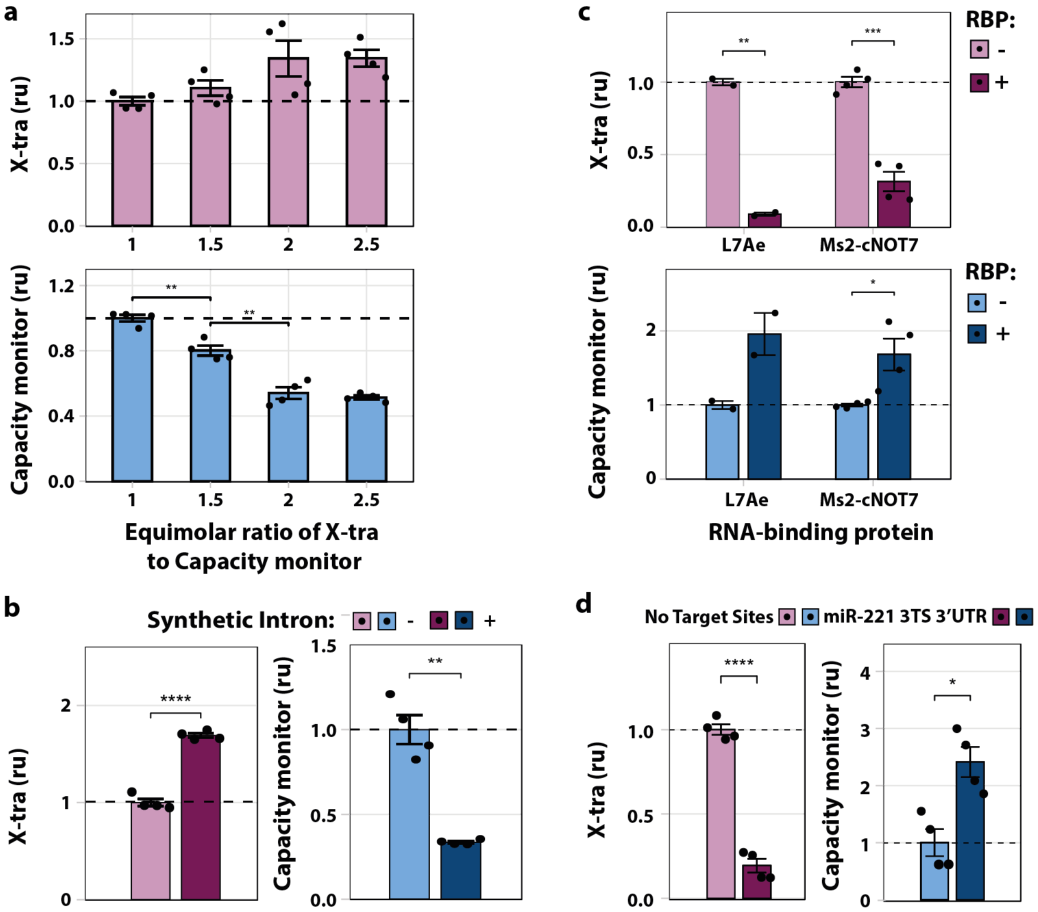

Supplementary Fig. 9. Gene expression burden in U2OS cells. (a) Flow cytometry results of U2OS cells co-transfected with fixed amount of capacity monitor and increasing amount of X-tra (1:1 to 1:2.5 molar ratio), both under CMV promoter regulation. Data show the mean fluorescence normalized to its value at a plasmid molar ratio of 1. N=4 biological replicates. Source data are provided as a Source Data file. **(b)** Flow cytometry results of U2OS cells co-transfected with X-tra (mKate) which includes or not a synthetic intron in the 5'UTR, and capacity monito*r* (EGFP). Data show that when mKate expression is enhanced by the synthetic intron, EGFP levels decrease. Data are the mean fluorescence normalized to fluorescence values in the absence of the intron. N=4 biological replicates. Source data are provided as a Source Data file. **(c)** Flow cytometry results of U2OS cells co-transfected with 2kturn-EGFP or EGFP-8xMs2 (X-tra) and mKate (capacity monitor) in presence or absence of L7Ae or Ms2-cNOT7 respectively. Data show that when X-tra is down-regulated, the capacity monitor levels increase. Plot represents normalization of mean fluorescence values to the condition without RBP. N=2 biological replicates for L7Ae and N=4 for Ms2-cNOT7. Source data are provided as a Source Data file. **(d)** Flow cytometry results of U2OS cells co-transfected with mKate (*X-tra*) that includes or not miR-221 target sites in the 5'UTR, and EGFP (capacity monitor). Data show that *capacity monitor* levels are higher when the X-tra is downregulated by miR-221. N=4 biological replicates. Source data are provided as a Source Data file. Plot represents normalization of mean fluorescence values to the no target site condition. Data were acquired 48 hours post-transfection. Error bars represent the standard error. ru: relative units. Unpaired two-sided T-test. p-value: **<0.005, *<0.05.

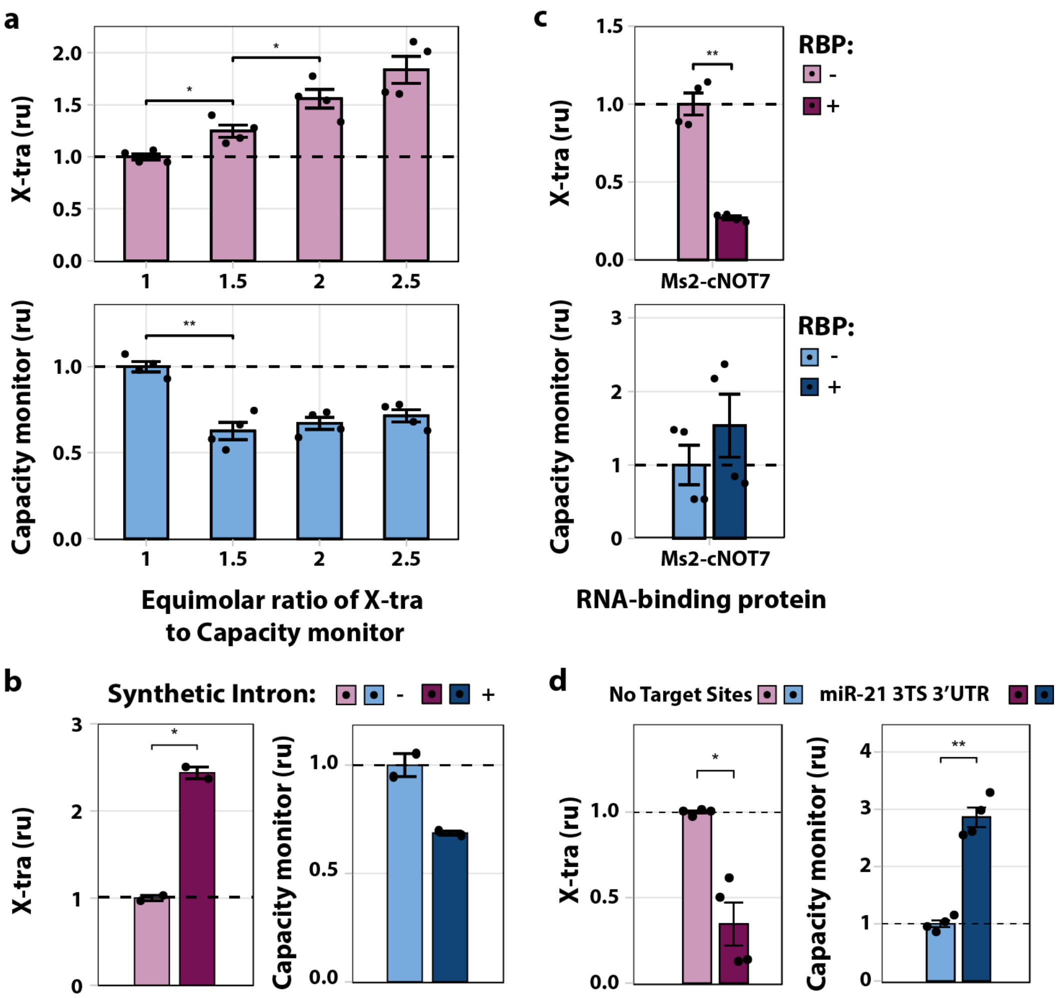

Supplementary Fig. 10. Gene expression burden in HeLa cells. (a) Flow cytometry results of HeLa cells co-transfected with fixed amount of capacity monitor and increasing amount of X-tra (1:1 to 1:2.5 molar ratio), both under CMV promoter regulation. Data show the mean fluorescence normalized to its value at a plasmid molar ratio of 1. N=4 biological replicates. Source data are provided as a Source Data file. **(b)** Flow cytometry results of HeLa cells co-transfected with X-tra (mKate) which includes or not a synthetic intron in the 5'UTR, and capacity monitor (EGFP). Data show that when mKate expression is enhanced by the synthetic intron, EGFP levels decrease. Data are the mean fluorescence normalized to fluorescence values in the absence of the intron. N=2 biological replicates. Source data are provided as a Source Data file. **(c)** Flow cytometry results of HeLa cells co-transfected with 2kturn-EGFP or EGFP-8xMs2 (X-tra) and mKate (capacity monitor) in presence or absence of L7Ae or Ms2-cNOT7 respectively. Data show that when X-tra is down-regulated, the capacity monitor levels increase. Plot represents normalization of mean fluorescence values to the condition without RBP. N=4 biological replicates. Source data are provided as a Source Data file. **(d)** Flow cytometry results of HeLa cells co-transfected with mKate (X-tra) that includes or not miR-21 target sites in the 5'UTR, and EGFP (capacity monitor). Data show that capacity monitor levels are higher when the X-tra is downregulated by miR-21. N=4 biological replicates. Source data are provided as a Source Data file. Plot represents normalization of mean fluorescence values to the no target site condition. Data were acquired 48 hours post-transfection. Error bars represent the standard error. ru: relative units. Unpaired two-sided T-test. p-value: **<0.005, *<0.05.

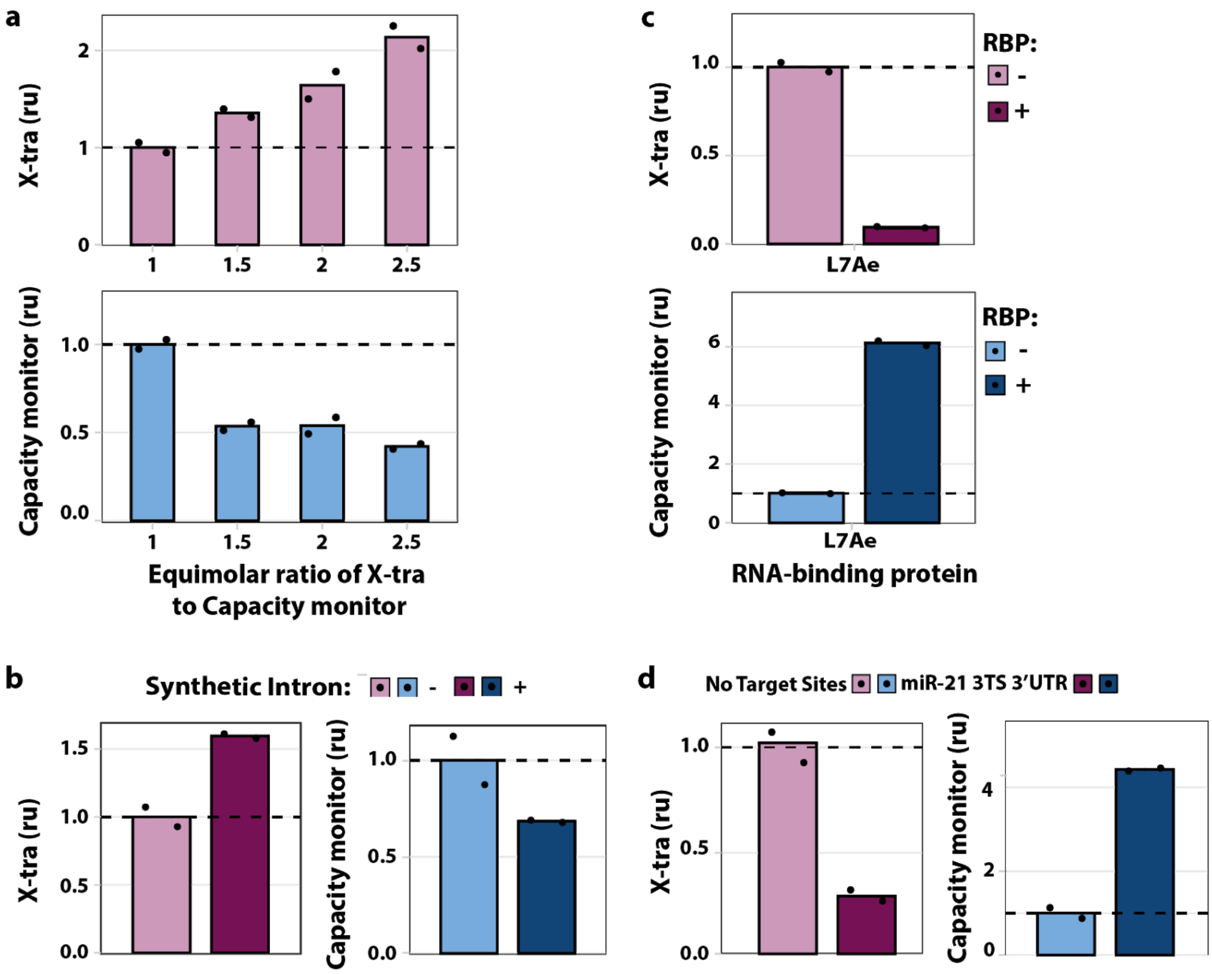

Supplementary Figure 11. Gene expression burden in CHO-K1 cells. (a) Flow cytometry results of CHO-K1 cells co-transfected with fixed amount of capacity monitor and increasing amount of X-tra (1:1 to 1:2.5 molar ratio), both under CMV promoter regulation. Data show the mean fluorescence normalized to its value at a plasmid molar ratio of 1. **(b)** Flow cytometry results of CHO-K1 cells co-transfected with X-tra (mKate) which includes or not a synthetic intron in the 5'UTR, and capacity monitor (EGFP). Data show that when mKate expression is enhanced by the synthetic intron, EGFP levels decrease. Data represent the mean fluorescence normalized to fluorescence values in the absence of the intron. **(c)** Flow cytometry results of CHO-K1 cells co-transfected with 2kturn-EGFP (X-tra) and mKate (capacity monitor) in presence or absence of L7Ae. Data show that when X-tra is down-regulated, the capacity monitor levels increase. Plot represents mean normalization of fluorescence values to the condition without L7Ae. **(d)** Flow cytometry results of CHO-K1 cells co-transfected with mKate (X-tra) that includes or not miR-21 target sites in the 5'UTR, and EGFP (capacity monitor). Data show that capacity monitor levels are higher when the X-tra is downregulated by miR-21. Plot represents normalization of mean fluorescence values to the no target site condition. Data were acquired 48 hours post-transfection. Error bars represent the standard error. ru: relative units. N=2 biological replicates. Source data are provided as a Source Data file.

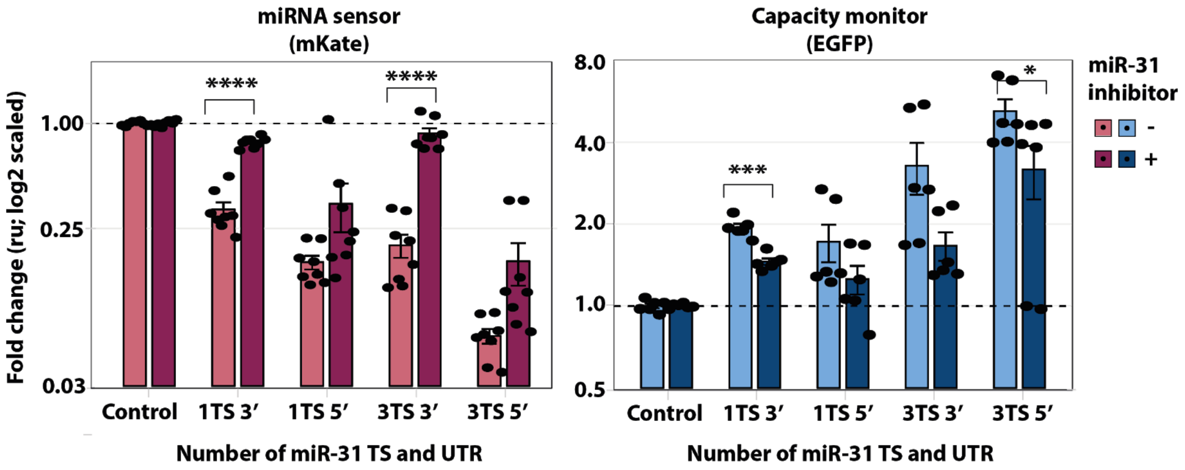

Supplementary Figure 12. Inhibition of miR-31 in H1299 cells. miR-31 activity impaired by a miR-31 inhibitor, leads to the rescue of miRNA sensor (mKate) expression in transfected H1299 cells. As a consequence, capacity monitor (EGFP) levels decrease. Both fluorescent proteins do not vary in the control. Data are expressed in logarithmic base 2 scale. Flow cytometry data were acquired 48 hours post-transfection and are plotted as mean fluorescence normalized on the control +/- SE. SE: standard error. ru: relative units. N=6 biological replicates. Source data are provided as a Source Data file. Unpaired two-sided T-test. p-value: ****<0.0001, ***<0.0005, *<0.05.

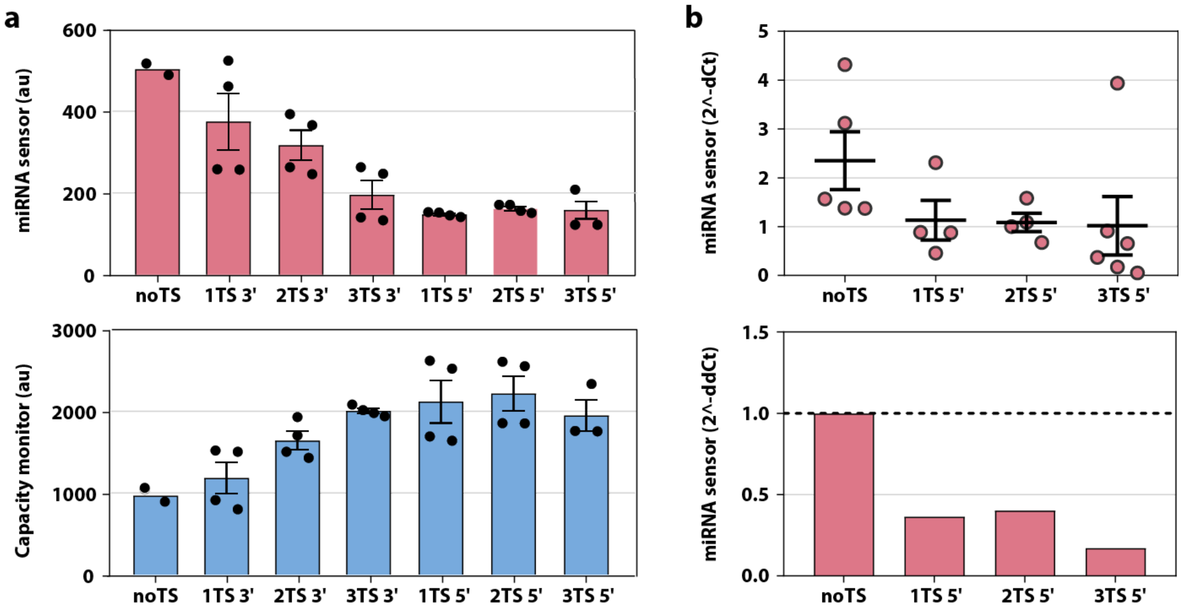

Supplementary Figure 13. miR-31 sensor in H1299. (a) Flow cytometry results of mKate-miR31-TS (miRNA sensor) co-transfected with EGFP (capacity monitor) in H1299 cells show that downregulation of miRNA sensor expression leads to an increase in capacity monitor levels. Data were acquired 48h post transfection and are plotted as mean fluorescence +/- SE. SE: standard error. au: arbitrary units. N=2 biological replicates (N=2 in noTS sample; N=3 in 3TS 5' sample). Source data are provided as a Source Data file. **(b)** qPCR measurement confirms lower mRNA levels of miRNA sensor. Top, scattered dot plot of 2^-dCt values. Data are plotted +/- SE. SE: standard error. Bottom, bar plot of the fold change measured with the 2^-ddCt method[8]. Data were acquired 48h post transfection. N=4 biological replicates. Source data are provided as a Source Data file.

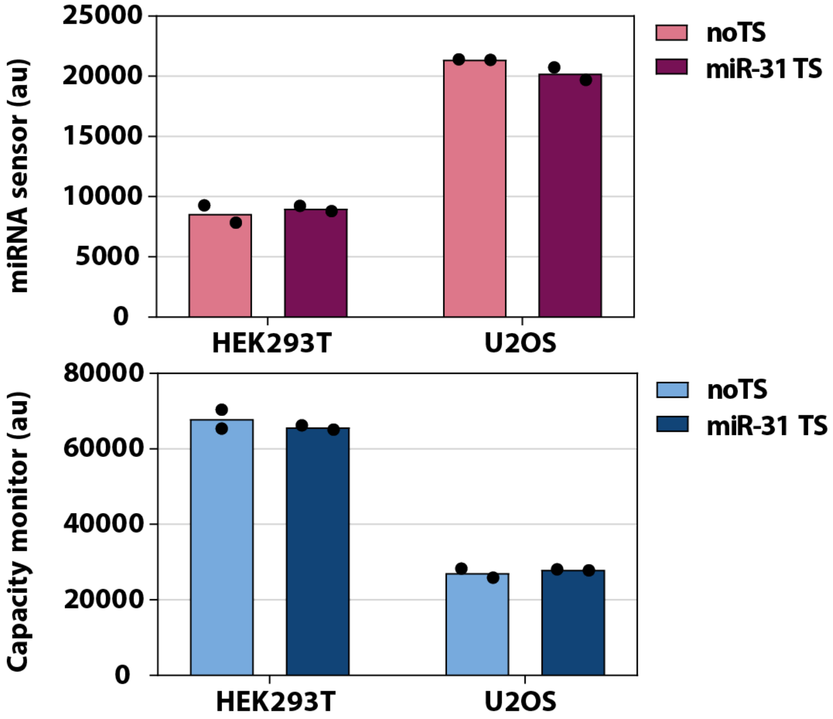

Supplementary Figure 14. The increase of capacity monitor levels is a consequence of miRNA regulation. U2OS and HEK293T cells were co-transfected with the 4-TS-3'UTR miR-31 sensor (miRNA sensor) and EGFP (capacity monitor). Both cell lines do not exhibit high expression of miR31, therefore miRNA sensor levels should not change. Data show that both miRNA sensor and the capacity monitor levels are comparable with and without miR-31 TS, indicating that the higher capacity monitor levels are indeed a consequence of miRNA activity. Data were acquired 48h post-transfection +/- SE. SE: standard error. au: arbitrary units. N=2 biological replicates. Source data are provided as a Source Data file.

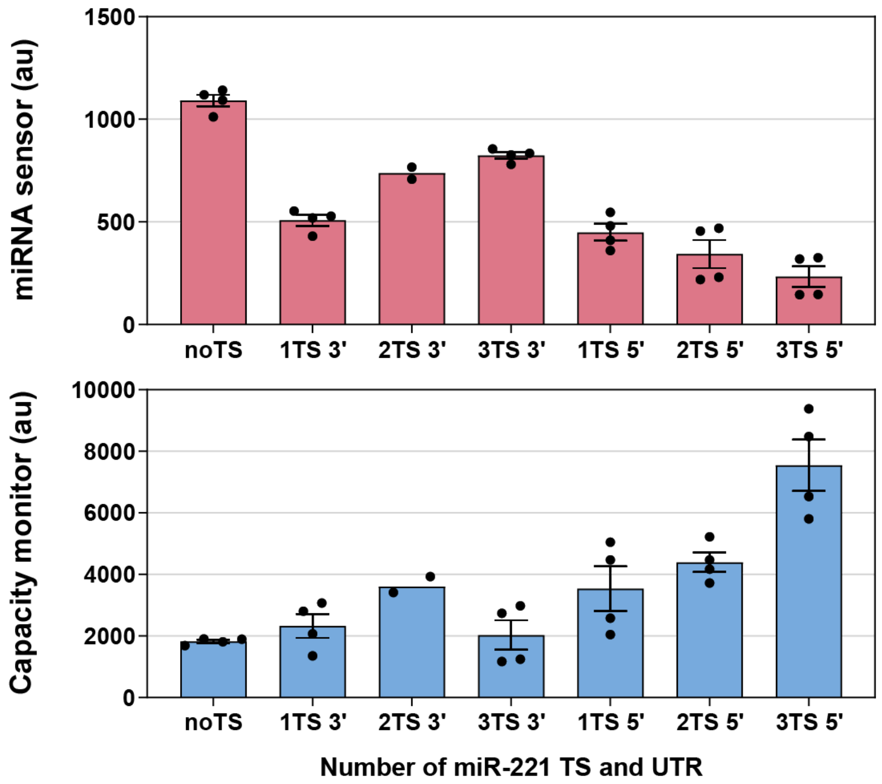

Supplementary Figure 15. miRNA-mediated resource re-allocation in the U2OS cell line. The miRNA sensor responds to miR-221, which is highly expressed in U2OS cells. Flow cytometry results of a co-transfection of mKate-miR221-TS (miRNA sensor) and EGFP (capacity monitor) in U2OS cells show the negative correlation of the two genes. Data were acquired 48h post-transfection and are plotted as mean fluorescence +/- SE. SE: standard error. au: arbitrary units. N=4 biological replicates (N=2 in 2TS 3' sample). Source data are provided as a Source Data file.

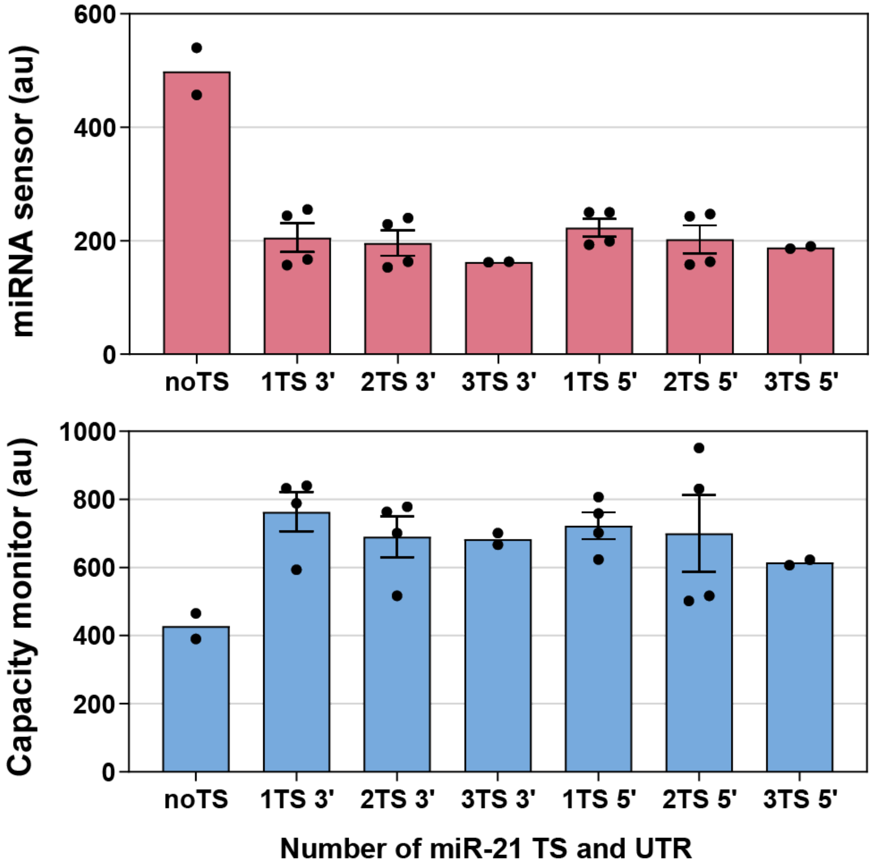

Supplementary Figure 16. miRNA-mediated resource re-allocation in the HeLa cell line. The miRNA sensor gene was designed for miR-21, which is highly expressed in HeLa cells. Flow cytometry results from a co-transfection of mKate-miR21-TS (miRNA sensor) and EGFP (capacity monitor) in HeLa cells. Interestingly, mKate downregulation seems to saturate already at 1TS3’. This may be due to the absolute levels of miR21 in this cell line. Data were acquired 48h post transfection and are plotted as mean fluorescence +/- SE. SE: standard error. au: arbitrary units. N=2 biological replicates for noTS, 3TS 3’, 3TS 5’ and N=4 for 1TS 3’, 2TS 3’, 1TS 5’ and 2TS 5’. Source data are provided as a Source Data file.

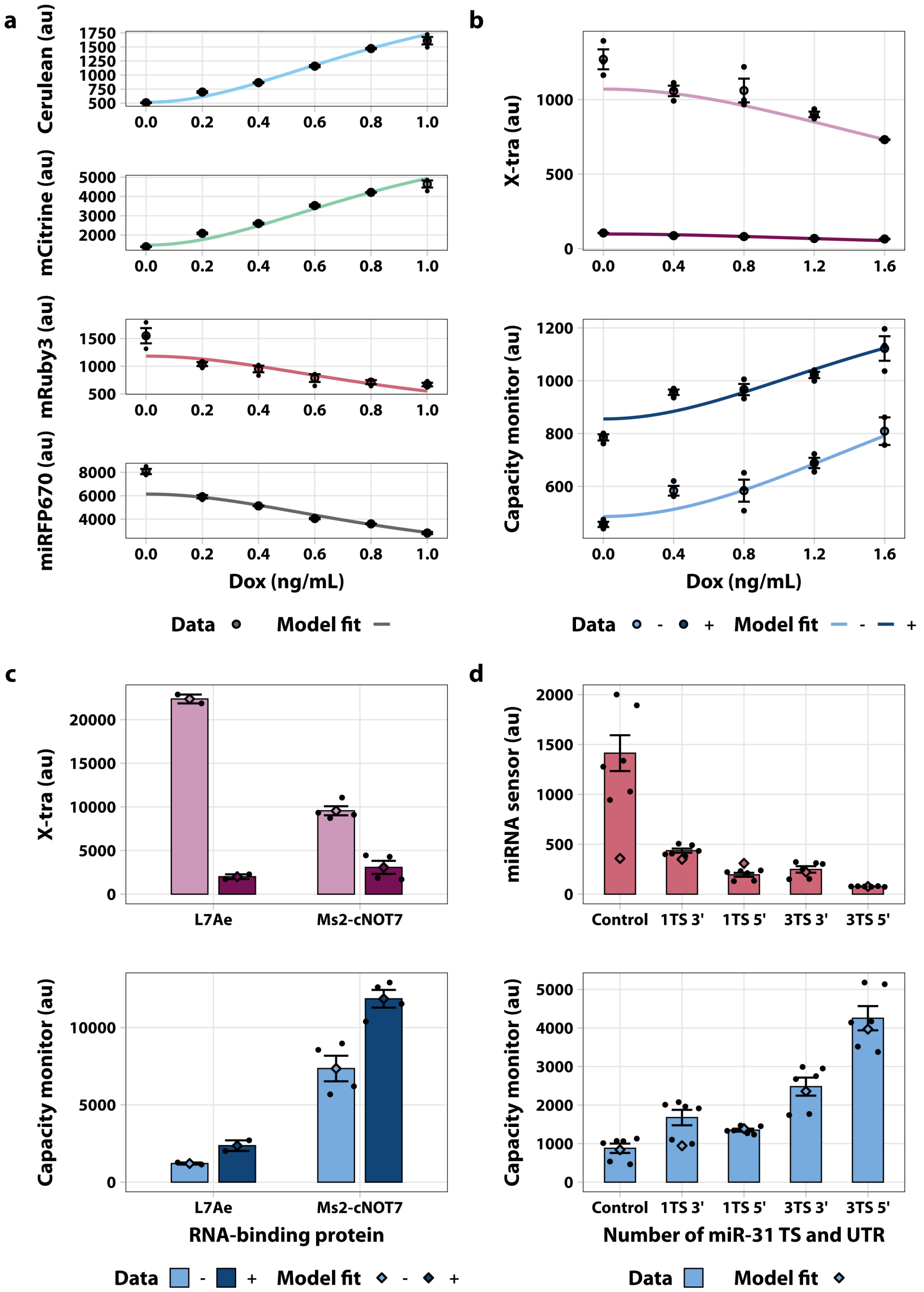

Supplementary Figure 17. Model fits for data displayed in Fig. 2b,e,g and Fig. 3b. The modeling framework described in **Fig. 4a** was applied to models of the genetic circuits used to generate the data in **Fig. 2b,e,g** and **Fig. 3b**. Detailed descriptions of these models can be found in **Supplementary Note 6**. The parameters obtained are summarized in **Supplementary Tables 13, 14, 15 and 16**. Source data for the respective figures are provided as a Source Data file. The data are presented as mean +/- SE. SE: standard error. au: arbitrary units.

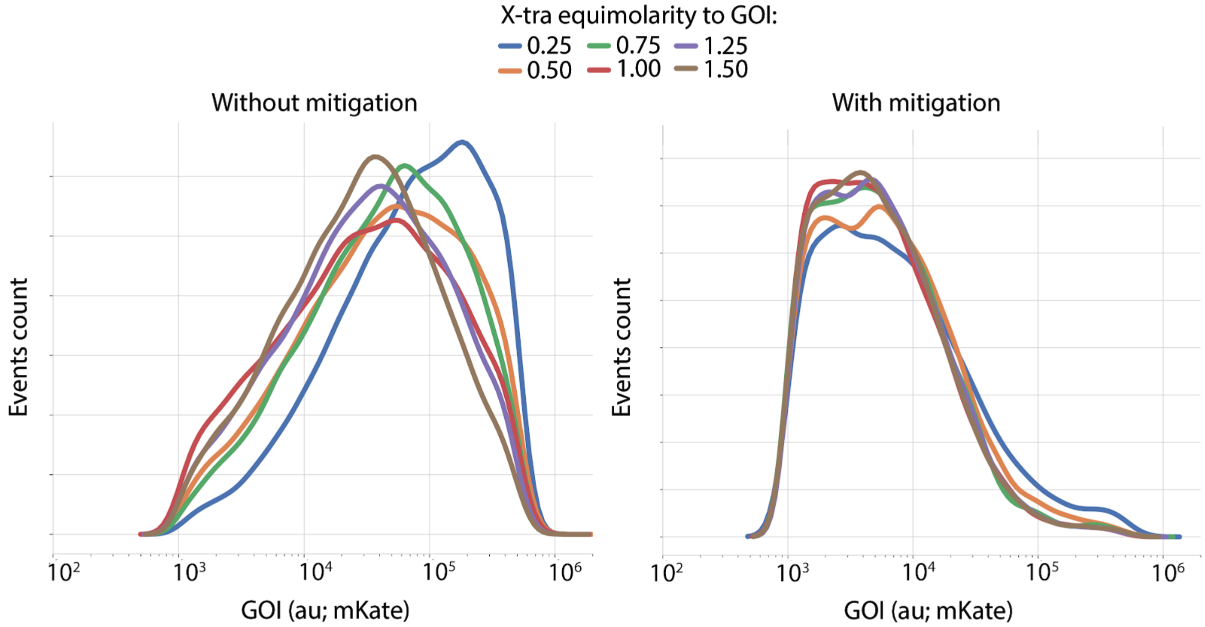

Supplementary Figure 18. GOI fluorescence distribution in co-transfected cells population. We compared the tolerance of mKate to increasing levels of X-tra gene in the absence or presence of an iFFL in which mKate includes miR-31 TS in the 5'UTR. The iFFL mitigation of resource competition is reflected by smaller shifts in GOI fluorescence at different equimolarities (right side). Data were acquired 48h post-transfection. Source data are provided as a Source Data file.

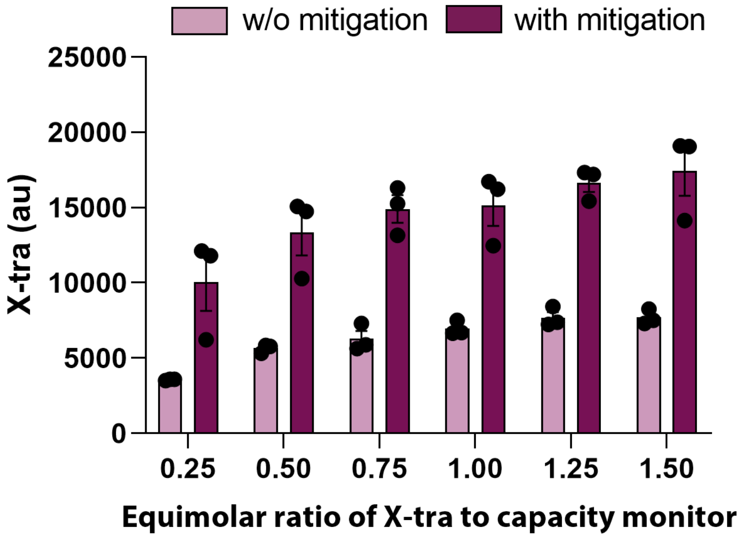

Supplementary Figure 19. X-tra absolute fluorescence in presence or absence of iFFL. X-tra expression levels of the plasmid titration experiment in **Fig. 5c**. With miR-31 iFFL mitigation, the absolute X-tra expression increases about 2 fold compared to w/o mitigation. Data is plotted as mean +/- SE. SE: standard error. au: arbitrary units. N=3 biological replicates. Source data are provided as a Source Data file.

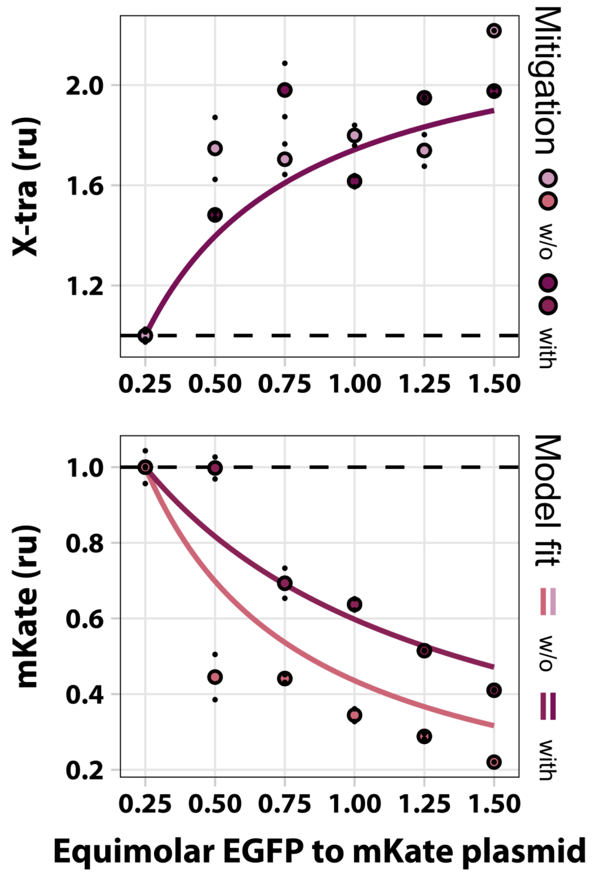

Supplementary Figure 20. The miR-221-based iFFL improves tolerance to exogenous gene load in U2OS cells. An iFFL whereby mKate includes miR-221 TS in the 5'UTR is less affected by the increased amount of the X-tra gene, as compared to the expression in the absence of miR-221 regulation. The model was unable to capture the differences in expression between the two conditions in the X-tra response due to the variability in the data. Therefore, the two lines plotted are exactly the same and it appears as if only one was plotted. Experimental data are normalized to the lowest equimolar ratio. The parameter values obtained by fitting are summarized in **Supplementary Table 10**. Data were acquired 48h post-transfection and are plotted +/- SE. SE: standard error. ru: relative units. N=2 biological replicates (N=1 for w/o Mitigation, 1.5 equimolar EGFP to mKate plasmid). Source data are provided as a Source Data file.

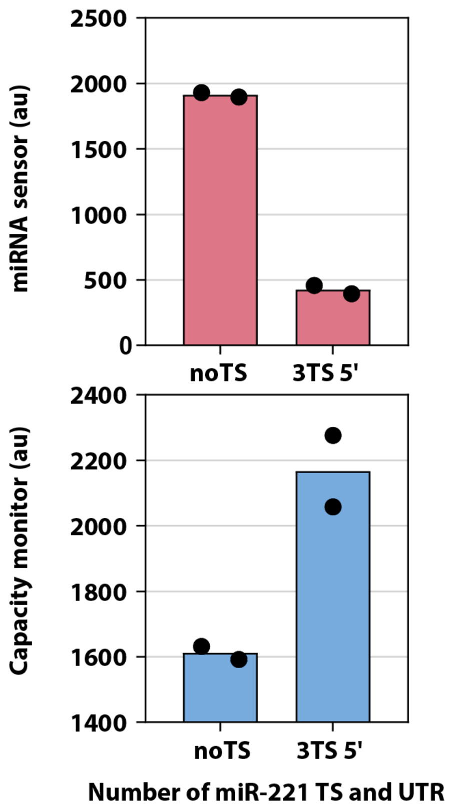

Supplementary Figure 21. miRNA-mediated resource re-allocation in the HEK293T cell line. The miRNA sensor gene was designed for miR-221, which is highly expressed in HEK293T cells. Flow cytometry results from a co-transfection of mKate-3xmiR221_5'UTR-TS (miRNA sensor) and EGFP (capacity monitor) in HEK293T cells. Data were acquired 48h post-transfection and are plotted +/- SE. SE: standard error. au: arbitrary units. N=2 biological replicates. Source data are provided as a Source Data file.

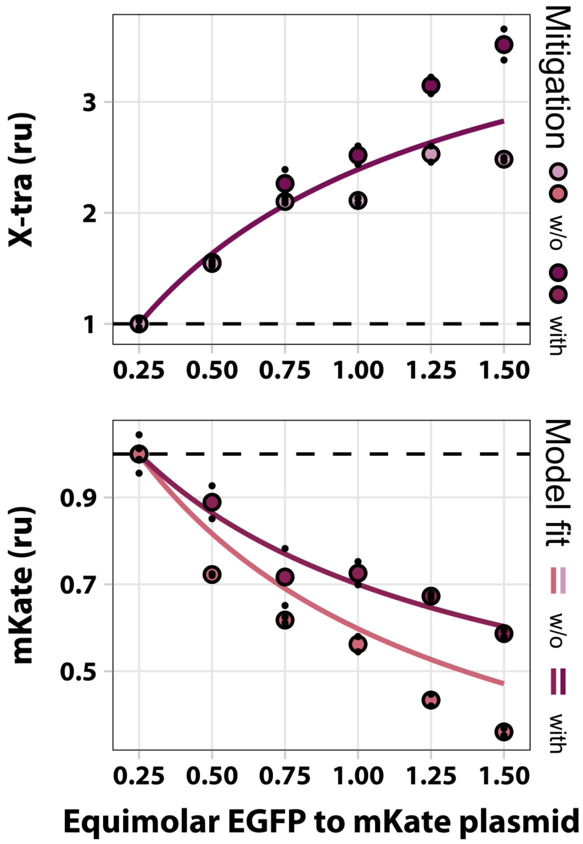

Supplementary Figure 22. The miR-221-based iFFL improves tolerance to exogenous gene load in HEK293T cells. We compared the tolerance of mKate to increasing levels of X-tra gene in the absence or presence of an iFFL whereby mKate includes miR-221 TS in the 5'UTR. The iFFL mitigates the effects of resource competition. The model was unable to capture the differences in expression between the two conditions in the X-tra response. Therefore, the two lines plotted are exactly the same and it appears as if only one was plotted. The parameter values obtained by fitting are summarized in **Supplementary Table 11**. Experimental data are normalized to the lowest equimolar ratio. Data were acquired 48h post-transfection and are plotted +/- SE. SE: standard error. ru: relative units. N=2 biological replicates. Source data are provided as a Source Data file.

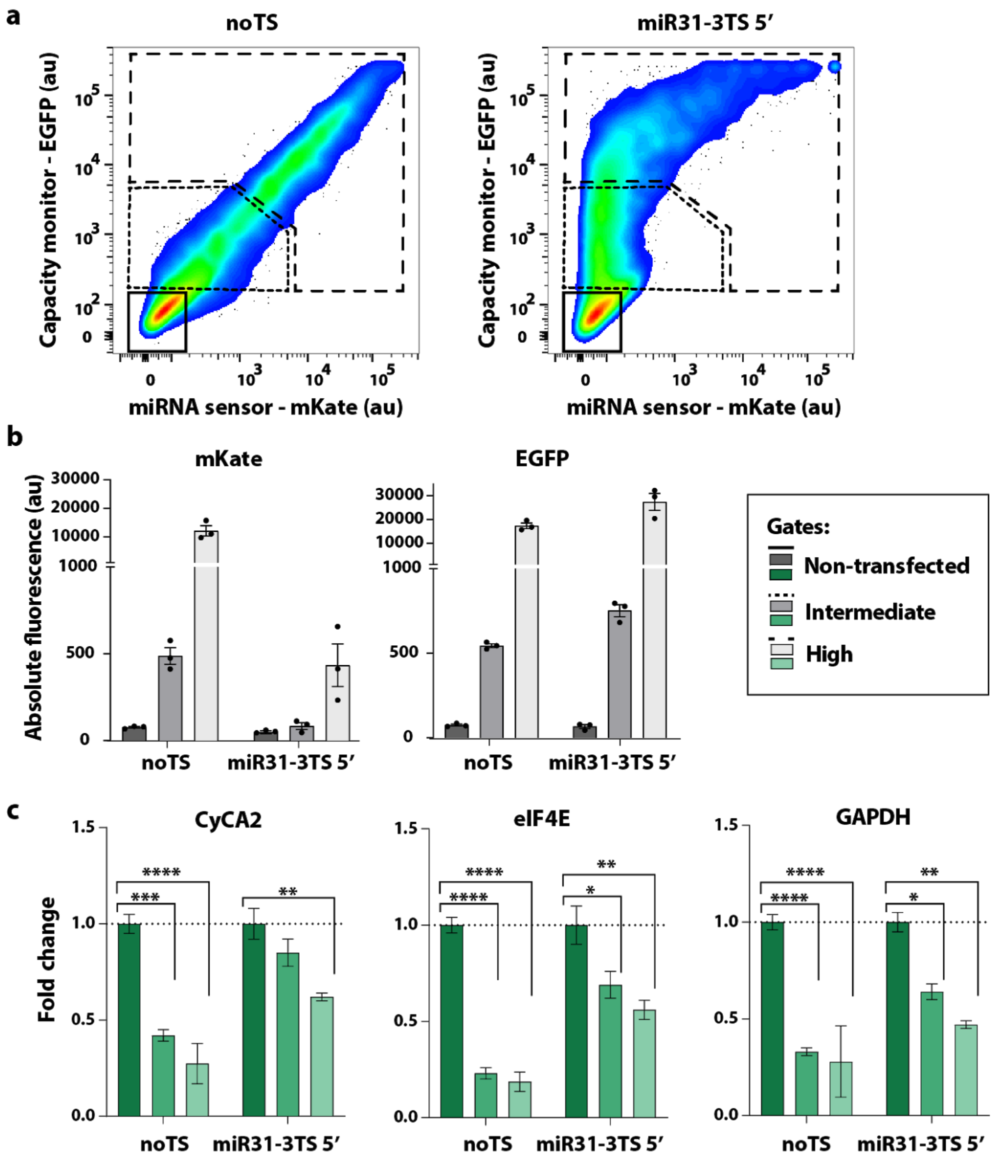

Supplementary Figure 23. Impact of transient plasmid transfection on endogenous genes in noTS and miR31-sensor samples. (a) H1299 cells were transfected with a bidirectional promoter plasmid encoding the fluorescent proteins EGFP (capacity monitor) and mKate (miRNA sensor), without **(noTS, left)** or with TS for miR-31 **(miR31-sensor, right)**. Cells were sorted by fluorescence intensity 48 hours after transfection to collect non-transfected, intermediate transfected and high transfected cells from the same transfection plate. **(b)** Protein levels of EGFP and mKate in sorted populations of noTS and miR31-sensor transfected cells. Consistent with the gates, fluorescence intensity increases in intermediate and high transfected cells when compared to non-transfected cells. In agreement with data shown in **Fig 2h and 3b,c**, EGFP fluorescence is higher in miR31-sensor samples, while mKate is lower. Data are the mean fluorescence +/- SE. **(c)** mRNA levels of CyCA2, eIF4E and GAPDH in sorted samples. All three endogenous genes decrease in intermediate and high transfected cells as compared to non-transfected cells. However, in cells transfected with the miR31-sensor circuit the decrease of expression is lower. mRNA levels are normalized to the non-transfected population. Data were collected 48 hours after transfection and are represented as mean +/- SE. SE: standard error. au: arbitrary units. Unpaired two-sided T-test. p-value: ****<0.0001, ***<0.0005, **<0.005, *<0.05. N=3 biological replicates. Source data are provided as a Source Data file.

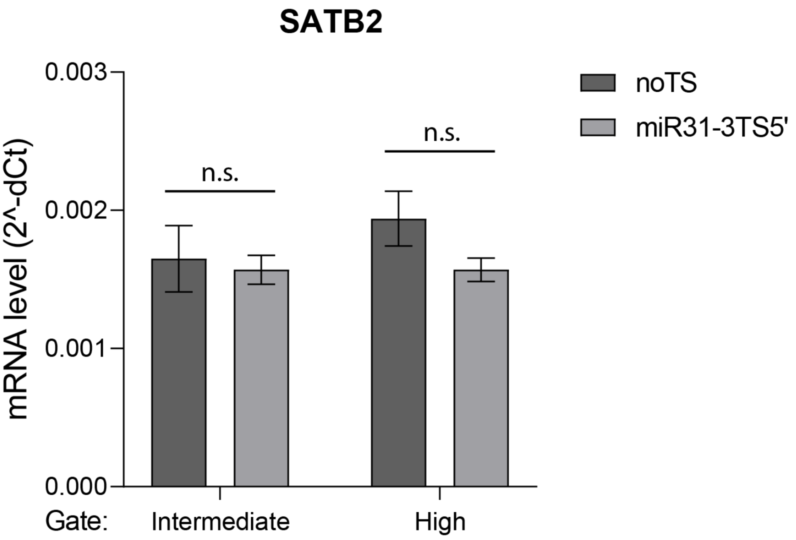

Supplementary Figure 24. Impact of noTS control vs miR-31-3TS5' transfections on the native miR-31 target SATB2. mRNA extracted from sorted populations as described in **Supplementary Fig. 22a** and analyzed in **Fig. 2d** and **Supplementary Fig. 22c**, was used to measure SATB2 levels relative to the internal reference 18S. SATB2 expression does not vary when miR-31 TS are present in our genetic circuit. Data represent the mean mRNA expression +/- SE. SE: standard error. ru: relative units. Unpaired two-sided T-test. N=3 biological replicates. Source data are provided as a Source Data file.

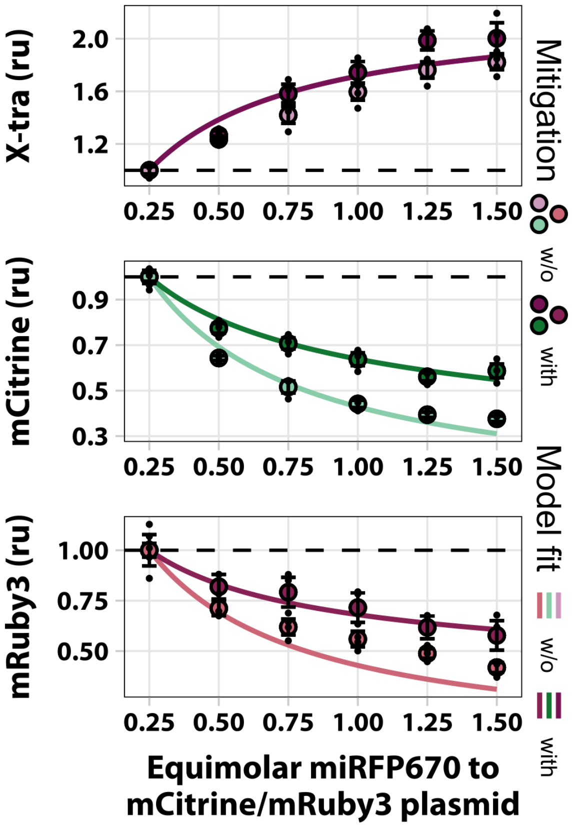

Supplementary Figure 25. The iFFL architecture improves tolerance to increase gene load in a 3-output system. Mouse embryonic stem cells were transfected with the miRNA mitigation iFFL shown in **Fig. 5d**. Light and dark colors represent gene expression levels in the absence or presence of mitigation. The solid lines show a model that includes resources, fit to the experimental data. Experimental data are normalized to the lowest equimolar ratio. The parameter values obtained by fitting are summarized in **Supplementary Table 12**. All data were acquired 48h post transfection and are plotted +/- SE. SE: standard error. ru: relative units. N=3 biological replicates. Source data are provided as a Source Data file.

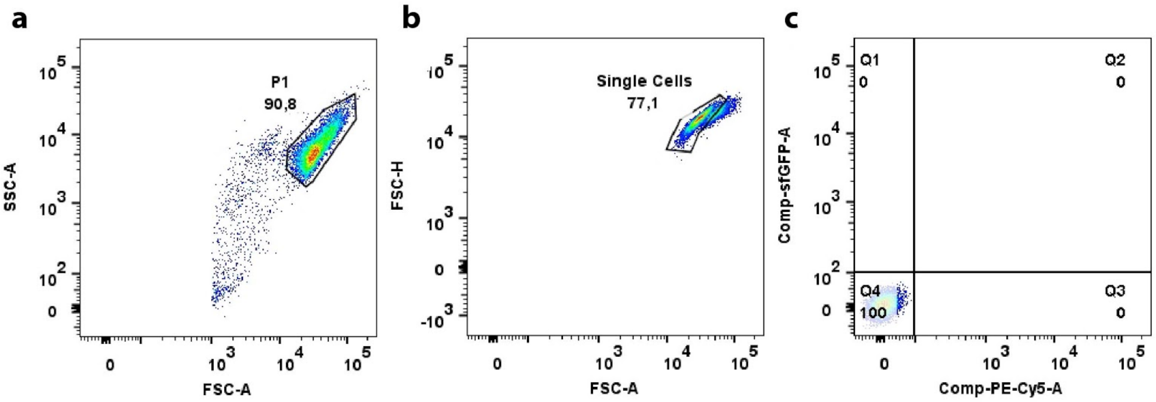

Supplementary Figure 26. FACS gating strategy. (a) The recorded events were gated in the FSC-A vs SSC-A channels to select the living cells population (P1). **(b)** The P1 was then gated in the FSC-A vs FSC-H channels to select the single cell population. **(c)** For each experiment a sample of non-transfected cells was used to set the positive threshold for each fluorescence. Cells selected following this pipeline were then analyzed.

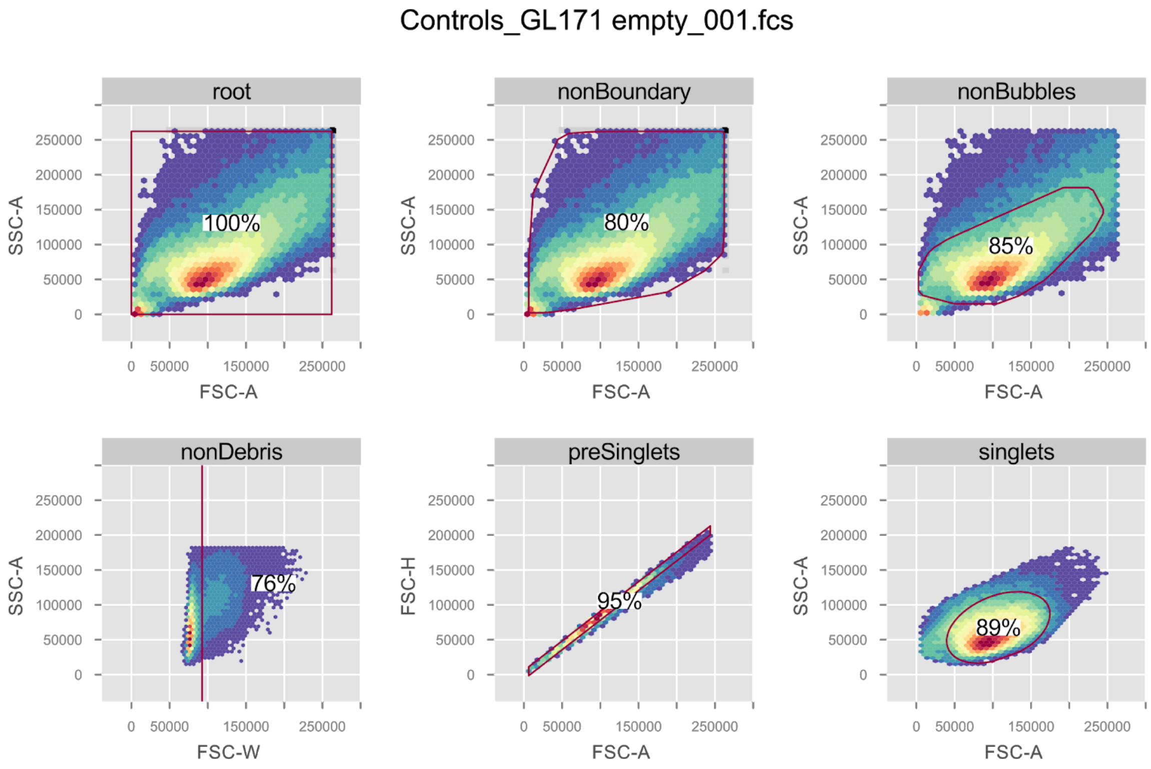

Supplementary Figure 27. Alternative FACS gating strategy. The plots show the hierarchical gating strategy implemented in a custom R script. The hierarchy progresses from left to right, top to bottom. **Top left:** The first gate removes events that potentially lie on the boundary of the detectable values. **Top middle:** This gate facilitates the subsequent gating by removing potential bubbles that were recorded. **Top right:** Here, a custom density-based gating strategy is employed to select for the living cell population and remove debris. **Bottom left:** In this gate the tail of the distribution in the FSC-W channel representing the bulk of the doublet event is removed. **Bottom middle:** The singlet population is further refined by gating in the FSC-H vs FSC-A channels. **Bottom right:** The resulting singlets are further refined by applying an ellipse gate around the point of highest density.

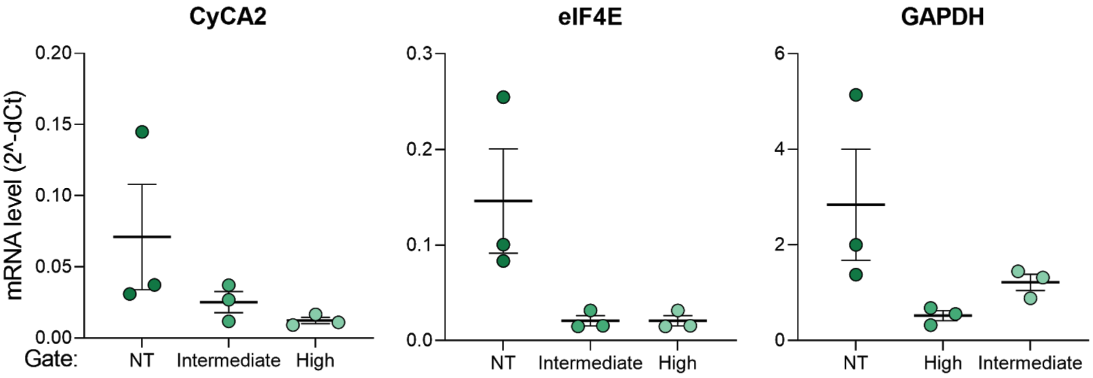

Supplementary Figure 28. mRNA level of data show in Fig. 2d. Individual measures of mRNA level in the 3 biological replicates. Source data are provided as a Source Data file. mRNA was extracted 48 hours post-transfection from sorted cells according to **Supplementary Fig. 5** gating. Data are plotted +/- SE. NT: Non Transfected. SE: standard error.

Supplementary Note 1. Derivation of the effective production rate constant $k_i^{\text{eff}}(A_1, \ldots, A_n)$.

Here we show the derivation of the effective production rate constant as the quasi-steady-state solution for the complexes formed between substrate species A_i and their corresponding resource pool R. The reactions shown in **Fig. 4a** are:

$$A_i + R \xrightarrow{k_i^+} C_i$$

$$C_i \xrightarrow{k_i^-} A_i + R$$

$$C_i \xrightarrow{k_i^{\text{cat}}} A_i + R + B_i$$

In these reactions, B_i is the product formed. We apply the law of mass action to get the following ordinary differential equation for the complex species C_i:

$$\dot{C}_i(t) = k_i^+ R(t) A_i(t) - \left(k_i^- + k_i^{\text{cat}}\right) C_i(t)$$

Assuming a limited amount of available resources, we can express the free resources as:

$$R(t) = R^{\text{total}} - \sum_{j=1}^{n} C_j(t)$$

Since we would like to obtain a quasi-steady-state expression for any n, we cast the equations in matrix from:

$$\dot{\mathbf{C}} = \text{diag}(\mathbf{k}^+)(R^{\text{total}} - \mathbf{1}^T\mathbf{C})\mathbf{A} - \text{diag}(\mathbf{k}^- + \mathbf{k}^{\text{cat}})\mathbf{C}$$

Here we use bold font to denote vectors and $\text{diag}(\mathbf{x})$ to represent the matrix with the vector $\mathbf{x}$ as its diagonal. When we set the left-hand side to zero, lump the binding, unbinding and production rates into $\mathbf{k_m}$ such that $k_{m_i} := \frac{k_i^- + k_i^{\text{cat}}}{k_i^+}$ and solve for $\mathbf{C}^*$ we get the expression:

$$\mathbf{C}^* = R^{\text{total}}(I + \text{diag}(\mathbf{k_m^{-1}})\mathbf{A}^*\mathbf{1}^{\text{T}})^{-1}\text{diag}(\mathbf{k_m^{-1}})\mathbf{A}^*$$

$$= R^{\text{total}}(I - \frac{\text{diag}(\mathbf{k_m^{-1}})\mathbf{A}^*\mathbf{1}^{\text{T}}}{1 + \mathbf{1}^{\text{T}}\text{diag}(\mathbf{k_m^{-1}})\mathbf{A}^*})\text{diag}(\mathbf{k_m})\mathbf{A}^* \quad \text{(Sherman-Morrison formula)}$$

$$= \frac{R^{\text{total}}\text{diag}(\mathbf{k_m^{-1}})\mathbf{A}^*}{1 + \mathbf{1}^{\text{T}}\text{diag}(\mathbf{k_m^{-1}})\mathbf{A}^*}$$

The effective production rate is obtained by multiplying this last expression with $\mathrm{diag}(\mathbf{k}^{\mathrm{cat}})$. For an individual element this gives:

$$k_i^{\mathrm{eff}}(A_1,\ldots,A_n) := k_i^{\mathrm{cat}} R^{\mathrm{total}} \frac{k_{m_i}^{-1} A_i}{1+\sum_{j=1}^{n} k_{m_j}^{-1} A_j}.$$

Supplementary Note 2. Simple model of plasmid copy number induced competition for limited resources.

To derive the equations used in Figure 1A of the main text the following model was used:

$$\dot{M}_i = k^{\text{eff}}_{M_i}(G_1,\dots,G_n)G_i - \delta_{M_i}M_i$$

$$\dot{P}_i = k^{\text{eff}}_{P_i}(M_1,\dots,M_n)M_i - \delta_{P_i}P_i$$

Here, we distinguish between two potential pools of shared limited resources. One for mRNA production and one for protein production. The mRNA species are denoted by the subscript letter M and the protein species are denoted by the subscript letter P. The copy number of the genes are denoted by G_i and are assumed to be constant. The degradation rates of each species is represented by a δ subscripted with the respective species' name. Using the definition:

$$k^{\text{eff}}_{B_i}(A_1,\dots,A_n) := k^{\text{cat}}_{B_i} R^{\text{total}} \frac{k^{-1}_{m_{B_i}}}{1+\sum_{j=1}^{n} k^{-1}_{m_{B_j}} A_j}$$

The equations can be written as:

$$\dot{M}_i = k^{\text{cat}}_{M_i} R^{\text{total}}_{M} \frac{k^{-1}_{m_{M_i}}}{1+\sum_{j=1}^{n} k^{-1}_{m_{M_j}} G_j} G_i - \delta_{M_i}M_i$$

$$\dot{P}_i = k^{\text{cat}}_{P_i} R^{\text{total}}_{P} \frac{k^{-1}_{m_{P_i}}}{1+\sum_{j=1}^{n} k^{-1}_{m_{P_j}} M_j} M_i - \delta_{P_i}P_i$$

We solve for the steady state expressions of each of the species by setting the left-hand side of the equations to zero. After simplifying we obtain the following expressions:

$$M_i^* = \frac{k^{\text{cat}}_{M_i} R^{\text{total}}_{M}}{\delta_{M_i}} \frac{k^{-1}_{m_{M_i}} G_i}{1+\sum_{j=1}^{n} k^{-1}_{m_{M_j}} G_j}$$

$$P_i^* = \frac{k^{\text{cat}}_{M_i} R^{\text{total}}_{M}}{\delta_{M_i}} \frac{k^{\text{cat}}_{P_i} R^{\text{total}}_{P}}{\delta_{P_i}} \frac{k^{-1}_{m_{M_i}} k^{-1}_{m_{P_i}} G_i}{1+\sum_{j=1}^{n} k^{-1}_{m_{M_j}} \left(1 + k^{-1}_{m_{P_j}} \frac{k^{\text{cat}}_{M_j} R^{\text{total}}_{M}}{\delta_{M_j}}\right) G_j}$$

The normalized expressions as shown in Figure 2A are further defined as $\hat{P}_i^* = \frac{P_i^*}{P_i^*|_{G_k = G_{k_0}}}$ with $k \neq i$, which gives:

$$\hat{P}_i^* = \frac{1 + k_{m_{M_k}}^{-1}\left(1 + k_{m_{P_k}}^{-1}\frac{k_{M_k}^{\mathrm{cat}} R_M^{\mathrm{total}}}{\delta_{M_k}}\right) G_{k_0} + \sum_{j\neq k}^{n} k_{m_{M_j}}^{-1}\left(1 + k_{m_{P_j}}^{-1}\frac{k_{M_j}^{\mathrm{cat}} R_M^{\mathrm{total}}}{\delta_{M_j}}\right) G_j}{1 + k_{m_{M_k}}^{-1}\left(1 + k_{m_{P_k}}^{-1}\frac{k_{M_k}^{\mathrm{cat}} R_M^{\mathrm{total}}}{\delta_{M_k}}\right) G_k + \sum_{j\neq k}^{n} k_{m_{M_j}}^{-1}\left(1 + k_{m_{P_j}}^{-1}\frac{k_{M_j}^{\mathrm{cat}} R_M^{\mathrm{total}}}{\delta_{M_j}}\right) G_j}$$

Specifically for Figure 2A n was set to 2.

Supplementary Note 3. Normalized gene expression of low absolute expression levels are more sensitive to reduced availability of resources.

To show that normalized expression is more sensitive to burden at low expression levels we take the general term for the protein levels derived in Supplementary Note 1.

$$P_i^* = \frac{k_{M_i}^{\text{cat}} R_M^{\text{total}}}{\delta_{M_i}} \frac{k_{P_i}^{\text{cat}} R_P^{\text{total}}}{\delta_{P_i}} \frac{k_{m_{M_i}}^{-1} k_{m_{P_i}}^{-1} G_i}{1 + \sum_{j=1}^{n} k_{m_{M_j}}^{-1} \left(1 + k_{m_{P_j}}^{-1} \frac{k_{M_j}^{\text{cat}} R_M^{\text{total}}}{\delta_{M_j}}\right) G_j}$$

To simplify the above term we lump parameters by setting:

$$\beta_{M_i} := k_{m_{P_i}}^{-1} \frac{k_{M_i}^{\text{cat}} R_M^{\text{total}}}{\delta_{M_i}}$$

$$\alpha_{P_i} := \frac{k_{P_i}^{\text{cat}} R_P^{\text{total}}}{\delta_{P_i}}$$

Which gives:

$$P_i^* = \frac{\alpha_{P_i} \beta_{M_i} k_{m_{M_i}}^{-1} G_i}{1 + \sum_{j=1}^{n} k_{m_{M_j}}^{-1} \left(1 + \beta_{M_j}\right) G_j}$$

From this term we can write the expression level normalized with respect to the expression level at G_{k0} as:

$$\hat{P}_i^* = \frac{P_i^*}{P_i^*|_{G_k = G_{k0}}} = \frac{1 + k_{m_{M_k}}^{-1} \left(1 + \beta_{M_k}\right) G_{k0} + \sum_{j \neq k}^{n} k_{m_{M_j}}^{-1} \left(1 + \beta_{M_j}\right) G_j}{1 + k_{m_{M_k}}^{-1} \left(1 + \beta_{M_k}\right) G_k + \sum_{j \neq k}^{n} k_{m_{M_j}}^{-1} \left(1 + \beta_{M_j}\right) G_j}$$

with $$\hat{P}_i^* = \frac{P_i^*}{P_i^*|_{G_k = G_{k0}}} = \frac{1 + k_{m_{M_k}}^{-1} \left(1 + \alpha_{M_k}\right) G_{k0} + \sum_{j \neq k}^{n} k_{m_{M_j}}^{-1} \left(1 + \alpha_{M_j}\right) G_j}{1 + k_{m_{M_k}}^{-1} \left(1 + \alpha_{M_k}\right) G_k + \sum_{j \neq k}^{n} k_{m_{M_j}}^{-1} \left(1 + \alpha_{M_j}\right) G_j}$$.

To test whether the normalized expression for a gene expressing at low levels is less sensitive to reduced resource availability than its high expressing counterpart we wish to evaluate the following inequality $\hat{P}_i^* < \hat{P}_i^*|_{G_i = \bar{G}_i}$ where the low expression is given by the assumption that $\bar{G}_i < G_i$. To simplify this inequality we write it as the equivalent inequality given by $\text{sgn}(\hat{P}_i^* - \hat{P}_i^*|_{G_i = \bar{G}_i}) < 0$ where sgn denotes the sign function. Plugging in the expression for $\hat{P}_i^*$ and $\hat{P}_i^*|_{G_i = \bar{G}_i}$ gives:

$$\operatorname{sgn}\left(\frac{1+k_{mM_k}^{-1}\left(1+\beta_{M_k}\right)G_{k_0}+\sum_{j\neq k}^{n}k_{mM_j}^{-1}\left(1+\beta_{M_j}\right)G_j}{1+k_{mM_k}^{-1}\left(1+\beta_{M_k}\right)G_k+\sum_{j\neq k}^{n}k_{mM_j}^{-1}\left(1+\beta_{M_j}\right)G_j}-\frac{1+k_{mM_i}^{-1}\left(1+\beta_{M_i}\right)\bar{G}_i+k_{mM_k}^{-1}\left(1+\beta_{M_k}\right)G_{k_0}+\sum_{j\neq\{i,k\}}^{n}k_{mM_j}^{-1}\left(1+\beta_{M_j}\right)G_j}{1+k_{mM_i}^{-1}\left(1+\beta_{M_i}\right)\bar{G}_i+k_{mM_k}^{-1}\left(1+\beta_{M_k}\right)G_k+\sum_{j\neq\{i,k\}}^{n}k_{mM_j}^{-1}\left(1+\beta_{M_j}\right)G_j}\right) < 0$$

Given that all parameters are positive and we demand that $G_k > G_{k_0}$ the term on the left hand side of the inequality above reduces to $\operatorname{sgn}(G_i - \bar{G}_i) < 0$. Given our initial assumption that $\bar{G}_i < G_i$ and the requirement that both are positive real numbers we find that the claim stated in the inequality is false because the sign function evaluates to 1. Therefore, the model shows that the normalized expression of a gene expressing at low absolute levels will be more affected by resource availability than its high expressing counterpart.

Supplementary Note 4. Model for the topologies from Lillacci et al. [1].

The model for the four topologies can be given by a system of ordinary differential equations, where setting the individual repression rates η_{M_1} and/or η_{M_2} of the microRNA to zero specifies the topology. More specifically, $\eta_{M_1} = 0$ and $\eta_{M_2} = 0$ is the open-loop (OLP) topology, $\eta_{M_1} = 0$ and $\eta_{M_2} > 0$ the incoherent feedforward (IFF) topology, $\eta_{M_1} > 0$ and $\eta_{M_2} = 0$ is the feedback (FBK) topology and $\eta_{M_1} > 0$ and $\eta_{M_2} > 0$ is the hybrid (HYB) topology.

$$\dot{M}_1 = k_{M_1}^{\text{eff}}(G_1, G_2 f(P_1), G_3)G_1 - (\delta_{M_1} + \eta_{M_1} m)\, M_1$$
$$\dot{M}_2 = k_{M_2}^{\text{eff}}(G_1, G_2 f(P_1), G_3)G_2 f(P_1) - (\delta_{M_2} + \eta_{M_2} m)\, M_2$$
$$\dot{M}_3 = k_{M_3}^{\text{eff}}(G_1, G_2 f(P_1), G_3)G_3 - \delta_{M_3} M_3$$
$$\dot{m} = k_{m}^{\text{eff}}(G_1, G_2 f(P_1), G_3)G_2 f(P_1) - \delta_m m$$
$$\dot{P}_1 = k_{P_1}^{\text{eff}}(M_1, M_2, M_3)M_1 - \delta_{P_1} P_1$$
$$\dot{P}_2 = k_{P_2}^{\text{eff}}(M_1, M_2, M_3)M_2 - \delta_{P_2} P_2$$
$$\dot{P}_1 = k_{P_3}^{\text{eff}}(M_1, M_2, M_3)M_3 - \delta_{P_3} P_3$$

In this system of equations the species M_1, M_2 and M_3 correspond to tTA-Cer. mRNA, DsRed mRNA and mCitr. mRNA respectively as shown in Figure 4B and C. P_1 denotes the transcriptional activator tTA-Cer., P_2 denotes the fluorescent protein DsRed and P_3 denotes the fluorescent protein mCitrine. m denotes miR-FF4 expressed from the same gene as DsRed. Furthermore, G_1, G_2 and G_3 correspond to the plasmid copy number of tTA-Cer., DsRed and mCitr. Respectively. The rates beginning with a δ denote the degradation rates of the species written in the subscript. The rates beginning with η correspond to the repression rates of the microRNA FF4. Lastly, the transcriptional activation was modeled by a hill-type function $f(x) := \frac{x^h}{\kappa^h + x^h}$. The steady states for the protein species used for fitting can be written as:

$$P_1^* = \frac{\alpha_{P_1^*} \frac{\beta_{M_1} G_1 k_{m_{M_1}}^{-1}}{1 + G_1 k_{m_{M_1}}^{-1} + \gamma_{M_2} f(P_1^*)\left(1 + \theta_{M_1}\right) + \gamma_{M_3}}}{1 + \frac{\beta_{M_1} G_1 k_{m_{M_1}}^{-1}}{1 + G_1 k_{m_{M_1}}^{-1} + \gamma_{M_2} f(P_1^*)\left(1 + \theta_{M_1}\right) + \gamma_{M_3}} + \frac{\beta_{M_2} \gamma_{M_2} f(P_1^*)}{1 + G_1 k_{m_{M_1}}^{-1} + \gamma_{M_2} f(P_1^*)\left(1 + \theta_{M_2}\right) + \gamma_{M_3}} + \frac{\beta_{M_3} \gamma_{M_3}}{1 + G_1 k_{m_{M_1}}^{-1} + \gamma_{M_2} f(P_1^*) + \gamma_{M_3}}}$$

$$P_2^* = \frac{\alpha_{P_2} \frac{\beta_{M_2} \gamma_{M_2} f(P_1^*)}{1 + G_1 k_{m_{M_1}}^{-1} + \gamma_{M_2} f(P_1^*)\left(1 + \theta_{M_2}\right) + \gamma_{M_3}}}{1 + \frac{\beta_{M_1} G_1 k_{m_{M_1}}^{-1}}{1 + G_1 k_{m_{M_1}}^{-1} + \gamma_{M_2} f(P_1^*)\left(1 + \theta_{M_1}\right) + \gamma_{M_3}} + \frac{\beta_{M_2} \gamma_{M_2} f(P_1^*)}{1 + G_1 k_{m_{M_1}}^{-1} + \gamma_{M_2} f(P_1^*)\left(1 + \theta_{M_2}\right) + \gamma_{M_3}} + \frac{\beta_{M_3} \gamma_{M_3}}{1 + G_1 k_{m_{M_1}}^{-1} + \gamma_{M_2} f(P_1^*) + \gamma_{M_3}}}$$

$$P_3^* = \frac{\alpha_{P_3} \frac{\beta_{M_3}\gamma_{M_3}}{1+G_1 k_{m_{M_1}}^{-1} + \gamma_{M_2} f(P_1^*) + \gamma_{M_3}}}{1 + \frac{\beta_{M_1} G_1 k_{m_{M_1}}^{-1}}{1+G_1 k_{m_{M_1}}^{-1} + \gamma_{M_2} f(P_1^*)\left(1+\theta_{M_1}\right) + \gamma_{M_3}} + \frac{\beta_{M_2}\gamma_{M_2} f(P_1^*)}{1+G_1 k_{m_{M_1}}^{-1} + \gamma_{M_2} f(P_1^*)\left(1+\theta_{M_2}\right) + \gamma_{M_3}} + \frac{\beta_{M_3}\gamma_{M_3}}{1+G_1 k_{m_{M_1}}^{-1} + \gamma_{M_2} f(P_1^*) + \gamma_{M_3}}}$$

Here, α_{P_i} and β_{M_i} are the same as defined in Supplementary Note 2. Additionally, $\gamma_{M_i} := k_{m_{M_i}}^{-1} G_i$ for $i \in \{2, 3\}$ and $\theta_{M_i} := \frac{\eta_{M_i}}{\delta_{M_i}} \frac{k_{M_2}^{\text{cat}} R_M^{\text{total}}}{\delta_m}$ were introduced. The equations were fit in the implicit form shown because a closed form solution could not be obtained.

Supplementary Note 5. Models for endogenous microRNA-based iFFL and synthetic microRNA-based iFFL circuits.

Endogenous microRNA-based iFFL:

The system of equations used to derive the steady state expressions is given by:

$$\dot{M}_1 = k^{\text{eff}}_{M_1}(G_1, G_2, G_m)G_1 - \delta_{M_1} M_1$$

$$\dot{M}_2 = k^{\text{eff}}_{M_2}(G_1, G_2, G_m)G_2 - \left(\delta_{M_2} + \eta_{M_2} m\right) M_2$$

$$\dot{m} = k^{\text{eff}}_{m}(G_1, G_2, G_m)G_m - \delta_m m$$

$$\dot{P}_1 = k^{\text{eff}}_{P_1}(M_1, M_2)M_1 - \delta_{P_1} P_1$$

$$\dot{P}_2 = k^{\text{eff}}_{P_2}(M_1, M_2)M_2 - \delta_{P_2} P_2$$

Here, M_1 and M_2 represent the mRNA species for the fluorescent proteins EGFP (*X-tra*) and mKate (*GOI*) respectively. P_1 and P_2 correspond to the proteins themselves and m denotes the microRNA miR-31. As in Supplementary Note 1 and 3, the degradation rates are shown as δ subscripted with the species they correspond to and the repression rates are shown as η subscripted with the respective species. G_1 and G_2 are the plasmid copy number of the EGFP and mKate plasmids respectively and G_m is the copy number of the microRNA on the genome. The steady states for the protein species can be written as:

$$P_1^* = \frac{\alpha_{P_1} \frac{\beta_{M_1} G_1 k^{-1}_{m_{M_1}}}{1+G_1 k^{-1}_{m_{M_1}}+\gamma_{M_2}+\gamma_m}}{1 + \frac{\beta_{M_1} G_1 k^{-1}_{m_{M_1}}}{1+G_1 k^{-1}_{m_{M_1}}+\gamma_{M_2}+\gamma_m} + \frac{\beta_{M_2}\gamma_{M_2}}{1+G_1 k^{-1}_{m_{M_1}}+\gamma_{M_2}+\gamma_m\left(1+\theta_{M_2}\right)}}$$

$$P_2^* = \frac{\alpha_{P_2} \frac{\beta_{M_2}\gamma_{M_2}}{1+G_1 k^{-1}_{m_{M_1}}+\gamma_{M_2}+\gamma_m\left(1+\theta_{M_2}\right)}}{1 + \frac{\beta_{M_1} G_1 k^{-1}_{m_{M_1}}}{1+G_1 k^{-1}_{m_{M_1}}+\gamma_{M_2}+\gamma_m} + \frac{\beta_{M_2}\gamma_{M_2}}{1+G_1 k^{-1}_{m_{M_1}}+\gamma_{M_2}+\gamma_m\left(1+\theta_{M_2}\right)}}$$

For fitting, the expressions were normalized the same way as introduced in Supplementary Note 1 and 2 which yields the expressions:

$$\hat{P}_1^* = \frac{G_1\left(1+G_{1_0} k^{-1}_{m_{M_1}}+\gamma_{M_2}+\gamma_m\right)}{G_{1_0}\left(1+G_1 k^{-1}_{m_{M_1}}+\gamma_{M_2}+\gamma_m\right)} \frac{1 + \frac{\beta_{M_1} G_{1_0} k^{-1}_{m_{M_1}}}{1+G_{1_0} k^{-1}_{m_{M_1}}+\gamma_{M_2}+\gamma_m} + \frac{\beta_{M_2}\gamma_{M_2}}{1+G_{1_0} k^{-1}_{m_{M_1}}+\gamma_{M_2}+\gamma_m\left(1+\theta_{M_2}\right)}}{1 + \frac{\beta_{M_1} G_1 k^{-1}_{m_{M_1}}}{1+G_1 k^{-1}_{m_{M_1}}+\gamma_{M_2}+\gamma_m} + \frac{\beta_{M_2}\gamma_{M_2}}{1+G_1 k^{-1}_{m_{M_1}}+\gamma_{M_2}+\gamma_m\left(1+\theta_{M_2}\right)}}$$

$$\hat{P}_2^* = \frac{1 + G_{1_0} k_{m_{M_1}}^{-1} + \gamma_{M_2} + \gamma_m \left(1 + \theta_{M_2}\right)}{1 + G_1 k_{m_{M_1}}^{-1} + \gamma_{M_2} + \gamma_m \left(1 + \theta_{M_2}\right)} \frac{1 + \frac{\beta_{M_1} G_{1_0} k_{m_{M_1}}^{-1}}{1+G_{1_0} k_{m_{M_1}}^{-1} + \gamma_{M_2} + \gamma_m} + \frac{\beta_{M_2} \gamma_{M_2}}{1+G_{1_0} k_{m_{M_1}}^{-1} + \gamma_{M_2} + \gamma_m \left(1+\theta_{M_2}\right)}}{1 + \frac{\beta_{M_1} G_1 k_{m_{M_1}}^{-1}}{1+G_1 k_{m_{M_1}}^{-1} + \gamma_{M_2} + \gamma_m} + \frac{\beta_{M_2} \gamma_{M_2}}{1+G_1 k_{m_{M_1}}^{-1} + \gamma_{M_2} + \gamma_m \left(1+\theta_{M_2}\right)}}$$

Synthetic microRNA-based iFFL:

The system of equations used to obtain the steady state expressions is given by:

$$\dot{M}_1 = k_{M_1}^{\text{eff}}(G_1, G_2) G_1 - \delta_{M_1} M_1$$

$$\dot{M}_2 = k_{M_2}^{\text{eff}}(G_1, G_2) G_2 - \left(\delta_{M_2} + \eta_{M_2} m\right) M_2$$

$$\dot{M}_3 = k_{M_3}^{\text{eff}}(G_1, G_2) G_2 - \left(\delta_{M_3} + \eta_{M_3} m\right) M_3$$

$$\dot{m} = k_m^{\text{eff}}(G_1, G_2) G_2 - \delta_m m$$

$$\dot{P}_1 = k_{P_1}^{\text{eff}}(M_1, M_2, M_3) M_1 - \delta_{P_1} P_1$$

$$\dot{P}_2 = k_{P_2}^{\text{eff}}(M_1, M_2, M_3) M_2 - \delta_{P_2} P_2$$

$$\dot{P}_1 = k_{P_3}^{\text{eff}}(M_1, M_2, M_3) M_3 - \delta_{P_3} P_3$$

Here, M_1, M_2 and M_3 denote the mRNA species of the fluorescent proteins miRFP670 (*X-tra*), mCitrine (*GOI*$_1$) and mRuby3 (*GOI*$_2$) respectively. Similarly, P_1, P_2 and P_3 denote their protein species and m represents the microRNA FF4. G_1 corresponds to the plasmid which encodes miRFP670 and G_2 corresponds to the plasmid which encodes both the transcriptional unit of mCitrine and the transcriptional unit of mRuby3 and the microRNA. Again, rates beginning with δ describe degradation rates, while rates beginning with η denote the repression by the microRNA. Compared to the endogenous system, the microRNA FF4 is produced from the same gene as mRuby3 and therefore we model their production rates in a similar manner. The steady states for the protein species can be obtained to be:

$$P_1^* = \frac{\alpha_{P_1} \frac{\beta_{M_1} G_1 k_{m_{M_1}}^{-1}}{1+G_1 k_{m_{M_1}}^{-1} + \gamma_{M_2} + \gamma_{M_3}}}{1 + \frac{\beta_{M_1} G_1 k_{m_{M_1}}^{-1}}{1+G_1 k_{m_{M_1}}^{-1} + \gamma_{M_2} + \gamma_{M_3}} + \frac{\beta_{M_2} \gamma_{M_2}}{1+G_1 k_{m_{M_1}}^{-1} + \gamma_{M_2} + \gamma_{M_3}\left(1+\theta_{M_2}\right)} + \frac{\beta_{M_3} \gamma_{M_3}}{1+G_1 k_{m_{M_1}}^{-1} + \gamma_{M_2} + \gamma_{M_3}\left(1+\theta_{M_3}\right)}}$$

$$P_2^* = \frac{\alpha_{P_2} \frac{\beta_{M_2} \gamma_{M_2}}{1+G_1 k_{m_{M_1}}^{-1} + \gamma_{M_2} + \gamma_{M_3}\left(1+\theta_{M_2}\right)}}{1 + \frac{\beta_{M_1} G_1 k_{m_{M_1}}^{-1}}{1+G_1 k_{m_{M_1}}^{-1} + \gamma_{M_2} + \gamma_{M_3}} + \frac{\beta_{M_2} \gamma_{M_2}}{1+G_1 k_{m_{M_1}}^{-1} + \gamma_{M_2} + \gamma_{M_3}\left(1+\theta_{M_2}\right)} + \frac{\beta_{M_3} \gamma_{M_3}}{1+G_1 k_{m_{M_1}}^{-1} + \gamma_{M_2} + \gamma_{M_3}\left(1+\theta_{M_3}\right)}}$$

$$P_3^* = \frac{\alpha_{P_3} \frac{\beta_{M_3}\gamma_{M_3}}{1+G_1 k_{m_{M_1}}^{-1}+\gamma_{M_2}+\gamma_{M_3}\left(1+\theta_{M_3}\right)}}{1 + \frac{\beta_{M_1} G_1 k_{m_{M_1}}^{-1}}{1+G_1 k_{m_{M_1}}^{-1}+\gamma_{M_2}+\gamma_{M_3}} + \frac{\beta_{M_2}\gamma_{M_2}}{1+G_1 k_{m_{M_1}}^{-1}+\gamma_{M_2}+\gamma_{M_3}\left(1+\theta_{M_2}\right)} + \frac{\beta_{M_3}\gamma_{M_3}}{1+G_1 k_{m_{M_1}}^{-1}+\gamma_{M_2}+\gamma_{M_3}\left(1+\theta_{M_3}\right)}}$$

For fitting we again use the expression normalized to the first titration of miRFP670.

$$\hat{P}_1^* = \frac{G_1 \left(1 + G_{1_0} k_{m_{M_1}}^{-1} + \gamma_{M_2} + \gamma_{M_3}\right)}{G_{1_0} \left(1 + G_1 k_{m_{M_1}}^{-1} + \gamma_{M_2} + \gamma_{M_3}\right)} \frac{1 + \frac{\beta_{M_1} G_{1_0} k_{m_{M_1}}^{-1}}{1+G_{1_0} k_{m_{M_1}}^{-1}+\gamma_{M_2}+\gamma_{M_3}} + \frac{\beta_{M_2}\gamma_{M_2}}{1+G_{1_0} k_{m_{M_1}}^{-1}+\gamma_{M_2}+\gamma_{M_3}\left(1+\theta_{M_2}\right)} + \frac{\beta_{M_3}\gamma_{M_3}}{1+G_{1_0} k_{m_{M_1}}^{-1}+\gamma_{M_2}+\gamma_{M_3}\left(1+\theta_{M_3}\right)}}{1 + \frac{\beta_{M_1} G_1 k_{m_{M_1}}^{-1}}{1+G_1 k_{m_{M_1}}^{-1}+\gamma_{M_2}+\gamma_{M_3}} + \frac{\beta_{M_2}\gamma_{M_2}}{1+G_1 k_{m_{M_1}}^{-1}+\gamma_{M_2}+\gamma_{M_3}\left(1+\theta_{M_2}\right)} + \frac{\beta_{M_3}\gamma_{M_3}}{1+G_1 k_{m_{M_1}}^{-1}+\gamma_{M_2}+\gamma_{M_3}\left(1+\theta_{M_3}\right)}}$$

$$\hat{P}_2^* = \frac{1 + G_{1_0} k_{m_{M_1}}^{-1} + \gamma_{M_2} + \gamma_{M_3}\left(1 + \theta_{M_2}\right)}{1 + G_1 k_{m_{M_1}}^{-1} + \gamma_{M_2} + \gamma_{M_3}\left(1 + \theta_{M_2}\right)} \frac{1 + \frac{\beta_{M_1} G_{1_0} k_{m_{M_1}}^{-1}}{1+G_{1_0} k_{m_{M_1}}^{-1}+\gamma_{M_2}+\gamma_{M_3}} + \frac{\beta_{M_2}\gamma_{M_2}}{1+G_{1_0} k_{m_{M_1}}^{-1}+\gamma_{M_2}+\gamma_{M_3}\left(1+\theta_{M_2}\right)} + \frac{\beta_{M_3}\gamma_{M_3}}{1+G_{1_0} k_{m_{M_1}}^{-1}+\gamma_{M_2}+\gamma_{M_3}\left(1+\theta_{M_3}\right)}}{1 + \frac{\beta_{M_1} G_1 k_{m_{M_1}}^{-1}}{1+G_1 k_{m_{M_1}}^{-1}+\gamma_{M_2}+\gamma_{M_3}} + \frac{\beta_{M_2}\gamma_{M_2}}{1+G_1 k_{m_{M_1}}^{-1}+\gamma_{M_2}+\gamma_{M_3}\left(1+\theta_{M_2}\right)} + \frac{\beta_{M_3}\gamma_{M_3}}{1+G_1 k_{m_{M_1}}^{-1}+\gamma_{M_2}+\gamma_{M_3}\left(1+\theta_{M_3}\right)}}$$

$$\hat{P}_1^* = \frac{1 + G_{1_0} k_{m_{M_1}}^{-1} + \gamma_{M_2} + \gamma_{M_3}\left(1 + \theta_{M_3}\right)}{1 + G_1 k_{m_{M_1}}^{-1} + \gamma_{M_2} + \gamma_{M_3}\left(1 + \theta_{M_3}\right)} \frac{1 + \frac{\beta_{M_1} G_{1_0} k_{m_{M_1}}^{-1}}{1+G_{1_0} k_{m_{M_1}}^{-1}+\gamma_{M_2}+\gamma_{M_3}} + \frac{\beta_{M_2}\gamma_{M_2}}{1+G_{1_0} k_{m_{M_1}}^{-1}+\gamma_{M_2}+\gamma_{M_3}\left(1+\theta_{M_2}\right)} + \frac{\beta_{M_3}\gamma_{M_3}}{1+G_{1_0} k_{m_{M_1}}^{-1}+\gamma_{M_2}+\gamma_{M_3}\left(1+\theta_{M_3}\right)}}{1 + \frac{\beta_{M_1} G_1 k_{m_{M_1}}^{-1}}{1+G_1 k_{m_{M_1}}^{-1}+\gamma_{M_2}+\gamma_{M_3}} + \frac{\beta_{M_2}\gamma_{M_2}}{1+G_1 k_{m_{M_1}}^{-1}+\gamma_{M_2}+\gamma_{M_3}\left(1+\theta_{M_2}\right)} + \frac{\beta_{M_3}\gamma_{M_3}}{1+G_1 k_{m_{M_1}}^{-1}+\gamma_{M_2}+\gamma_{M_3}\left(1+\theta_{M_3}\right)}}$$

Supplementary Note 6. Models for DOX, HDV, RNA-binding protein and miRNA experiments.

Doxycycline (DOX) titration:
The system of ordinary differential equations used to obtain the steady state expressions is:

$$\dot{M}_1 = k_{M_1}^{\text{eff}}(G_1, G_1, G_2 f(P_1), G_2 f(P_1))G_1 - \delta_{M_1} M_1$$

$$\dot{M}_2 = k_{M_2}^{\text{eff}}(G_1, G_1, G_2 f(P_1), G_2 f(P_1))G_1 - \delta_{M_2} M_2$$

$$\dot{M}_3 = k_{M_3}^{\text{eff}}(G_1, G_1, G_2 f(P_1), G_2 f(P_1))G_2 f(P_1) - \delta_{M_3} M_3$$

$$\dot{M}_4 = k_{M_4}^{\text{eff}}(G_1, G_1, G_2 f(P_1), G_2 f(P_1))G_2 f(P_1) - \delta_{M_4} M_4$$

$$\dot{P}_1 = k_{P_1}^{\text{eff}}(M_1, M_2, M_3, M_4)M_1 - \delta_{P_1} P_1$$

$$\dot{P}_2 = k_{P_2}^{\text{eff}}(M_1, M_2, M_3, M_4)M_2 - \delta_{P_2} P_2$$

$$\dot{P}_3 = k_{P_3}^{\text{eff}}(M_1, M_2, M_3, M_4)M_3 - \delta_{P_3} P_3$$

$$\dot{P}_4 = k_{P_4}^{\text{eff}}(M_1, M_2, M_3, M_4)M_4 - \delta_{P_4} P_4$$

Here, the species M_1 and M_2 represent the mRNA species of the constitutively expressed transcriptional activator tagged with a fluorescent protein tTA-Cerulean (capacity monitor; P_1) and the fluorescent protein mCitrine (P_2). The species M_3 and M_4 represent the mRNA species of the two fluorescent proteins mRuby3 (X-tra; P_3) and miRFP670 (P_4). These two genes are expressed from a bidirectional tTA activatible promoter. By setting the left-hand side to zero, we can solve for the steady state of the protein species:

$$P_1^* = \frac{\alpha_{P_1} \frac{\beta_{M_1}\gamma_{M_1}}{1+\gamma_{M_1}+\gamma_{M_2}+(\gamma_{M_3}+\gamma_{M_4})f(P_1^*)}}{1 + \frac{\beta_{M_1}\gamma_{M_1}}{1+\gamma_{M_1}+\gamma_{M_2}+(\gamma_{M_3}+\gamma_{M_4})f(P_1^*)} + \frac{\beta_{M_2}\gamma_{M_2}}{1+\gamma_{M_1}+\gamma_{M_2}+(\gamma_{M_3}+\gamma_{M_4})f(P_1^*)} + \frac{\beta_{M_3}\gamma_{M_3}f(P_1^*)}{1+\gamma_{M_1}+\gamma_{M_2}+(\gamma_{M_3}+\gamma_{M_4})f(P_1^*)} + \frac{\beta_{M_4}\gamma_{M_4}f(P_1^*)}{1+\gamma_{M_1}+\gamma_{M_2}+(\gamma_{M_3}+\gamma_{M_4})f(P_1^*)}}$$

$$P_2^* = \frac{\alpha_{P_2} \frac{\beta_{M_2}\gamma_{M_2}}{1+\gamma_{M_1}+\gamma_{M_2}+(\gamma_{M_3}+\gamma_{M_4})f(P_1^*)}}{1 + \frac{\beta_{M_1}\gamma_{M_1}}{1+\gamma_{M_1}+\gamma_{M_2}+(\gamma_{M_3}+\gamma_{M_4})f(P_1^*)} + \frac{\beta_{M_2}\gamma_{M_2}}{1+\gamma_{M_1}+\gamma_{M_2}+(\gamma_{M_3}+\gamma_{M_4})f(P_1^*)} + \frac{\beta_{M_3}\gamma_{M_3}f(P_1^*)}{1+\gamma_{M_1}+\gamma_{M_2}+(\gamma_{M_3}+\gamma_{M_4})f(P_1^*)} + \frac{\beta_{M_4}\gamma_{M_4}f(P_1^*)}{1+\gamma_{M_1}+\gamma_{M_2}+(\gamma_{M_3}+\gamma_{M_4})f(P_1^*)}}$$

$$P_3^* = \frac{\alpha_{P_3} \frac{\beta_{M_3}\gamma_{M_3}f(P_1^*)}{1+\gamma_{M_1}+\gamma_{M_2}+(\gamma_{M_3}+\gamma_{M_4})f(P_1^*)}}{1 + \frac{\beta_{M_1}\gamma_{M_1}}{1+\gamma_{M_1}+\gamma_{M_2}+(\gamma_{M_3}+\gamma_{M_4})f(P_1^*)} + \frac{\beta_{M_2}\gamma_{M_2}}{1+\gamma_{M_1}+\gamma_{M_2}+(\gamma_{M_3}+\gamma_{M_4})f(P_1^*)} + \frac{\beta_{M_3}\gamma_{M_3}f(P_1^*)}{1+\gamma_{M_1}+\gamma_{M_2}+(\gamma_{M_3}+\gamma_{M_4})f(P_1^*)} + \frac{\beta_{M_4}\gamma_{M_4}f(P_1^*)}{1+\gamma_{M_1}+\gamma_{M_2}+(\gamma_{M_3}+\gamma_{M_4})f(P_1^*)}}$$

$$P_4^* = \frac{\alpha_{P_4} \frac{\beta_{M_4}\gamma_{M_4}f(P_1^*)}{1+\gamma_{M_1}+\gamma_{M_2}+(\gamma_{M_3}+\gamma_{M_4})f(P_1^*)}}{1 + \frac{\beta_{M_1}\gamma_{M_1}}{1+\gamma_{M_1}+\gamma_{M_2}+(\gamma_{M_3}+\gamma_{M_4})f(P_1^*)} + \frac{\beta_{M_2}\gamma_{M_2}}{1+\gamma_{M_1}+\gamma_{M_2}+(\gamma_{M_3}+\gamma_{M_4})f(P_1^*)} + \frac{\beta_{M_3}\gamma_{M_3}f(P_1^*)}{1+\gamma_{M_1}+\gamma_{M_2}+(\gamma_{M_3}+\gamma_{M_4})f(P_1^*)} + \frac{\beta_{M_4}\gamma_{M_4}f(P_1^*)}{1+\gamma_{M_1}+\gamma_{M_2}+(\gamma_{M_3}+\gamma_{M_4})f(P_1^*)}}$$

These expressions were fit to the data. The parameters were lumped according to:

$$\alpha_{P_i} := \frac{k_{P_i}^{\text{cat}} R_P^{\text{total}}}{\delta_{P_i}}$$

$$\beta_{M_i} := k_{m_{P_i}}^{-1} \frac{k_{M_i}^{\text{cat}} R_M^{\text{total}}}{\delta_{M_i}}$$

$$\gamma_{M_i} := k_{m_{M_i}}^{-1} G_i$$

Further, to reflect the inhibiting action of Doxycycline we use the product of an activating and a inhibiting hill-type function:

$$f(x, DOX) = \frac{x^{h_a}}{\kappa_a^{h_a} + x^{h_a}} \frac{\kappa_i^{h_i}}{\kappa_i^{h_i} + DOX^{h_i}}$$

HDV:
The system of ordinary differential equations used to obtain the steady state expressions is:

$$\dot{M}_1 = k_{M_1}^{\text{eff}}(G_1 f(P_1), G_1) G_1 f(P_2) - \delta_{M_1} M_1$$

$$\dot{M}_2 = k_{M_2}^{\text{eff}}(G_1 f(P_1), G_1) G_1 - \delta_{M_2} M_2$$

$$\dot{P}_1 = k_{P_1}^{\text{eff}}(M_1, M_2) M_1 - \delta_{P_1} P_1$$

$$\dot{P}_2 = k_{P_2}^{\text{eff}}(M_1, M_2) M_2 - \delta_{P_2} P_2$$

In these equations, the HDV-tagged mRNA species of X-tra (mCitrine) is denoted as M_1 and X-tra itself is denoted by P_1. Further, the species M_2 and P_2 represent the capacity monitor (tTA-P2A-mRuby3) mRNA and protein, respectively. To model the different expression of P_1 with and without the HDV ribozyme, we introduce an additional parameter ω_{M_1}. This parameter captures the reduction in the production rate of mature mRNA M_1 in the presence of the ribozyme. In the absence of the ribozyme, we set $\omega_{M_1} = 1$.

$$P_1^* = \frac{\alpha_{P_1} \frac{\frac{\beta_{M_1}}{\omega_{M_1}} \gamma_{M_1} f(P_2^*)}{1+\gamma_{M_1} f(P_2^*)+\gamma_{M_2}}}{1 + \frac{\frac{\beta_{M_1}}{\omega_{M_1}} \gamma_{M_1} f(P_2^*)}{1+\gamma_{M_1} f(P_2^*)+\gamma_{M_2}} + \frac{\beta_{M_2} \gamma_{M_2}}{1+\gamma_{M_1} f(P_2^*)+\gamma_{M_2}}}$$

$$P_2^* = \frac{\alpha_{P_2} \frac{\beta_{M_2} \gamma_{M_2}}{1+\gamma_{M_1} f(P_2^*)+\gamma_{M_2}}}{1 + \frac{\frac{\beta_{M_1}}{\omega_{M_1}} \gamma_{M_1} f(P_2^*)}{1+\gamma_{M_1} f(P_2^*)+\gamma_{M_2}} + \frac{\beta_{M_2} \gamma_{M_2}}{1+\gamma_{M_1} f(P_2^*)+\gamma_{M_2}}}$$

These expressions were fit to the data. The parameters were lumped according to the same definitions as in the Doxycycline titration model above.

RNA-binding proteins:
L7Ae:

The system of ordinary differential equations used to obtain the steady state expressions is:

$$\dot{M}_1 = k_{M_1}^{\text{eff}}(G_1, G_2, G_3)G_1 - (\delta_{M_1} + \eta P_3)M_1 + \nu C_{M_1,P_3}$$

$$\dot{M}_2 = k_{M_2}^{\text{eff}}(G_1, G_2, G_3)G_2 - \delta_{M_2}M_2$$

$$\dot{M}_3 = k_{M_3}^{\text{eff}}(G_1, G_2, G_3)G_3 - \delta_{M_3}M_3$$

$$\dot{P}_1 = k_{P_1}^{\text{eff}}(M_1, M_2, M_3)M_1 - \delta_{P_1}P_1$$

$$\dot{P}_2 = k_{P_2}^{\text{eff}}(M_1, M_2, M_3)M_2 - \delta_{P_2}P_2$$

$$\dot{P}_3 = k_{P_3}^{\text{eff}}(M_1, M_2, M_3)M_3 - \delta_{P_3}P_3$$

$$\dot{C}_{M_1,P_3} = \eta M_1 P_3 - (\nu + \delta_{C_{M_1,P_3}})C_{M_1,P_3}$$

In this set of equations, M_1 denotes the mRNA species of the X-tra protein P_1. The capacity monitor mRNA is represented by M_2 and its protein by P_2. The RNA-binding protein L7Ae is denote by P_3 and its mRNA species is given by M_3.

$$P_1^* = \frac{\alpha_{P_1}\frac{\beta_{M_1}\gamma_{M_1}(1+\nu)}{(1+\gamma_{M_1}+\gamma_{M_2}+\gamma_{M_3})(1+\nu+\rho_{M_1}P_3^*)}}{1+\frac{\beta_{M_1}\gamma_{M_1}(1+\nu)}{(1+\gamma_{M_1}+\gamma_{M_2}+\gamma_{M_3})(1+\nu+\rho_{M_1}P_3^*)}+\frac{\beta_{M_2}\gamma_{M_2}}{1+\gamma_{M_1}+\gamma_{M_2}+\gamma_{M_3}}+\frac{\beta_{M_3}\gamma_{M_3}}{1+\gamma_{M_1}+\gamma_{M_2}+\gamma_{M_3}}}$$

$$P_2^* = \frac{\alpha_{P_2}\frac{\beta_{M_2}\gamma_{M_2}}{1+\gamma_{M_1}+\gamma_{M_2}+\gamma_{M_3}}}{1+\frac{\beta_{M_1}\gamma_{M_1}(1+\nu)}{(1+\gamma_{M_1}+\gamma_{M_2}+\gamma_{M_3})(1+\nu+\rho_{M_1}P_3^*)}+\frac{\beta_{M_2}\gamma_{M_2}}{1+\gamma_{M_1}+\gamma_{M_2}+\gamma_{M_3}}+\frac{\beta_{M_3}\gamma_{M_3}}{1+\gamma_{M_1}+\gamma_{M_2}+\gamma_{M_3}}}$$

$$P_3^* = \frac{\alpha_{P_3}\frac{\beta_{M_3}\gamma_{M_3}}{1+\gamma_{M_1}+\gamma_{M_2}+\gamma_{M_3}}}{1+\frac{\beta_{M_1}\gamma_{M_1}(1+\nu)}{(1+\gamma_{M_1}+\gamma_{M_2}+\gamma_{M_3})(1+\nu+\rho_{M_1}P_3^*)}+\frac{\beta_{M_2}\gamma_{M_2}}{1+\gamma_{M_1}+\gamma_{M_2}+\gamma_{M_3}}+\frac{\beta_{M_3}\gamma_{M_3}}{1+\gamma_{M_1}+\gamma_{M_2}+\gamma_{M_3}}}$$

Here, we introduce a new lumped parameter $\rho_{M_1} := \frac{\eta}{\delta_{M_1}}$. Due to the complexity of the expressions, the system has not been fully solved for its steady state. To obtain the expressions used for fitting to the data, the steady state expression for P_3^* above was solved for P_3^* and plugged into the steady state expressions of P_1^* and P_2^*.

Ms2-cNOT7:
The system of ordinary differential equations used to obtain the steady state expressions is:

$$\dot{M}_1 = k_{M_1}^{\text{eff}}(G_1, G_2, G_3)G_1 - (\delta_{M_1} + \eta P_3)M_1$$

$$\dot{M}_2 = k_{M_2}^{\text{eff}}(G_1, G_2, G_3)G_2 - \delta_{M_2}M_2$$

$$\dot{M}_3 = k_{M_3}^{\text{eff}}(G_1, G_2, G_3)G_3 - \delta_{M_3}M_3$$

$$\dot{P}_1 = k_{P_1}^{\text{eff}}(M_1, M_2, M_3)M_1 - \delta_{P_1}P_1$$

$$\dot{P}_2 = k_{P_2}^{\text{eff}}(M_1, M_2, M_3)M_2 - \delta_{P_2}P_2$$

$$\dot{P}_3 = k_{P_3}^{\text{eff}}(M_1, M_2, M_3)M_3 - \delta_{P_3}P_3$$

Again, M_1 represents the mRNA and P_1 the protein of X-tra. M_2 and P_2 denote the mRNA and the protein of the capacity monitor. The RNA-binding protein Ms2-cNOT7 is captured in P_3, with the mRNA species M_3. Solving the above system for steady state yields.

$$P_1^* = \frac{\alpha_{P_1}\frac{\beta_{M_1}\gamma_{M_1}}{(1+\gamma_{M_1}+\gamma_{M_2}+\gamma_{M_3})(1+\rho_{M_1}P_3^*)}}{1+\frac{\beta_{M_1}\gamma_{M_1}}{(1+\gamma_{M_1}+\gamma_{M_2}+\gamma_{M_3})(1+\rho_{M_1}P_3^*)}+\frac{\beta_{M_2}\gamma_{M_2}}{1+\gamma_{M_1}+\gamma_{M_2}+\gamma_{M_3}}+\frac{\beta_{M_3}\gamma_{M_3}}{1+\gamma_{M_1}+\gamma_{M_2}+\gamma_{M_3}}}$$

$$P_2^* = \frac{\alpha_{P_2}\frac{\beta_{M_2}\gamma_{M_2}}{1+\gamma_{M_1}+\gamma_{M_2}+\gamma_{M_3}}}{1+\frac{\beta_{M_1}\gamma_{M_1}}{(1+\gamma_{M_1}+\gamma_{M_2}+\gamma_{M_3})(1+\rho_{M_1}P_3^*)}+\frac{\beta_{M_2}\gamma_{M_2}}{1+\gamma_{M_1}+\gamma_{M_2}+\gamma_{M_3}}+\frac{\beta_{M_3}\gamma_{M_3}}{1+\gamma_{M_1}+\gamma_{M_2}+\gamma_{M_3}}}$$

$$P_3^* = \frac{\alpha_{P_3}\frac{\beta_{M_3}\gamma_{M_3}}{1+\gamma_{M_1}+\gamma_{M_2}+\gamma_{M_3}}}{1+\frac{\beta_{M_1}\gamma_{M_1}}{(1+\gamma_{M_1}+\gamma_{M_2}+\gamma_{M_3})(1+\rho_{M_1}P_3^*)}+\frac{\beta_{M_2}\gamma_{M_2}}{1+\gamma_{M_1}+\gamma_{M_2}+\gamma_{M_3}}+\frac{\beta_{M_3}\gamma_{M_3}}{1+\gamma_{M_1}+\gamma_{M_2}+\gamma_{M_3}}}$$

As before, the expressions fit to the data are obtained by solving the equation for P_3^* for P_3^* and plugin the result into the other two expressions.

miRNA:
The system of ordinary differential equations used to obtain the steady state expressions is:

$$\dot{M}_1 = k_{M_1}^{\text{eff}}(G_1, G_1, G_m)G_1 - (\delta_{M_1} + \eta m)M_1$$

$$\dot{M}_2 = k_{M_2}^{\text{eff}}(G_1, G_1, G_m)G_1 - \delta_{M_2}M_2$$

$$\dot{m} = k_{m}^{\text{eff}}(G_1, G_1, G_m)G_m - \delta_{m}m$$

$$\dot{P}_1 = k_{P_1}^{\text{eff}}(M_1, M_2)M_1 - \delta_{P_1}P_1$$

$$\dot{P}_2 = k_{P_2}^{\text{eff}}(M_1, M_2)M_2 - \delta_{P_2}P_2$$

In this system of equations, we denote the miRNA sensor mRNA by M_1 and the respective fluorescent protein output as P_1. The capacity monitor is expressed through the mRNA M_2 and the protein P_2. The miRNA itself is represented by m. Solving for steady state yields:

$$P_1^* = \frac{\alpha_{P_1}\frac{\beta_{M_1}\gamma_{M_1}}{1+\gamma_{M_1}+\gamma_{M_2}+\gamma_m(1+\theta_{M_1}\lambda\tau)}}{1+\frac{\beta_{M_1}\gamma_{M_1}}{1+\gamma_{M_1}+\gamma_{M_2}+\gamma_m}+\frac{\beta_{M_2}\gamma_{M_2}}{1+\gamma_{M_1}+\gamma_{M_2}+\gamma_m(1+\theta_{M_1}\lambda\tau)}}$$

$$P_2^* = \frac{\alpha_{P_2} \frac{\beta_{M_2}\gamma_{M_2}}{1+\gamma_{M_1}+\gamma_{M_2}+\gamma_m}}{1 + \frac{\beta_{M_1}\gamma_{M_1}}{1+\gamma_{M_1}+\gamma_{M_2}+\gamma_m} + \frac{\beta_{M_2}\gamma_{M_2}}{1+\gamma_{M_1}+\gamma_{M_2}+\gamma_m(1+\theta_{M_1}\lambda\tau)}}$$

We use the additional lumping parameter:

$$\theta_{M_i} := \frac{\eta_{M_i}}{\delta_{M_i}} \frac{k_{M_2}^{\mathrm{cat}} R_M^{\mathrm{total}}}{\delta_m}$$

To account for the placement of the miRNA targets in different UTRs and different numbers of targets we introduce the two parameters λ (UTR) and τ (target number). Specifically, $\lambda \in \{\lambda_{3'}, \lambda_{5'}\}$ and $\tau \in \{\tau_{1x}, \tau_{3x}\}$.

Supplementary Note 7. Use of geometric means as a relative measure of cellular capacity.

To show how the ratio of geometric means measures gene expression capacity we consider a simple model of gene expression:

$$\dot{C}_{M_i} = k^{+}_{M_i} G_i R_{f_M} - (k^{-}_{M_i} + k^{\mathrm{cat}}_{M_i}) C_{M_i}$$

$$\dot{M}_i = k^{\mathrm{cat}}_{M_i} C_{M_i} - \delta_{M_i} M_i$$

$$\dot{C}_{P_i} = k^{+}_{P_i} M_i R_{f_P} - (k^{-}_{P_i} + k^{\mathrm{cat}}_{P_i}) C_{P_i}$$

$$\dot{P}_i = k^{\mathrm{cat}}_{P_i} C_{P_i} - \delta_{P_i} P_i$$

In this model, gene G_i can bind free transcriptional resources R_{f_M}. This forms the complex C_{M_i} with rate $k^{+}_{M_i}$. This complex can disassociate with rate $k^{-}_{M_i}$ or give rise to the mRNA species M_i with rate k^{cat}_{i}. The mRNA species M_i may also degrade with rate δ_{M_i}. Analogously to this process, the mRNA species can bind free translational resources R_{f_P} to form the complex C_{P_i} with rate $k^{+}_{P_i}$. Again, this complex may dissociate with rate $k^{-}_{P_i}$ or produce the protein species P_i. This protein species is removed with rate δ_{P_i}. When the left-hand side of the equations above are set to zero, we can solve for the steady-state expression for the protein species:

$P^*_i = \alpha_i G_i R^*_{f_M} R^*_{f_P}$ with $\alpha_i := \frac{k^{\mathrm{cat}}_{P_i}}{\delta_{P_i}} \frac{k^{+}_{P_i}}{k^{-}_{P_i} + k^{\mathrm{cat}}_{P_i}} \frac{k^{+}_{M_i}}{k^{-}_{M_i} + k^{\mathrm{cat}}_{M_i}}$.

By treating G_i, $R^*_{f_M}$, $R^*_{f_P}$ and P^*_i as random variables we can write the geometric mean of P^*_i as:

$$e^{\mathrm{E}[\log(P^*_i)]} = e^{\mathrm{E}[\log(\alpha_i G_i R^*_{f_M} R^*_{f_P})]}$$

$$= e^{\mathrm{E}[\log(\alpha_i) + \log(G_i) + \log(R^*_{f_M}) + \log(R^*_{f_P})]}$$

$$= e^{\log(\alpha_i) + \mathrm{E}[\log(G_i)] + \mathrm{E}[\log(R^*_{f_M})] + \mathrm{E}[\log(R^*_{f_P})]}$$

$$= \alpha_i e^{\mathrm{E}[\log(G_i)]} e^{\mathrm{E}[\log(R^*_{f_M})]} e^{\mathrm{E}[\log(R^*_{f_P})]}$$

If we consider P^*_i as our capacity monitor, which we use to measure the change in gene expression capacity relative to the capacity monitor measures for a baseline capacity $P^*_{i,0}$, then we write:

$$\frac{e^{\mathrm{E}[\log(P^*_i)]}}{e^{\mathrm{E}[\log(P^*_{i,0})]}} = \frac{\alpha_i e^{\mathrm{E}[\log(G_i)]} e^{\mathrm{E}[\log(R^*_{f_M})]} e^{\mathrm{E}[\log(R^*_{f_P})]}}{\alpha_i e^{\mathrm{E}[\log(G_i)]} e^{\mathrm{E}[\log(R^*_{f_M,0})]} e^{\mathrm{E}[\log(R^*_{f_P,0})]}}$$

Assuming that the plasmid take-up distribution of the capacity monitor plasmid G_i does not change across samples we get:

$$= \frac{e^{\mathrm{E}[\log(R^*_{f_M})]} e^{\mathrm{E}[\log(R^*_{f_P})]}}{e^{\mathrm{E}[\log(R^*_{f_M,0})]} e^{\mathrm{E}[\log(R^*_{f_P,0})]}}$$

$$= \frac{e^{\mathrm{E}[\log(R^*_{f_M})]+\mathrm{E}[\log(R^*_{f_P})]}}{e^{\mathrm{E}[\log(R^*_{f_M,0})]+\mathrm{E}[\log(R^*_{f_P,0})]}}$$

$$= \frac{e^{\mathrm{E}[\log(R^*_{f_M})+\log(R^*_{f_P})]}}{e^{\mathrm{E}[\log(R^*_{f_M,0})+\log(R^*_{f_P,0})]}}$$

$$= \frac{e^{\mathrm{E}[\log(R^*_{f_M} R^*_{f_P})]}}{e^{\mathrm{E}[\log(R^*_{f_M,0} R^*_{f_P,0})]}}$$

$$e^{\mathrm{E}[\log(P^*_i)]-\mathrm{E}[\log(P^*_{i,0})]} = e^{\mathrm{E}[\log(R^*_{f_M} R^*_{f_P})]-\mathrm{E}[\log(R^*_{f_M,0} R^*_{f_P,0})]}$$

$$e^{\mathrm{E}[\log(P^*_i)-\log(P^*_{i,0})]} = e^{\mathrm{E}[\log(R^*_{f_M} R^*_{f_P})-\log(R^*_{f_M,0} R^*_{f_P,0})]}$$

$$e^{\mathrm{E}[\log(\frac{P^*_i}{P^*_{i,0}})]} = e^{\mathrm{E}[\log(\frac{R^*_{f_M} R^*_{f_P}}{R^*_{f_M,0} R^*_{f_P,0}})]}$$

This shows that our approach can report on relative changes in available free resources. In comparison, when the same analysis is performed with the same assumptions but the arithmetic mean is used instead of the geometric mean we get:

$$\frac{\mathrm{E}[P^*_i]}{\mathrm{E}[P^*_{i,0}]} = \frac{\mathrm{E}[\alpha_i G_i R^*_{f_M} R^*_{f_P}]}{\mathrm{E}[\alpha_i G_i R^*_{f_M,0} R^*_{f_P,0}]}$$

$$= \frac{\mathrm{E}[G_i R^*_{f_M} R^*_{f_P}]}{\mathrm{E}[G_i R^*_{f_M,0} R^*_{f_P,0}]}$$

This expression is analogous to the ratio of the weighted arithmetic mean, where the weighting is given by G_i. If we consider the gene of the capacity monitor to be integrated, the expression simplifies to:

$$\frac{\mathrm{E}[P^*_i]}{\mathrm{E}[P^*_{i,0}]} = \frac{\mathrm{E}[R^*_{f_M} R^*_{f_P}]}{\mathrm{E}[R^*_{f_M,0} R^*_{f_P,0}]}$$

This holds because the gene copy number G_i is fixed after integration and can be pulled out of the expected value. The expression shows that integration of the capacity monitor into the genome of a cell would permit a more direct way of measuring the cellular capacity.

Supplementary Table 1. Transfection tables for all experiments in this study.

Figure 2a

500 ng total	pGLM49	pTTF72	pGLM171
1:1	62.5 ng	62.5 ng	375 ng
1:2	62.5 ng	125 ng	312.5 ng
1:3	62.5 ng	187.5 ng	250 ng
1:4	62.5 ng	250 ng	187.5 ng
2:1	125 ng	62.5 ng	312.5 ng
2:2	125 ng	125 ng	250 ng
2:3	125 ng	187.5 ng	187.5 ng
2:4	125 ng	250 ng	125 ng
3:1	187.5 ng	62.5 ng	250 ng
3:2	187.5 ng	125 ng	187.5 ng
3:3	187.5 ng	187.5 ng	125 ng
3:4	187.5 ng	250 ng	62.5 ng
4:1	250 ng	62.5 ng	187.5 ng
4:2	250 ng	125 ng	125 ng
4:3	250 ng	187.5 ng	62.5 ng
4:4	250 ng	250 ng	0 ng
Reagent/cells			
Optimem	to 50 µL		
PEI	1.5 µL		
HEK293T	62500		

50 ng total	pGLM49	pTTF72	pGLM171
1:1	6.25 ng	6.25 ng	487.5 ng
1:2	6.25 ng	12.5 ng	481.25 ng
1:3	6.25 ng	18.75 ng	475 ng
1:4	62.5 ng	25 ng	468.75 ng
2:1	12.5 ng	6.25 ng	481.25 ng
2:2	12.5 ng	12.5 ng	475 ng
2:3	12.5 ng	18.75 ng	468.75 ng
2:4	12.5 ng	25 ng	462.5 ng

3:1	18.75 ng	6.25 ng	475 ng
3:2	18.75 ng	12.5 ng	468.75 ng
3:3	18.75 ng	18.75 ng	462.5 ng
3:4	18.75 ng	25 ng	456.25 ng
4:1	25 ng	6.25 ng	468.75 ng
4:2	25 ng	12.5 ng	462.5 ng
4:3	25 ng	18.75 ng	456.25 ng
4:4	25 ng	25 ng	450 ng
Reagent/cells			
Optimem	to 50 µL		
PEI	1.5 µL		
HEK293T	62500		

Supplementary figure 2

	pTTF57	pTTF181	pGLM49	pTTF72	pGLM171
EFS/EFS	174.9 ng	174.8 ng	0 ng	0 ng	96.8 ng
EF1a/EFS	0 ng	174.8 ng	215.0 ng	0 ng	56.7 ng
EFS/EF1a	174.9 ng	0 ng	0 ng	215.5 ng	56.0 ng
EF1a/EF1a	0 ng	0 ng	215.0 ng	215.5 ng	15.9 ng
Reagent/cells					
Optimem	to 50 µL				
PEI	1.5 µL				
HEK293T	50000/75000				

Supplementary figure 3

	pGLM49	pGLM177	pGLM171
0.25	104.9 ng	32.5 ng	162.4 ng
0.50	104.9 ng	64.9 ng	129.9 ng
0.75	104.9 ng	97.4 ng	97.4 ng
1.00	104.9 ng	129.9 ng	64.9 ng
1.25	104.9 ng	162.4 ng	32.5 ng
1.50	104.9 ng	194.8 ng	0 ng
Reagent/cells			
Optimem	to 30 µL		
PEI	0.9 µL		
HEK293T	75000		

Figure 2b

	pGLM49	pTTF72	pGLM171
1:1	62.5 ng	62.5 ng	375 ng
Reagent/cells			
Optimem	to 50 µL		
PEI	1.5 µL		
HEK293T	62500		

Supplementary figure 4

	pTTF194	pTTF72	pGLM171
2:4	112.7 ng	156.1 ng	229.2 ng
4:4	225.3 ng	156.1 ng	114.6 ng
6:4	338 ng	156.1 ng	0 ng
Reagent/cells			
Optimem	to 50 µL		
PEI	1.5 µL		
HEK293T	50000/75000		

Figure 2c and supplementary figure 1b-c, 8a, 9a, 10a

	pL-A1	pBI-G	pEMPTY
1.0	120 ng	120 ng	360 ng
1.5	120 ng	180 ng	200 ng
2.0	120 ng	240 ng	140 ng
2.5	120 ng	300 ng	80 ng
Reagent/cells			
Optimem	50 µL		
Lipofectemine 3000	0.75 µL		
P3000	1 µL		
H1299	150000		
U2OS	200000		
HeLa	200000		
HEK293T	150000		
CHO-K1	150000		

Supplementary figure 1d-e

	ai274	pBI-G	pEMPTY
1.0	120 ng	120 ng	360 ng
1.5	120 ng	180 ng	200 ng
2.0	120 ng	240 ng	140 ng
2.5	120 ng	300 ng	80 ng
Reagent/cells			
Optimem	50 µL		
Lipofectemine 3000	0.75 µL		
P3000	1 µL		
H1299	150000		
HEK	150000		

Figure 2d and supplementary figure 5, 23, 24

	pBI-F3G	pBI-H3G
noTS	500 ng	
miR31 iFFL		500 ng
Reagent/cells		
Optimem	250 µL	
Lipofectemine 3000	11 µL	
P3000	8.25 µL	
H1299	1650000	

Figure 2e and supplementary figure 7

	pTTF218	pTTF219
HDV (-)	0 ng	500 ng
HDV (+)	500 ng	0 ng
Reagent/cells		
Optimem	to 50 µL	
PEI	1.5 µL	
HEK293T	70000	

Figure 2f and supplementary figure 8b, 9b, 10b, 11b

	pL-A1	Oipron	pBI-G	pEMPTY
(-) synthetic intron	50 ng		50 ng	200 ng
(+) synthtic intron		50 ng	50 ng	200 ng
Reagent/cells				
Optimem	50 µL			
Lipofectemine 3000	0.75 µL			
P3000	1 µL			
H1299	150000			
HEK293T	150000			
U2OS	200000			
HeLa	200000			
CHO-K1	150000			

Figure 2g and supplementary figure 8c, 9c, 10c, 11c

	pL-S1	pL-C1	p125	pL-R1	pL-A1	pEMPTY
L7Ae control	50 ng				50 ng	200 ng
L7Ae	50 ng		50 ng		50 ng	150 ng
Ms2-cNOT7 control		50 ng			50 ng	200 ng
Ms2-cNOT7		50 ng		50 ng	50 ng	150 ng
Reagent/cells						
Optimem	50 µL					
Lipofectemine 3000	0.75 µL					
P3000	1 µL					
H1299	150000					
HEK	150000					
U2OS	200000					
HeLa	200000					
CHO-K1	150000					

Figure 2h and supplementary figure 8d, 9d, 10d, 11d

	pL-A1	pH-7	pH-22	pH-14	pL-S1	pEMPTY
H1299 control	50 ng				50 ng	200 ng
H1299 reppressed		50 ng			50 ng	200 ng
U2OS control	50 ng				50 ng	200 ng
U2OS repressed			50 ng		50 ng	200 ng
HeLa control	50 ng				50 ng	200 ng
HeLa repressed				50 ng	50 ng	200 ng
Reagent/cells						
Optimem	50 µL					
Lipofectemine 2000	1.5 µL					
P3000	1 µL					
H1299	150000					
HeLa	200000					
U2-OS	200000					

Figure 3b,c and supplementary figure 12

	pBI-F3G	pBI-H1G/pBI-H3G/pBI-H5G/pBI-H7G	pEMPTY
Control	100 ng		200 ng
miR-31 TS		100 ng	200 ng
miR-31 TS + inhibitor (20pmol)		100 ng	200 ng
Reagent/cells			
Optimem	50 µL		
Lipofectemine 3000	0.75 µL		
P3000	1 µL		
H1299	150000		

Supplementary figure 13

	pL-A1	pH-1/pH-2/pH-3/pH-5/pH-6/pH-7	pL-S1	pEMPTY
noTS	50 ng		50 ng	200 ng
miR-31 TS		50 ng	50 ng	200 ng
Reagent/cells				
Optimem	50 µL			
Lipofectemine 3000	0.75 µL			
P3000	1 µL			
H1299	150000			

Supplementary figure 14

	pL-A1	pH-31	pL-S1	pEMPTY
noTS	50 ng		50 ng	200 ng
miR-31 TS		50 ng	50 ng	200 ng
Reagent/cells				
Optimem	50 µL			
Lipofectemine 3000	0.75 µL			
P3000	1 µL			
HEK293T	150000			
U2-OS	200000			

Supplementary figure 15, 16

**SF 16**	pL-A1	pH-16/pH-17/pH-18/pH-20/pH-21/pH-22	pL-S1	pEMPTY
noTS	50 ng		50 ng	200 ng
miR-221 TS		50 ng	50 ng	200 ng
**SF 17**	pL-A1	pH-8/pH-9/pH-10/pH-12/pH-13/pH-14	pL-S1	pEMPTY
noTS	50 ng		50 ng	200 ng
miR-21 TS		50 ng	50 ng	200 ng
Reagent/cells				
Optimem	50 µL			
Lipofectemine 3000	0.75 µL			
HeLa	200000			
U2-OS	200000			

Figure 4

	pGLM49	pGLM91	pGLM92	pGLM102	pGLM103	pGLM171
OLP 0 ng	140 ng	0 ng	180 ng	0 ng	0 ng	180 ng
OLP 20 ng	140 ng	0 ng	180 ng	0 ng	20 ng	140 ng
OLP 60 ng	140 ng	0 ng	180 ng	0 ng	60 ng	100 ng
OLP 100 ng	140 ng	0 ng	180 ng	0 ng	100 ng	60 ng
OLP 140 ng	140 ng	0 ng	180 ng	0 ng	140 ng	20 ng
OLP 180 ng	140 ng	0 ng	180 ng	0 ng	180 ng	0 ng
IFF 0 ng	140 ng	180 ng	0 ng	0 ng	0 ng	180 ng
IFF 20 ng	140 ng	180 ng	0 ng	0 ng	20 ng	140 ng
IFF 60 ng	140 ng	180 ng	0 ng	0 ng	60 ng	100 ng
IFF 100 ng	140 ng	180 ng	0 ng	0 ng	100 ng	60 ng
IFF 140 ng	140 ng	180 ng	0 ng	0 ng	140 ng	20 ng
IFF 180 ng	140 ng	180 ng	0 ng	0 ng	180 ng	0 ng
FBK 0 ng	140 ng	0 ng	180 ng	0 ng	0 ng	180 ng
FBK 20 ng	140 ng	0 ng	180 ng	20 ng	0 ng	140 ng
FBK 60 ng	140 ng	0 ng	180 ng	60 ng	0 ng	100 ng
FBK 100 ng	140 ng	0 ng	180 ng	100 ng	0 ng	60 ng
FBK 140 ng	140 ng	0 ng	180 ng	140 ng	0 ng	20 ng
FBK 180 ng	140 ng	0 ng	180 ng	180 ng	0 ng	0 ng
HYB 0 ng	140 ng	180 ng	0 ng	0 ng	0 ng	180 ng
HYB 20 ng	140 ng	180 ng	0 ng	20 ng	0 ng	140 ng
HYB 60 ng	140 ng	180 ng	0 ng	60 ng	0 ng	100 ng
HYB 100 ng	140 ng	180 ng	0 ng	100 ng	0 ng	60 ng
HYB 140 ng	140 ng	180 ng	0 ng	140 ng	0 ng	20 ng
HYB 180 ng	140 ng	180 ng	0 ng	180 ng	0 ng	0 ng
Reagent/cells						
Optimem	to 50 µL					
PEI	1.5 µL					
HEK293T	62500					

Figure 5c and supplementary figure 18, 19, 20, 22

	pL-A1	p-H3/p-H18	pBI-G	pEMPTY
0.25 noTS	120 ng		30 ng	150 ng
0.25 miR-TS		120 ng	30 ng	150 ng
0.50 noTS	120 ng		60 ng	120 ng
0.50 miR-TS		120 ng	60 ng	120 ng
0.75 noTS	120 ng		90 ng	90 ng
0.75 miR-TS		120 ng	90 ng	90 ng
1.00 noTS	120 ng		120 ng	60 ng
1.00 miR-TS		120 ng	120 ng	60 ng
1.25 noTS	120 ng		150 ng	30 ng
1.25 miR-TS		120 ng	150 ng	30 ng
1.50 noTS	120 ng		180 ng	
1.50 miR-TS		120 ng	180 ng	
Reagent/cells				
Optimem	50 µL			
Lipofectemine 3000	0.75 µL			
P3000	1 µL			
H1299	150000			
U2OS	200000			
HEK293T	150000			

Supplementary figure 21

	pL-A1	pH-18	pL-S1	pEMPTY
noTS	50 ng		50 ng	200 ng
miR-221 TS		50 ng	50 ng	200 ng
Reagent/cells				
Optimem	50 µL			
Lipofctemine 3000	0.75 µL			
P3000	1 µL			
HEK	150000			

Figure 5d

	pTTF220	pTTF223	pTTF138	pGLM171
0.25 mCit.-3xTFF5/mRub.-3xTFF5	155.1 ng	0 ng	24.2 ng	120.7 ng
0.50 mCit.-3xTFF5/mRub.-3xTFF5	155.1 ng	0 ng	48.3 ng	96.6 ng
0.75 mCit.-3xTFF5/mRub.-3xTFF5	155.1 ng	0 ng	72.5 ng	72.4 ng
1.00 mCit.-3xTFF5/mRub.-3xTFF5	155.1 ng	0 ng	96.6 ng	48.3 ng
1.25 mCit.-3xTFF5/mRub.-3xTFF5	155.1 ng	0 ng	120.8 ng	24.1 ng
1.50 mCit.-3xTFF5/mRub.-3xTFF5	155.1 ng	0 ng	144.9 ng	0 ng
0.25 mCit.-3xTFF4/mRub.-3xTFF4	0 ng	155.1 ng	24.2 ng	120.7 ng
0.50 mCit.-3xTFF4/mRub.-3xTFF4	0 ng	155.1 ng	48.3 ng	96.6 ng
0.75 mCit.-3xTFF4/mRub.-3xTFF4	0 ng	155.1 ng	72.5 ng	72.4 ng
1.00 mCit.-3xTFF4/mRub.-3xTFF4	0 ng	155.1 ng	96.6 ng	48.3 ng
1.25 mCit.-3xTFF4/mRub.-3xTFF4	0 ng	155.1 ng	120.8 ng	24.1 ng
1.50 mCit.-3xTFF4/mRub.-3xTFF4	0 ng	155.1 ng	144.9 ng	0 ng
Reagent/cells				
Optimem	to 50 µL			
Lipofectamine 2000	0.6 µL			
mES E14	70000			

Supplementary figure 25

	pTTF220	pTTF223	pTTF138	pGLM171
0.25 mCit.-3xTFF5/mRub.-3xTFF5	258.4 ng	0 ng	40.3 ng	201.3 ng
0.50 mCit.-3xTFF5/mRub.-3xTFF5	258.4 ng	0 ng	80.5 ng	161.1 ng
0.75 mCit.-3xTFF5/mRub.-3xTFF5	258.4 ng	0 ng	120.8 ng	120.8 ng
1.00 mCit.-3xTFF5/mRub.-3xTFF5	258.4 ng	0 ng	161.0 ng	80.6 ng
1.25 mCit.-3xTFF5/mRub.-3xTFF5	258.4 ng	0 ng	201.3 ng	40.3 ng
1.50 mCit.-3xTFF5/mRub.-3xTFF5	258.4 ng	0 ng	241.6 ng	0 ng
0.25 mCit.-3xTFF4/mRub.-3xTFF4	0 ng	258.4 ng	40.3 ng	201.3 ng
0.50 mCit.-3xTFF4/mRub.-3xTFF4	0 ng	258.4 ng	80.5 ng	161.1 ng
0.75 mCit.-3xTFF4/mRub.-3xTFF4	0 ng	258.4 ng	120.8 ng	120.8 ng
1.00 mCit.-3xTFF4/mRub.-3xTFF4	0 ng	258.4 ng	161.0 ng	80.6 ng
1.25 mCit.-3xTFF4/mRub.-3xTFF4	0 ng	258.4 ng	201.3 ng	40.3 ng
1.50 mCit.-3xTFF4/mRub.-3xTFF4	0 ng	258.4 ng	241.6 ng	0 ng
Reagent/cells				
Optimem	to 50 µL			
PEI	1.5 µL			
HEK293T	70000			

Supplementary Table 2. List of the plasmids used in this study. Plasmids sequences are available on GenBank.

Fig.	Short plasmid name	Full plasmid name	Parts from	GenBank accession code
2c-e-f-g, 5b & SF1, 8-11, 13-16, 18-21	pL-A1	pT-GTW6-CMV-mKate	[2]	MT891367
SF1	ai274	pT-PGK-mCherry	[3]	MT891341
2f & SF8-11	Oipron	pT-GTW6-CMV-SImKate	[4]	
2c-e, 5c & SF1, 8-11, 18-19	pBI-G	pBI-CMV1_EGFP	Clontech 631630	MT891343
2g-h & SF8-11, 13-16, 20	pL-S1	pBoxCDGC_2xKMet_EGFP	[5]	MT891368
2g & SF8-11	pL-C1	pBoxCDGCmut_KMetEGFP-8xMS2-pA	[5]	MH883358
2g & SF8-11	p125	pT-GTW6-CMV-L7AescFv35	[5]	MH883336
2g & SF8-11	pL-R1	pT-GTW6-CMV-MS2- CNOT7	[5]	MH883359
SF13	pH-1	pT-GTW6-CMV-mKate_1xmiR31TS5'	[6]	MT891348
SF13	pH-2	pT-GTW6-CMV-mKate_2xmiR31TS5'	[6]	MT891349
5c & SF13	pH-3	pT-GTW6-CMV-mKate_3xmiR31TS5'	[6]	MT891350
SF13	pH-5	pT-GTW6-CMV-mKate_1xmiR31TS3'	[6]	MT891351
SF13	pH-6	pT-GTW6-CMV-mKate_2xmiR31TS3'	[6]	MT891352
SF8, 13-14	pH-7	pT-GTW6-CMV-mKate_3xmiR31TS3'	[6]	MT891353
2d, 3 & SF5, 12, 23-24	pBI-F3G	pBI-CMV1_EGFP_mKate	Clontech 631630	MT891342
3 & SF12	pBI-H1G	pBI-CMV1_EGFP_mKate_1xmiR31TS5'	Clontech 631630	MT891344
3 & SF5, 12, 23-24	pBI-H3G	pBI-CMV1_EGFP_mKate_3xmiR31TS5'	Clontech 631630	MT891345

3 & SF12	pBI-H5G	pBI-CMV1_EGFP_mKate_1xmiR31TS3'	Clontech 631630	MT891346
3	pBI-H7G	pBI-CMV1_EGFP_mKate_3xmiR31TS3'	Clontech 631630	MT891347
SF15	pH-8	pT-GTW6-CMV-mKate_1xmiR21TS5'	[6]	MT891354
SF15	pH-9	pT-GTW6-CMV-mKate_2xmiR21TS5'	[6]	MT891355
SF15	pH-10	pT-GTW6-CMV-mKate_3xmiR21TS5'	[6]	MT891356
SF15	pH-12	pT-GTW6-CMV-mKate_1xmiR21TS3'	[6]	MT891357
SF15	pH-13	pT-GTW6-CMV-mKate_2xmiR21TS3'	[6]	MT891358
SF10-11, 15	pH-14	pT-GTW6-CMV-mKate_3xmiR21TS3'	[6]	MT891359
SF16	pH-16	pT-GTW6-CMV-mKate_1xmiR221TS5'	[6]	MT891361
SF16	pH-17	pT-GTW6-CMV-mKate_2xmiR221TS5'	[6]	MT891362
SF16, 20-22	pH-18	pT-GTW6-CMV-mKate_3xmiR221TS5'	[6]	MT891363
SF16	pH-20	pT-GTW6-CMV-mKate_1xmiR221TS3'	[6]	MT891364
SF16	pH-21	pT-GTW6-CMV-mKate_2xmiR221TS3'	[6]	MT891365
2h & SF16	pH-22	pT-GTW6-CMV-mKate_3xmiR221TS3'	[6]	MT891366
2a, 4, SF2 & SF3	pGLM49	INS-bGHpA-P_{EF1a}-mCitrine-SV40pA-INS		MT891334
SF3	pGLM177	P_{SV40}-PuroR-SV40pA-INS-bGHpA-P_{EF1a}-sigW-SV40pA-INS	[1]	MT891340
2a, SF2 & SF4	pTTF72	INS-bGHpA-P_{EF1a}-mRuby3-SV40pA-INS	[1]	MT891324
2a, 4, 5e, SF3, SF25 & SF27	pGLM171	AmpR-INS-bGHpA-SV40pA-INS-pUCori	[1]	MT891339
2b	pTTF84	INS-bGHpA-synpA-P_{EF1a}-tTA2::Cerulean-SV40pA-INS-bGHpA-P_{EF1a}-mCitrine-SV40pA	[1]	MT891325
2b	pTTF145	INS-bGHpA-mRuby3-P_{biTRE}-miRFP670-SV40pA-INS	[1]	MT891327
2e & SF7	pTTF218	P_{TRE}-(HDV)mCitrine-SV40pA-INS-bGHpA-P_{EF1a}-tTA2::P2A::mRuby3-SV40pA-P_{SV40}-puΔtk-SV40pA	[1]	MT891330

2e & SF7	pTTF219	P_{TRE}-(dHDV)mCitrine-SV40pA-INS-bGHpA-P_{EF1a}-tTA2::P2A::mRuby3-SV40pA-P_{SV40}-puΔtk-SV40pA	[1]	MT891331
SF4	pTTF57	INS-bGHpA-P_{EFS}-mCitrine-SV40pA-INS	[1]	MT891323
SF4	pTTF181	INS-bGHpA-P_{EFS}-mRuby3-SV40pA-INS	[1]	MT891328
4	pGLM91	INS-bGHpA-P_{TRE}-DsRed(FF4)-TFF4x3-SV40pA-INS		MT891335
4	pGLM92	INS-bGHpA-P_{TRE}-DsRed(FF4)-TFF5x3-SV40pA-INS		MT891336
4b	pGLM103	P_{SV40}-PuroR-SV40pA-INS-bGHpA-P_{EF1a}-tTA::Cerulean-TFF5x3-SV40pA-INS		MT891338
4c	pGLM102	P_{SV40}-PuroR-SV40pA-INS-bGHpA-P_{EF1a}-tTA::Cerulean-TFF4x3-SV40pA-INS		MT891337
5e & SF25	pTTF138	INS-bGHpA-P_{EF1a}-miRFP670-SV40pA-INS	[1]	MT891326
5e & SF25	pTTF220	P_{SV40}-PuroR-SV40pA-INS-bGHpA-P_{EF1a}-mCitrine-TFF5x3-SV40pA-INS-P_{EF1a}-mRuby3(FF4)-TFF5x3-SV40pA-INS	[1]	MT891332
5e & SF25	pTTF223	P_{SV40}-PuroR-SV40pA-INS-bGHpA-P_{EF1a}-mCitrine-TFF4x3-SV40pA-INS-P_{EF1a}-mRuby3(FF4)-TFF4x3-SV40pA-INS	[1]	MT891333
SF4	pTTF194	INS-bGHpA-P_{EF1a}-mCitrine-SV40pA-INS-P_{SV40}-miRFP670-SV40pA-INS	[1]	MT891329

Supplementary Table 3. List of the primers and oligos used to generate miRNA target sites.

Construct	Primer name	Primer Sequence
mKate_1xmiR31TS5'	Fw1	GATCCAGCTATGCCAGCATCTTGCCTG
mKate_1xmiR31TS5'	Rv1	CTAGCAGGCAAGATGCTGGCATAGCTG
mKate_2xmiR31TS5'	Fw2	GATCCAGCTATGCCAGCATCTTGCCTAGCTATGCCAGCATCTTGCCTG
mKate_2xmiR31TS5'	Rv2	CTAGCAGGCAAGATGCTGGCATAGCTAGGCAAGATGCTGGCATAGCTG
mKate_3xmiR31TS5'	Fw3	GATCCAGCTATGCCAGCATCTTGCCTAGCTATGCCAGCATCT
mKate_3xmiR31TS5'	Rv3	TAGCTAGGCAAGATGCTGGCATAGCTG
mKate_3xmiR31TS5'	Fw4	TGCCTAGCTATGCCAGCATCTTGCCTG
mKate_3xmiR31TS5'	Rv4	CTAGCAGGCAAGATGCTGGCATAGCTAGGCAAGATGCTGGCA
mKate_1xmiR31TS3'	Fw5	AGCTTAGCTATGCCAGCATCTTGCCTTTAAT
mKate_1xmiR31TS3'	Rv5	TAAAGGCAAGATGCTGGCATAGCTA
mKate_2xmiR31TS3'	Fw6	AGCTTAGCTATGCCAGCATCTTGCCTAGCTATGCCAGCATCTTGCCTTTAAT
mKate_2xmiR31TS3'	Rv6	TAAAGGCAAGATGCTGGCATAGCTAGGCAAGATGCTGGCATAGCTA
mKate_3xmiR31TS3'	Fw7	AGCTTAGCTATGCCAGCATCTTGCCTAGCTATGCCAGCATCT
mKate_3xmiR31TS3'	Rv7	TAGCTAGGCAAGATGCTGGCATAGCTA
mKate_3xmiR31TS3'	Fw8	TGCCTAGCTATGCCAGCATCTTGCCTTTAAT
mKate_3xmiR31TS3'	Rw8	TAAAGGCAAGATGCTGGCATAGCTAGGCAAGATGCTGGCA
mKate_1xmiR21TS5'	Fw9	GATCCTAGCTTATCAGACTGATGTTGAG
mKate_1xmiR21TS5'	Rv9	CTAGCTCAACATCAGTCTGATAAGCTAG
mKate_2xmiR21TS5'	Fw10	GATCCTAGCTTATCAGACTGATGTTGATAGCTTATCAGACTGATGTTGAG
mKate_2xmiR21TS5'	Rv10	CTAGCTCAACATCAGTCTGATAAGCTATCAACATCAGTCTGATAAGCTAG
mKate_3xmiR21TS5'	Fw11	GATCCTAGCTTATCAGACTGATGTTGATAGCTTATCAGACTGAT

mKate_3xmiR21TS5'	Rv11	AGCTATCAACATCAGTCTGATAAGCTAG
mKate_3xmiR21TS5'	Fw12	GTTGATAGCTTATCAGACTGATGTTGAG
mKate_3xmiR21TS5'	Rv12	CTAGCTCAACATCAGTCTGATAAGCTATCAACATCAGTCTGATA
mKate_1xmiR21TS3'	Fw15	AGCTTTAGCTTATCAGACTGATGTTGATTAAT
mKate_1xmiR21TS3'	Rv15	TAATCAACATCAGTCTGATAAGCTAA
mKate_2xmiR21TS3'	Fw16	AGCTTTAGCTTATCAGACTGATGTTGATAGCTTATCAGACTGATGTTG ATTAAT
mKate_2xmiR21TS3'	Rv16	TAATCAACATCAGTCTGATAAGCTATCAACATCAGTCTGATAAGCTAA
mKate_3xmiR21TS3'	Fw17	AGCTTTAGCTTATCAGACTGATGTTGATAGCTTATCAGACTGAT
mKate_3xmiR21TS3'	Rv17	AGCTATCAACATCAGTCTGATAAGCTAA
mKate_3xmiR21TS3'	Fw18	GTTGATAGCTTATCAGACTGATGTTGATTAAT
mKate_3xmiR21TS3'	Rv18	TAATCAACATCAGTCTGATAAGCTATCAACATCAGTCTGATA
mKate_1xmiR221TS5'	Fw21	GATCCACCTGGCATACAATGTAGATTTG
mKate_1xmiR221TS5'	Rv21	CTAGCAAATCTACATTGTATGCCAGGTG
mKate_2xmiR221TS5'	Fw22	GATCCACCTGGCATACAATGTAGATTTACCTGGCATACAATGTAGATT TG
mKate_2xmiR221TS5'	Rv22	CTAGCAAATCTACATTGTATGCCAGGTAAATCTACATTGTATGCCAGG TG
mKate_3xmiR221TS5'	Fw23	GATCCACCTGGCATACAATGTAGATTTACCTGGCATACAATGT
mKate_3xmiR221TS5'	Rv23	CAGGTAAATCTACATTGTATGCCAGGTG
mKate_3xmiR221TS5'	Fw24	AGATTTACCTGGCATACAATGTAGATTTG
mKate_3xmiR221TS5'	Rv24	CTAGCAAATCTACATTGTATGCCAGGTAAATCTACATTGTATGC
mKate_1xmiR221TS3'	Fw25	AGCTTACCTGGCATACAATGTAGATTTTTAAT
mKate_1xmiR221TS3'	Rv25	TAAAAATCTACATTGTATGCCAGGTA
mKate_2xmiR221TS3'	Fw26	AGCTTACCTGGCATACAATGTAGATTTACCTGGCATACAATGTAGATT TTTAAT
mKate_2xmiR221TS3'	Rv26	TAAAAATCTACATTGTATGCCAGGTAAATCTACATTGTATGCCAGGTA
mKate_3xmiR221TS3'	Fw27	AGCTTACCTGGCATACAATGTAGATTTACCTGGCATACAATGTA

mKate_3xmiR221TS3'	Rv27	CAGGTAAATCTACATTGTATGCCAGGTA
mKate_3xmiR221TS3'	Fw28	GATTTACCTGGCATACAATGTAGATTTTTAAT
mKate_3xmiR221TS3'	Rv28	TAAAAATCTACATTGTATGCCAGGTAAATCTACATTGTATGC
pTTF145	oTF221	GATCGAATTCTTCGAGCTCGGTGCGATCGCGCCACCATGGTAGCAG
pTTF145	oGL71	CTAGATCCGGTGGATCCG
pTTF57	oTF67	GATCGCTAGCATCGATGGGCAGAGCGCACATC
pTTF57	oTF68	GATCGCGGCCGCTTAATTAATCTAGGGCAGTTTATGCTGTGTTCTGG CGGCAAACC
pTTF138	oTF159	ATGCGCGGCCGCGCCACCATGGTAGCAGGTCAT
pTTF138	oTF160	GATCTCTAGAGGCGCGCCCTATCATTAGCTCTCAAGCGCGGTGATCC
pTTF220 & pTTF223	oTF238	GAATTCAAGTTCACTGCCGTATAGGCAGCTAAGAAATTAATTAAGCG GCCGCGCCACCA
pTTF220 & pTTF223	oTF334	CTAGTTCGAAGCATGTTACTACTTGTACAGCTCGTCCATGC

Supplementary Table 4. List of the target sites for miRNAs used in this study [7].

miRNA	Sequence
hsa-miR-31-5p	AGCTATGCCAGCATCTTGCCT
hsa-miR-21	TCAACATCAGTCTGATAAGCTA
hsa-miR-221	AAATCTACATTGTATGCCAGGT
miR-FF4	CCGCTTGAAGTCTTTAATTAAA
miR-FF5	AAGCACTCTGATTTGACAATTA

Supplementary Table 5. List of the primers used for qPCR analyses.

Primer	Function	Sequence (5'-3')
F7	Forward primer for mKate	GGTGTCTAAGGGCGAAGAGC
F8	Reverse primer for mKate	GCTGGTAGCCAGGATGTCGA
qPCR-EGFP-F	Forward primer for EGFP	AAGGGCATCGACTTCAAG
qPCR-EGFP-R	Reverse primer for EGFP	TGCTTGTCGGCCATGATATG
qPCR-18S-F	Forward primer for 18S	GCTTAATTTGACTCAACACGGGA
qPCR-18S-R	Reverse primer for 18S	AGCTATCAATCTGTCAATCCTGTC

Supplementary Table 6. 5x isothermal reaction buffer recipe.

**Component**	**Concentration**
PEG-800	25 %
Tris-HCl, pH 7.5	500 mM
$MgCl_2$	50 mM
DTT	50 mM
dATP	1 mM
dTTP	1 mM
dCTP	1 mM
dGTP	1 mM
NAD	5 mM
To 3 mL with ddH_2O	

Supplementary Table 7. Parameter fits related to Figure 4b,c and Supplementary Note 3.

**Parameter**	**Unit**	**Value**
α_{P_1}	$Arbitrary fluorescence$	3108.52
α_{P_2}	$Arbitrary fluorescence$	28007.3
α_{P_3}	$Arbitrary fluorescence$	2198.66
β_{M_1}	$Unitless$	9275.79
β_{M_2}	$Unitless$	1017.34
β_{M_3}	$Unitless$	709.432
$k_{m_{M_1}}$	ng	0.00400709
γ_{M_2}	$Unitless$	1.35779
γ_{M_3}	$Unitless$	14.5078
θ_{M_1}	$Unitless$	286.573
θ_{M_2}	$Unitless$	126.876
κ	$Arbitrary fluorescence$	17.2643
h	$Unitless$	31

Supplementary Table 8. Parameter fits related to Figure 5c and Supplementary Note 4 endogenous microRNA-based iFFL.

Parameter	Unit	Value
β_{M_1}	$Unitless$	0.746056
β_{M_2}	$Unitless$	32.429
$k_{m_{M_1}}$	$Unitless$	59.104
γ_{M_2}	$Unitless$	0.480552
γ_m	$Unitless$	33.2046
θ_{M_2}	$Unitless$	227.144

Supplementary Table 9. Parameter fits related to Figure 5d and Supplementary Note 4 synthetic microRNA-based iFFL.

**Parameter**	**Unit**	**Value**
β_{M_1}	$Unitless$	1.89289
β_{M_2}	$Unitless$	0.0152265
β_{M_3}	$Unitless$	1.43347e-18
$k_{m_{M_1}}$	$Unitless$	3.21455
γ_{M_2}	$Unitless$	2.04539e-16
γ_{M_3}	$Unitless$	1.91369
θ_{M_2}	$Unitless$	9.80712
θ_{M_3}	$Unitless$	30.272

Supplementary Table 10. Parameter fits related to Supplementary Figure 20 and Supplementary Note 4 endogenous microRNA-based iFFL.

**Parameter**	**Unit**	**Value**
β_{M_1}	$Unitless$	1.01232e-9
β_{M_2}	$Unitless$	0.943493
$k_{m_{M_1}}$	$Unitless$	3.57119
γ_{M_2}	$Unitless$	2.85075e-11
γ_m	$Unitless$	0.173874
θ_{M_2}	$Unitless$	10.9904

Supplementary Table 11. Parameter fits related to Supplementary Figure 22 and Supplementary Note 4 endogenous microRNA-based iFFL.

**Parameter**	**Unit**	**Value**
β_{M_1}	$Unitless$	2.81166
β_{M_2}	$Unitless$	1.95439e-5
$k_{m_{M_1}}$	$Unitless$	2.32439
γ_{M_2}	$Unitless$	1.1604e-13
γ_m	$Unitless$	6.65805
θ_{M_2}	$Unitless$	6.38791

Supplementary Table 12. Parameter fits related to Supplementary Figure 25 and Supplementary Note 4 synthetic microRNA-based iFFL.

**Parameter**	**Unit**	**Value**
β_{M_1}	$Unitless$	9.61413e-16
β_{M_2}	$Unitless$	0.00222615
β_{M_3}	$Unitless$	3.91565e-17
$k_{m_{M_1}}$	$Unitless$	33.6238
γ_{M_2}	$Unitless$	3.19321e-14
γ_{M_3}	$Unitless$	13.4858
θ_{M_2}	$Unitless$	6.45491
θ_{M_3}	$Unitless$	30.2392

Supplementary Table 13. Parameter fits related to Supplementary Figure 17 and Supplementary Note 6 Doxycycline titration.

Parameter	Unit	Value
α_{P_1}	$Arbitrary fluorescence$	8467.86
α_{P_2}	$Arbitrary fluorescence$	11843.7
α_{P_3}	$Arbitrary fluorescence$	34208.2
α_{P_4}	$Arbitrary fluorescence$	7872.41
β_{M_1}	$Unitless$	36149.8
β_{M_2}	$Unitless$	1763.75
β_{M_3}	$Unitless$	2970.3
β_{M_4}	$Unitless$	9847.66
γ_{M_1}	$Unitless$	276.803
γ_{M_2}	$Unitless$	11620.
γ_{M_3}	$Unitless$	1914.02
γ_{M_4}	$Unitless$	13025.9
κ_a	$Arbitrary fluorescence$	0.000376413
κ_i	$Arbitrary fluorescence$	0.402255
h_a	$Unitless$	1
h_i	$Unitless$	1

Supplementary Table 14. Parameter fits related to Supplementary Figure 17 and Supplementary Note 6 HDV.

Parameter	Unit	Value
α_{P_1}	$Arbitrary fluorescence$	2349.49
α_{P_2}	$Arbitrary fluorescence$	5194.24
β_{M_1}	$Unitless$	2.17088
β_{M_2}	$Unitless$	0.389635
γ_{M_1}	$Unitless$	269.809
γ_{M_2}	$Unitless$	308.635
ω_{M_1}	$Unitless$	19.1555
κ_a	$Arbitrary fluorescence$	0.00309458
κ_i	$Arbitrary fluorescence$	1.36083
h_a	$Unitless$	2
h_i	$Unitless$	1

Supplementary Table 15. Parameter fits related to Supplementary Figure 17 and Supplementary Note 6 RNA-binding proteins.

L7Ae:

Parameter	Unit	Value
α_{P_1}	$Arbitrary fluorescence$	41764.7
α_{P_2}	$Arbitrary fluorescence$	2604.06
α_{M_3}	$Arbitrary fluorescence$	16208.
β_{M_1}	$Unitless$	657.816
β_{M_2}	$Unitless$	3508.74
β_{M_3}	$Unitless$	386.641
γ_{M_1}	$Unitless$	142.346
γ_{M_2}	$Unitless$	23.082
γ_{M_3}	$Unitless$	10.0254
ν	$Time^{-1}$	36932.
ρ_{M_1}	$Arbitrary fluorescence^{-1}$	1093.94

Ms2-cNOT7:

Parameter	Unit	Value
α_{P_1}	$Arbitrary fluorescence$	19444.9
α_{P_2}	$Arbitrary fluorescence$	14480.
α_{M_3}	$Arbitrary fluorescence$	1.77696
β_{M_1}	$Unitless$	2115.61
β_{M_2}	$Unitless$	858.958
β_{M_3}	$Unitless$	74.2967
γ_{M_1}	$Unitless$	41.1893
γ_{M_2}	$Unitless$	104.793
γ_{M_3}	$Unitless$	31.3533
ρ_{M_1}	$Arbitrary fluorescence^{-1}$	213.586

Supplementary Table 16. Parameter fits related to Supplementary Figure 17 and Supplementary Note 6 miRNA.

**Parameter**	**Unit**	**Value**
α_{P_1}	$Arbitrary fluorescence$	433.658
α_{P_2}	$Arbitrary fluorescence$	4855.31
β_{M_1}	$Unitless$	992.56
β_{M_2}	$Unitless$	895.094
γ_{M_1}	$Unitless$	2232.66
γ_{M_2}	$Unitless$	518.349
γ_{M_3}	$Unitless$	2.56322
$\lambda_{3'}$	$Unitless$	155.969
$\lambda_{5'}$	$Unitless$	926.904
τ_{1x}	$Unitless$	3.0671
τ_{3x}	$Unitless$	70.1826

References

1. Lillacci, G., Benenson, Y. & Khammash, M. Synthetic control systems for high performance gene expression in mammalian cells. *Nucleic Acids Res.* **46**, 9855–9863 (2018).
2. Siciliano, V. *et al.* Engineering modular intracellular protein sensor-actuator devices. *Nat. Commun.* **9**, 1881 (2018).
3. Matsumiya, M., Tomita, T., Yoshioka-Kobayashi, K., Isomura, A. & Kageyama, R. ES cell-derived presomitic mesoderm-like tissues for analysis of synchronized oscillations in the segmentation clock. *Development* **145**, (2018).
4. Lu, J., Zhang, F. & Kay, M. A. A Mini-intronic Plasmid (MIP): A Novel Robust Transgene Expression Vector In Vivo and In Vitro. *Mol. Ther.* **21**, 954–963 (2013).
5. Wroblewska, L. *et al.* Mammalian synthetic circuits with RNA binding proteins for RNA-only delivery. *Nat. Biotechnol.* **33**, 839–841 (2015).
6. Cella, F., Wroblewska, L., Weiss, R. & Siciliano, V. Engineering protein-protein devices for multilayered regulation of mRNA translation using orthogonal proteases in mammalian cells. *Nature Communications* vol. 9 (2018).
7. Griffiths-Jones, S., Grocock, R. J., van Dongen, S., Bateman, A. & Enright, A. J. miRBase: microRNA sequences, targets and gene nomenclature. *Nucleic Acids Res.* **34**, D140–D144 (2006).
8. Applied Biosystems (2017), Access from URL: http://www3.appliedbiosystems.com/cms/groups/mcb_support/documents/generaldocuments/cms_042380.pdf
