# Supplementary material for: Characterization and mitigation of gene expression burden in mammalian cells
Source: Nat Commun. 2020 Sep 15;11:4641. doi: 10.1038/s41467-020-18392-x (PMC7492461; doi:10.1038/s41467-020-18392-x)
Supplement: Supplementary file 2 — Reporting Summary [file 41467_2020_18392_MOESM2_ESM.pdf]

nature research

Corresponding author(s): Velia Siciliano, Mustafa Khammash

Last updated by author(s): Aug 7, 2020

Reporting Summary

Nature Research wishes to improve the reproducibility of the work that we publish. This form provides structure for consistency and transparency in reporting. For further information on Nature Research policies, see our Editorial Policies and the Editorial Policy Checklist.

Statistics

For all statistical analyses, confirm that the following items are present in the figure legend, table legend, main text, or Methods section.

n/a	Confirmed	
☐	☒	The exact sample size (*n*) for each experimental group/condition, given as a discrete number and unit of measurement
☐	☒	A statement on whether measurements were taken from distinct samples or whether the same sample was measured repeatedly
☐	☒	The statistical test(s) used AND whether they are one- or two-sided<br>*Only common tests should be described solely by name; describe more complex techniques in the Methods section.*
☒	☐	A description of all covariates tested
☒	☐	A description of any assumptions or corrections, such as tests of normality and adjustment for multiple comparisons
☐	☒	A full description of the statistical parameters including central tendency (e.g. means) or other basic estimates (e.g. regression coefficient) AND variation (e.g. standard deviation) or associated estimates of uncertainty (e.g. confidence intervals)
☐	☒	For null hypothesis testing, the test statistic (e.g. *F*, *t*, *r*) with confidence intervals, effect sizes, degrees of freedom and *P* value noted<br>*Give P values as exact values whenever suitable.*
☒	☐	For Bayesian analysis, information on the choice of priors and Markov chain Monte Carlo settings
☒	☐	For hierarchical and complex designs, identification of the appropriate level for tests and full reporting of outcomes
☒	☐	Estimates of effect sizes (e.g. Cohen's *d*, Pearson's *r*), indicating how they were calculated

Our web collection on statistics for biologists contains articles on many of the points above.

Software and code

Policy information about availability of computer code

Data collection	FACS Diva Software - flow cytometry samples acquisition<br>CytExpert Software - flow cytometry samples acquisition v2.3
Data analysis	Flow cytometry data analysis: Cytoflow - Opensource v1.0<br>Statistical analysis: GraphPad Prism v8.0.0<br>qPCR data analysis: Bio-Rad - CFX Managing Software v2.1<br>Flow cytometry data analysis and statistical analysis: Custom R script for automating the gating and compensation work flow and analysis<br>R v4.0.2

For manuscripts utilizing custom algorithms or software that are central to the research but not yet described in published literature, software must be made available to editors and reviewers. We strongly encourage code deposition in a community repository (e.g. GitHub). See the Nature Research guidelines for submitting code & software for further information.

Data

Policy information about availability of data

All manuscripts must include a data availability statement. This statement should provide the following information, where applicable:

- Accession codes, unique identifiers, or web links for publicly available datasets
- A list of figures that have associated raw data
- A description of any restrictions on data availability

All relevant data are included as Source Data and/or are available from the corresponding author on reasonable request. Plasmid sequences are deposited on AddGene and GenBank under the accession codes specified in Supplementary Table 2. Strains and plasmids used in this study are available from the corresponding author on reasonable request. The miRNA target sites were obtained from the miRBase database (http://www.mirbase.org/) and are listed in Supplementary Table

4.

Field-specific reporting

Please select the one below that is the best fit for your research. If you are not sure, read the appropriate sections before making your selection.

- [x] Life sciences
- [ ] Behavioural & social sciences
- [ ] Ecological, evolutionary & environmental sciences

For a reference copy of the document with all sections, see nature.com/documents/nr-reporting-summary-flat.pdf

Life sciences study design

All studies must disclose on these points even when the disclosure is negative.

Sample size	Sample size is determined by FACS and qPCR samples acquisitions and is between 2 and 6 biological replicates. Data acquisition is described in Methods section
Data exclusions	No data was excluded.
Replication	Each experiment was repeated independently at least twice with similar results, with the exception of Supplementary Fig. 2 and condition w/o Mitigation, 1.5 equimolar EGFP to mKate plasmid in Supplementary Fig. 20.
Randomization	The samples were not randomized since position of samples in multi-well plates and order of flow cytometric acquisition is not expected to affect the conclusion.
Blinding	Knowledge of a samples identity does not affect the experimental conclusion.

Reporting for specific materials, systems and methods

We require information from authors about some types of materials, experimental systems and methods used in many studies. Here, indicate whether each material, system or method listed is relevant to your study. If you are not sure if a list item applies to your research, read the appropriate section before selecting a response.

Materials & experimental systems

n/a	Involved in the study	
x		Antibodies
	x	Eukaryotic cell lines
x		Palaeontology and archaeology
x		Animals and other organisms
x		Human research participants
x		Clinical data
x		Dual use research of concern

Methods

n/a	Involved in the study	
x		ChIP-seq
	x	Flow cytometry
x		MRI-based neuroimaging

Eukaryotic cell lines

Policy information about cell lines

Cell line source(s)	HEK293T - ATCC<br>mES E14 - Dr. Maaike Welling<br>H1299 - ATCC<br>U2OS - ATCC<br>HeLa - ATCC<br>CHO-K1 - ATCC
Authentication	None of the cell lines were authenticated.
Mycoplasma contamination	Cell lines were not tested for mycoplasma contamination.
Commonly misidentified lines<br>(See ICLAC register)	HEK293T cells are commonly misidentified as HeLa cells. We use them in our study since they are one of the most prominent cell lines.

Flow Cytometry

Plots

Confirm that:

- [x] The axis labels state the marker and fluorochrome used (e.g. CD4-FITC).
- [x] The axis scales are clearly visible. Include numbers along axes only for bottom left plot of group (a 'group' is an analysis of identical markers).
- [x] All plots are contour plots with outliers or pseudocolor plots.
- [x] A numerical value for number of cells or percentage (with statistics) is provided.

Methodology

Sample preparation	Cells were washed with PBS and detached with trypsin or Accutase solution from the wells. If trypsin was used, the cells were resuspended in media w/o phenol red + 1 % FBS + 2 mM EDTA. Samples were kept on ice or at room temperature until acquisition.
Instrument	FACS Celesta - BD, LSRFortessa SORP - BD or CytoFLEX S - Beckman Coulter
Software	FACS Diva software or CytExpert to collect data<br>Cytoflow or custom R script to analyze
Cell population abundance	About 25-40 % of all events were analyzed following the gating described below.
Gating strategy	Cells were gated in the FSC-A vs SSC-A channels to select living cells (P1). P1 was then gated in FSC-A vs FSC-H channels to select the single cell population. A comparable strategy was used in the custom R script. Here, gating for living cells is preceded by two gating steps designed to remove boundary events and bubbles in the FSC-A vs SSC-A channels. Furthermore, the single cell gate is preceded by a one dimensional threshold gate on the FSC-W channel to remove doublet events. The single cell population is finally refined by an ellipse gate around the point of highest density in the FSC-A vs SSC-A channels. A sample of non-transfected cells or cells transfected with an empty plasmid for each experiment was used to set the positive threshold for each of the fluorescence channels. Cells gated following this pipeline were then analyzed.

- [x] Tick this box to confirm that a figure exemplifying the gating strategy is provided in the Supplementary Information.
